# Modulator Control of Cation Distribution in Mixed-Metal UiO-66(Zr,Ce) Metal–Organic Frameworks

**DOI:** 10.1021/acs.chemmater.5c01629

**Published:** 2025-12-10

**Authors:** Baiwen Zhao, Martin Hutereau, Marc Walker, Reza J. Kashtiban, Paul B. J. Thompson, Ben Slater, Richard I. Walton

**Affiliations:** † Department of Chemistry, University of Warwick, Gibbet Hill Road, Coventry CV4 7AL, U.K.; ‡ Department of Chemistry, 4919 University College London, 20 Gordon Street, London WC1E 6BT, U.K.; § Department of Physics, 2707 University of Warwick, Gibbet Hill Road, Coventry CV4 7AL, U.K.; ∥ XMaS Beamline, European Synchrotron Radiation Facility, Grenoble F-38043, France

## Abstract

The cation distribution and redox activity of the [(Ce_*x*_Zr_6‑x_)­(OH)_4_O_4_]^12+^ clusters in bimetallic UiO-66­(Ce/Zr) MOFs were modified by varying the choice of modulator during synthesis. Extended X-ray absorption spectroscopy (EXAFS) at the Ce and Zr K-edges, *in situ* Fourier-transform infrared (FTIR) spectroscopy, along with density functional theory (DFT) calculations reveal that using benzoic acid as a modulator leads to the preferential formation of the [(*cis*-Ce_2_Zr_4_)­(OH)_4_O_4_]^12+^ cluster in UiO-66 (Ce/Zr) with higher Ce incorporation (33% Ce) than formic acid-modulated synthesis, which predominantly contains a [(CeZr_5_)­(OH)_4_O_4_]^12+^ cluster (17% Ce). Analysis of the reaction solution using Ce K-edge EXAFS and Ce L_III_-edge X-ray absorption near edge structure (XANES) shows how the formation of isolated Ce­(III) in the synthesis solution limits the uptake of Ce­(IV) into the UiO-66 structure for both modulators, but the effect is less for the benzoic acid modulator, explaining the enhanced overall incorporation of Ce. The local metal distribution of Ce and Zr in the *M*_6_ clusters is found to significantly impact the catalytic activity in the benzyl alcohol oxidation reaction using microwave-assisted heating, with the *cis*-Ce_2_Zr_4_ clusters exhibiting higher redox activity than CeZr_5_; this is rationalized by the greater possibility of removing oxide ions that bridge a pair of Ce­(IV) in the cluster to mediate the catalytic transformation. The results show how the choice of modulator during MOF synthesis can influence local distribution of metal cations, providing a subtle way to fine-tune properties beyond the average crystal structure.

## Graphical abstract

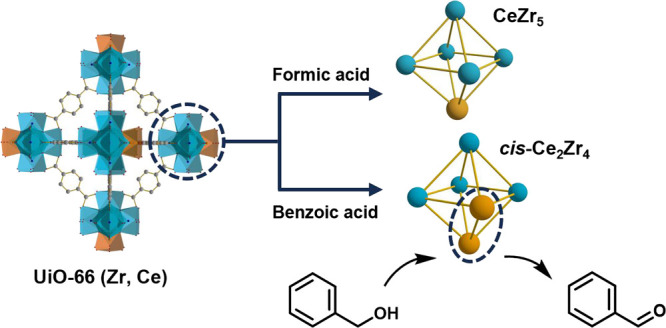

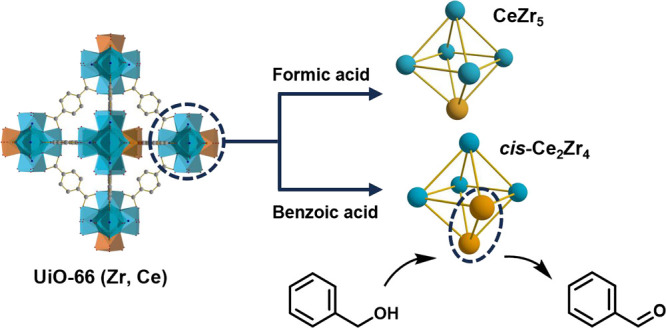

## Introduction

The control of the distribution of metal cations within metal–organic framework (MOF) structures provides a means of modifying properties. Various metal cations may be randomly distributed over a crystal structure, have distinct site preferences that lead to ordering, or may be present in phase segregated regions to give intergrowth structures in the extreme. Examples include controlling the breathing effect of flexible MOFs by mixing two cations,^1^ tuning luminescence properties by the distribution of lanthanide cations in a framework,^2^ and the preparation of core–shell structures with tunable gradients of each metal.^3^ This type of multivariate material, akin to solid-solutions of dense inorganic materials in traditional solid-state chemistry, is challenging to characterize fully because the average crystal structure determination must be complemented by methods that determine the local- and medium-range order, including spectroscopy and microscopy that can probe the location and homogeneity of mixing of two (or more) cations over the parent structure.^4,5^ At the same time, the use of modulating ligands in MOF chemistry has become an established method of modifying crystal size and shape, in influencing defects, and controlling phase selectivity in synthesis.^6^ In this paper, we explore how the choice of modulators can be used to influence the distribution of metal cations in a bimetallic MOF material, illustrated by the well-known material UiO-66 to uncover a principle that may be applicable to other MOF materials to tune their properties for catalysis.

Mixed CeO_2_–ZrO_2_ oxides are classical support materials that have been extensively studied for industrial applications, such as three-way catalysis, soot combustion, oxidation of volatile organics, and water–gas shift reactions.^7,8^ These are well-established in heterogeneous catalysis due to the reversible redox behavior of the Ce^4+^/Ce^3+^ couple that gives rise to oxygen storage properties.^9^ A notable limitation of CeO_2_–ZrO_2_ in catalysis is that only the surface of the crystallites is active in the reaction, leaving a major portion of the cerium atoms within the lattice unused. An effective solution involves the synthesis of structures with dispersed Ce/Zr-oxo clusters, enhancing active site accessibility. In this respect, MOFs offer opportunities, enabling the coordination of mixed Ce/Zr secondary building units (SBUs) with carboxylate ligands resulting in materials that possess high surface area and more accessible active sites.^10,11^ UiO-66 is a well-known MOF constructed from hexanuclear [*M*_6_(μ_3_-O)_4_(μ_3_–OH)_4_]^12+^ building units (*M* = tetravalent metal such as Zr, Ce,^12,13^ Hf,^14,15^ and Ti^16,17^ in mixed-metal analogues) connected in three dimensions by benzene-1,4-dicarboxylate (BDC) ligands to yield a porous network that shows unusual structural stability toward temperature and a range of solvents.^18,19^ The incorporation of Ce into the parent Zr UiO-66 introduces redox capabilities,^20^ addressing the lack of redox chemistry for Zr, therefore making it highly suitable for catalytic applications such as ciprofloxacin degradation,^21^ oxidation of cyclohexane,^22^ reduction of NO,^23^ and acting as a peptidase and oxidase mimic.^24^

The synthesis of bimetallic Ce_*x*_Zr_6‑x_-UiO-66 (0 < *x* < 6) has been reported using solvothermal methods.^25,26^ Monocarboxylic acid modulators are commonly used in the synthesis of MOFs,^6^ and this approach can significantly influence factors such as particle size,^27^ morphology,^28^ defectivity,^29^ and phase formation.^30^ In the synthetic method reported by the Stock group,^26^ formic acid was used as modulator for Ce_*x*_Zr_6‑x_-UiO-66 synthesis, and it was observed that Zr^4+^ cations exhibit a preference for incorporation into the MOF structure, resulting in a significantly lower Ce/Zr ratio compared to the initial composition of the starting materials. This discrepancy, however, was not addressed or explained in the previous work, and presumably, the missing Ce remains in the reaction solution after synthesis. Notably, the effect of using different types of modulators has also not been investigated in the synthesis of the bimetallic Ce_*x*_Zr_6‑x_-UiO-66 system, which may be important for determining the uptake of different metal cations in the UiO-66 structure.

Characterizing mixed-metal UiO-66 requires examining the local atomic distribution of the *M*_6_ clusters. This is crucial because there may be regions with segregated clusters of solely Ce or Zr, or genuine bimetallic clusters as desired, containing both cations with distinct distributions. Recent studies using element selective EXAFS (Ce and Zr K-edge) spectroscopy,^31^ and FTIR spectroscopy,^32^ have demonstrated that UiO-66­(Ce/Zr), synthesized with formic acid as modulator, exhibits a strong tendency to contain three distinct types of clusters: Ce_6_, CeZr_5_, and Zr_6_. These studies revealed that bimetallic CeZr_5_ clusters are preferably formed and coexist with pure Zr_6_ clusters when the cerium content is below 1/6 (16.7%), or with pure Ce_6_ clusters when the cerium content exceeds this threshold.

In this work, we investigate the tunability of the synthesis of UiO-66­(Ce/Zr) through variation of modulator and examine the composition of the hexanuclear clusters of the synthesized Ce_*x*_Zr_6‑x_-UiO-66 by EXAFS and *in situ* FTIR spectroscopy, rationalized by DFT calculations. While previous work on the use of modulators in the crystallization of UiO-66-type MOFs has focused on the influences of modulator on crystallinity, yield, morphology, pore size, defects, porosity, stability, and gas separation performance,^33–36^ by changing the modulator from formic acid to benzoic acid, we show that it is possible to tailor the local distribution of Ce^4+^ and Zr^4+^ cations. In particular, we are able to form clusters of Ce_2_Zr_4_ composition within the framework, with the pair of Ce centers in a *cis* orientation. When tested for catalytic oxidation of benzyl alcohol, these materials exhibit redox activities that depend on the presence of specific cluster compositions. Our work provides a strategy not only for controlling the distribution of metal cations in a MOF structure but also in influencing the bulk properties of the resulting material.

## Results and Discussion

### Synthesis and Characterization

The synthesis of the mixed-metal UiO-66­(Ce/Zr) using different modulators with low to high Ce/Zr ratios was optimized based on a published procedure using solvothermal conditions (using *N*,*N*- dimethylformamide (DMF) – water mixed solvent).^26^ The detailed synthesis methods are provided in Supporting Information (SI; Section S2). The synthesized samples are designated as FA-CeXX and BA-CeXX, where FA represents formic acid, BA represents benzoic acid and XX denotes the intended Ce content (mol %) from the ratio of metal precursor salts. Single-metal UiO-66­(Ce) and UiO-66­(Zr) were also synthesized for comparison. Powder XRD (PXRD) patterns of the as-synthesized UiO-66 (Ce/Zr) via formic acid (Figure 1a) and benzoic acid (Figure 1b) confirms successful synthesis of phase-pure samples without phase separation, the absence of any unreacted ligand precursor, and no additional peaks indicative of ordering of missing clusters defects.^37^ Increasing Ce^4+^ incorporation causes gradual shifts toward lower diffraction angles, consistent with the larger ionic radius of Ce^4+^. The powder XRD patterns (Figures S1–S6) are fitted using the Pawley method with a cubic unit cell in the *Fm*3̅*m* space group to determine the lattice parameters *a*_*Ce*/*Zr*_ (Table S4) followed by the estimation of the content of Ce^4+^ and Zr^4+^ ions according to Vegard’s law. DFT modeling confirms that this approach is valid since the unit cell volume of UiO-66­(Ce/Zr) scales linearly with cerium content (Figure S51). The Pawley plots of FA-Ce50 and BA-Ce50 are shown in Figure 1c and d, respectively. The PXRD analysis shows no evidence for a change of symmetry from the ideal cubic (*Fm*3̅*m*) symmetry expected for the UiO-66 structure,^18^ which implies no crystallographic ordering of any defects or of the Ce^4+^ and Zr^4+^ cations over the long-range structure. Since the crystallographic description of UiO-66 has only one unique site for the metal cation, it is not possible to refine the distribution of cations within the clusters, and a local spectroscopic probe is instead needed (see below for the EXAFS analysis).

**Figure 1:**
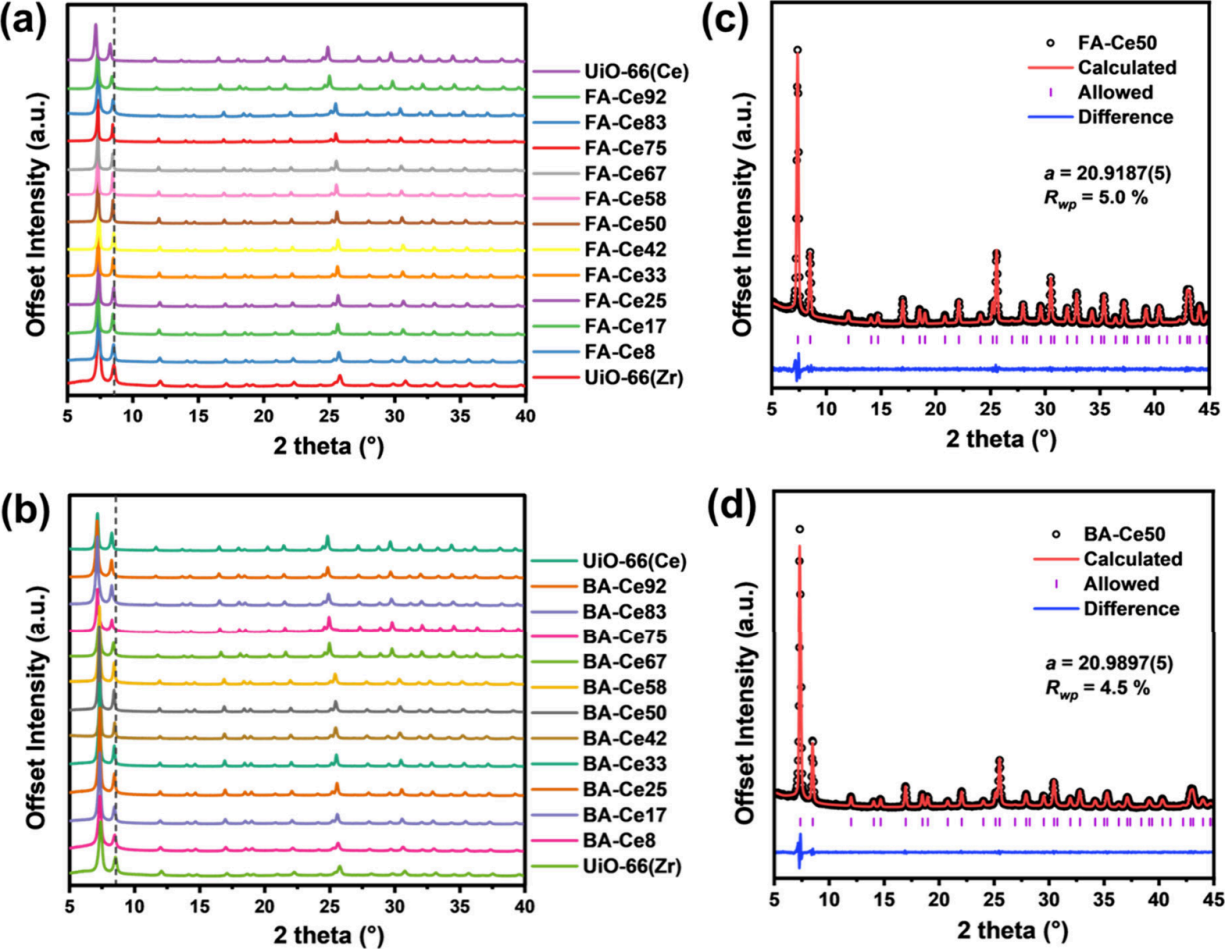
Powder XRD patterns of UiO-66­(Ce/Zr) synthesized with various Ce proportions using formic acid (a) and benzoic acid (b) as modulators compared with pristine UiO-66­(Ce) and UiO-66­(Zr). The dotted lines indicate the comparison of peak positions across different patterns, showing the increase in unit cell parameter with increasing Ce content. Powder XRD patterns were refined using the Pawley fit (Space group *Fm*3̅*m*) of samples FA-Ce50 (c) and BA-Ce50 (d).

Thermogravimetric analysis (TGA) (Figures S8, S9) shows how higher Ce^4+^ incorporation leads to a lower onset of decomposition to collapse under thermal treatment, consistent with the literature.^26^ TGA (Figures S11–S16) also enables the quantification of missing linker defects (Table S6) by comparing the mass loss including the decomposition of the BDC ligands and the modulators along with the calculated mass loss for a nondefective structure. It was thus found that missing linker defects are notably similar for both the BA- and FA-modulated samples. The modulator:BDC ratio was separately determined using solution NMR of digested MOFs (Table S7), while the Ce/Zr ratios were confirmed by X-ray fluorescence (XRF) spectroscopy (Table S4), which shows good agreement with the trend in lattice parameters from PXRD. The PXRD analysis thus provides independent evidence for the Ce/Zr ratios. Figure 2 presents the elemental composition results from XRF for materials prepared by formic acid and benzoic acid modulated syntheses, which reveals a different modulator influence on the final Ce content (mol %) in UiO-66­(Ce/Zr) MOFs. As the targeted Ce concentration increases, the Ce content in the MOF initially increases comparably for both modulators. The observed plateaus indicate a maximum achievable Ce content under certain initial Ce/Zr ratios. In the formic acid modulated synthesis, the observed 20% plateau aligns well with the previously reported formation of CeZr_5_ SBUs (17% Ce).^31^ However, with benzoic acid, the Ce proportion plateaus at a higher value of approximately 34%. This suggests the potential formation of clusters with a higher Ce content. Upon exceeding the plateaux, the formation of Ce_6_ clusters becomes favorable, leading to substantially higher Ce contents in the MOF.

**Figure 2:**
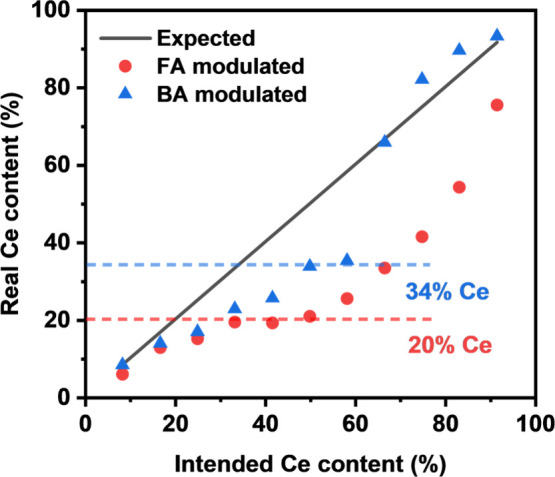
Comparison of the trend of actual and target Ce content (in mol) in UiO-66­(Ce/Zr) synthesized with formic acid (BA) and benzoic acid (FA) as modulators. The real Ce content is derived from XRF, but also verified from powder XRD (see text).

Scanning transmission electron microscopy, energy dispersive X-ray (STEM-EDX) mapping, and line scans of FA-Ce50 (Figure 3a) and BA-Ce50 (Figure 3c) demonstrate that Ce and Zr elements are homogeneously dispersed, with no indications of element segregation, while further TEM images (Figures S17, S18) show that both FA-Ce50 and BA-Ce50 have similar particle sizes of approximately 100 nm. Ce L_III_-edge XANES spectra and Ce and Zr K-edge EXAFS spectra were collected to analyze the local atomic environment in all materials. Normalized Ce L_III_-edge XANES show that Ce in all compounds is present predominantly as Ce (IV) oxidation state, as compared to Ce­(IV) (CeO_2_) and Ce­(III) (CeAlO_3_) reference materials. The materials synthesized using FA (Figure 3b) and BA (Figure 3d) with varying Ce/Zr ratios show minimal differences in their spectra. This suggests that the local chemical environment of Ce is largely unaltered, regardless of Ce content, indicating isomorphous substitution of Zr­(IV) by Ce­(IV). Consequently, the presence of Ce­(III) is ruled out in our materials, which others have shown can be accommodated by UiO-66­(Ce) via specific synthesis conditions.^38^

**Figure 3:**
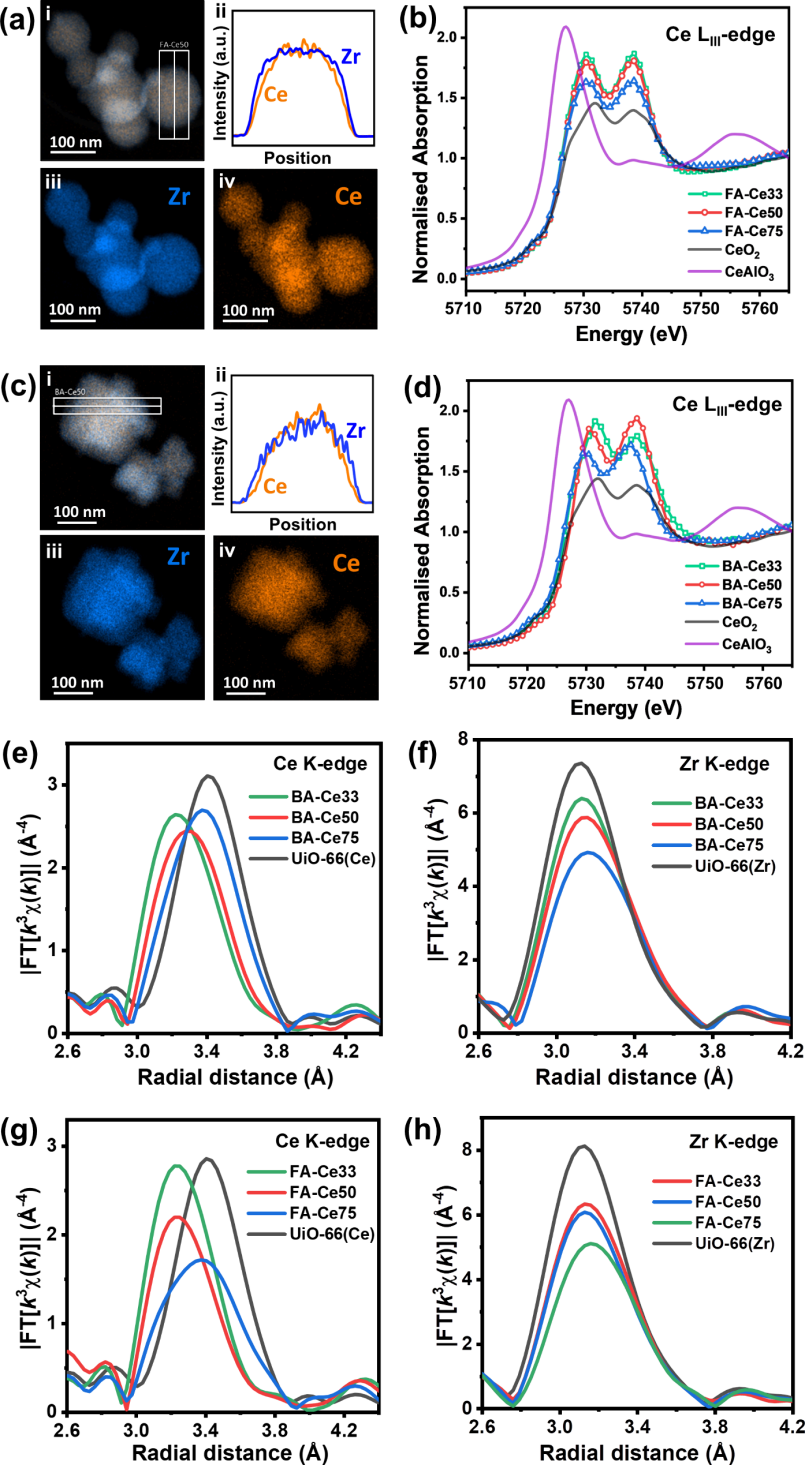
(a) STEM-EDX analysis for FA-Ce50: (i) mixed Ce/Zr composite map, (ii) EDX scan along the line of (i), (iii) Zr map, and (iv) Ce map. (b) Ce L_III_-edge XANES spectra of FA-modulated samples compared to CeO_2_ and CeAlO_3_. (c) STEM-EDX analysis for BA-Ce50: (i) mixed Ce/Zr composite map, (ii) EDX scan along the line of (i), (iii) Zr map, (iv) Ce map. (d) Ce L_III_-edge XANES spectra of BA-modulated samples compared to CeO_2_ and CeAlO_3_. (e) *k* ^3^-weighted, Fourier-transformed Ce K-edge EXAFS for FA-modulated samples. (f) *k* ^3^-weighted, Fourier-transformed Zr K-edge EXAFS for FA-modulated samples. (g) Fourier-transformed Ce K-edge EXAFS for BA-modulated samples. (h) Fourier-transformed Zr K-edge EXAFS for BA-modulated samples.

EXAFS spectra at the Ce K-edge and Zr K-edge (Figure S19) were used to quantify the local atomic arrangement about each atom type in the absence of long-range crystallographic order. The Fourier transforms of the Ce K-edge (Figure 3e,g) and Zr K-edge EXAFS (Figure 3f,h) reveal shifts of the second features that are due to Ce-*M* (*M* = Ce or Zr) scattering within the Ce_*x*_Zr_6–x_ clusters (2.8–4.0 Å) toward higher *R* values. The observed shifts in radial distance, along with changes in Fourier transform intensity, indicate modifications in Ce-*M* and Zr-*M* distances and variations in the coordination environment, suggesting different Ce_*x*_Zr_6–x_ cluster compositions.

Focusing on the composition of the Ce_*x*_Zr_6‑x_ clusters, fitting of the Fourier transforms was performed over the region corresponding to *M-M* scattering, specifically R = 3.0–3.8 Å for the Ce K-edge and R = 2.8–3.8 Å for the Zr K-edge spectra. This follows the approach of Lomachenko et al.^31^ who studied FA-modulated mixed-metal Ce/Zr UiO-66. To ensure consistency in the fitting parameters, the amplitude reduction factor (*S*_0_^2^) was initially confirmed to be 0.9 for the Ce K-edge and 1.5 for the Zr K-edge using pure Ce and Zr UiO-66 reference samples (Figure S20) and then fixed at these values for analysis of the mixed-metal materials. Detailed fitting parameters are provided in Table S8. Spectra from both samples were fitted using an octahedral *M*_6_ cluster model with a shell of 4 nearest Ce or Zr neighbors. The Debye–Waller (DW) factors (σ^2^) for UiO-66­(Zr) and UiO-66­(Ce) are 0.007 Å^2^ and 0.006 Å^2^, respectively, indicating similar levels of structural disorder, while the obtained Zr–Zr (3.53 Å) and Ce–Ce (3.79 Å) distances of UiO-66­(Zr) and UiO-66­(Ce) are consistent with both those from DFT (3.51 Å and 3.80 Å, respectively) and values reported previously.^31,39^ To investigate the local atomic distribution of Ce_*x*_Zr_6‑x_ MOFs synthesized with different modulators, we first replicated the fitting of FA-modulated materials (Figure 4a,b,c) using the CeZr_5_ model (along with Ce_6_ or Zr_6_ coexisting to account for the overall Ce content in the materials). The *R*(Ce–Zr) interatomic distance of the FA-modulated materials is fitted to approximately 3.65 Å, which aligns with the literature.^40^ Additionally, the Ce K-edge spectra indicate that as the Ce content increases, the DW factor for the Ce–Zr bond also increases, suggesting that the CeZr_5_ cluster undergoes distortion with higher Ce incorporation. This provides indicative parameters for subsequent analyses of BA-modulated materials.

**Figure 4:**
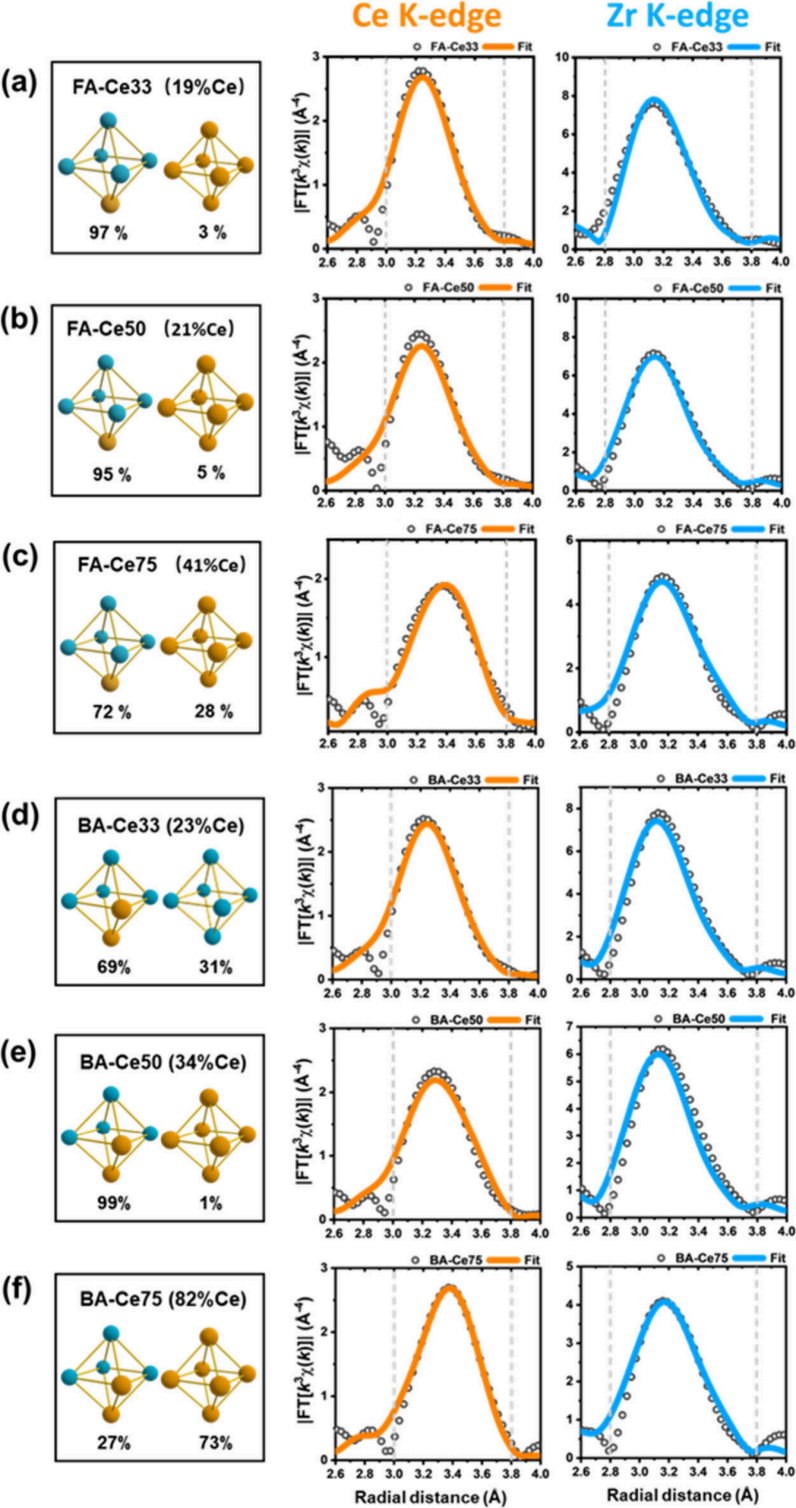
Results of the final EXAFS fitting for the formic acid and benzoic acid modulated samples: (a) FA-Ce33, (b) FA-Ce50, (c) FA-Ce75, (d) BA-Ce33, (e) BA-Ce50 and (f) BA-Ce75 at the Ce (middle panels) and Zr (right panels) K-edges. The left panels show the cluster configurations (orange spheres are Ce and blue are Zr) used in the fitting of the Fourier transform of the EXAFS shown on the right. The fitting range is indicated by gray dashed lines.

For the BA-modulated samples, various possible cluster compositions were considered to provide different models to test via the EXAFS fitting process (Section S6.3.2). This follows the procedure of including a predominantly mixed-metal cluster together with Ce_6_ or Zr_6_ clusters to add up the overall Ce/Zr ratios in the materials. Comparing the fitting parameters and results from these models allowed for the determination of the most probable metal distribution within the clusters. Specifically, the fitting parameters from pure Ce_6_/Zr_6_, CeZr_5_, *trans-*/*cis*-Ce_2_Zr_4_, *fac*-/*mer*-Ce_3_Zr_3_ models were respectively evaluated (Section S6.3). The analysis shows that the *cis*-Ce_2_Zr_4_ model (Figure 4d-f) uniquely provides the best agreement with the experimental data for BA-Ce33, demonstrating the most accurate and robust fitting parameters, all with physically reasonable values (Table 1). While the fitted parameters from the Zr K-edge are very similar for both models, the Ce K-edge spectra reveal significant distinctions. The CeZr_5_ model yields a higher *R*(Ce–Ce) value and a much lower DW factor (σ_Ce–Ce_^2^ = 0.002 Å^2^) compared to the *cis*-Ce_2_Zr_4_ model. This deviated *R*(Ce–Ce) distance (3.83 Å) suggests that there is a need for additional paths of Ce–Ce scattering in the CeZr_5_ model. The *cis*-Ce_2_Zr_4_ model effectively addresses this issue, with Ce–Ce distances that closely align with the measured data (3.80 Å), and a DW factor (σ_Ce–Ce_^2^ = 0.009 Å^2^) that falls within a more reasonable and consistent range compared to other samples. This is in agreement with DFT simulations of mixed Ce–Zr UiO-66 in the conventional unit cell (4 SBUs; 24 metal atoms). For the CeZr_5_ model, Ce–Ce paths are possible only in the Ce_6_ nodes that make up the cerium content. With the *cis*-Ce_2_Zr_4_ model, an additional path is possible with a reduced Ce–Ce distance (Table S19) as the cerium atoms in the mixed building block are held closer than ideal by shorter bonds between Zr and bridging oxygens. We also note that the successful analysis of the EXAFS spectra provides further evidence for the Ce:Zr ratio in the materials.

**Table 1:** EXAFS Fitting Parameters of BA-Ce33, BA-Ce50 and BA-Ce75 at the Ce and Zr K-Edges Using the *cis*-Ce_2_Zr_4_ Model, *S*_0_^2^ = 0.9 (Ce K-Edge) and *S*_0_^2^ = 1.5 (Zr K-Edge)

Sample	Edge	Shell	ΔE/eV	*R*/Å	σ^2^/Å^2^	R-factor
BA-Ce33 (23% Ce)	Ce K	3 Zr	–5.9 ± 3.2	3.654 ± 0.006	0.005 ± 0.0007	0.05
		1 Ce		3.800 ± 0.005	0.009 ± 0.0009	
	Zr K	2.96 Zr	–5.2 ± 2.4	3.515 ± 0.010	0.006 ± 0.0008	0.11
		1.04 Ce		3.661 ± 0.012	0.009 ± 0.0004	
BA-Ce50 (34% Ce)	Ce K	2.97 Zr	–4.9 ± 1.8	3.648 ± 0.006	0.006 ± 0.0011	0.08
		1.03 Ce		3.806 ± 0.007	0.005 ± 0.0005	
	Zr K	2.51 Zr	–3.4 ± 3.1	3.522 ± 0.011	0.005 ± 0.0017	0.12
		1.49 Ce		3.649 ± 0.008	0.010 ± 0.0012	
BA-Ce75 (82% Ce)	Ce K	0.81 Zr	–8.9 ± 3.7	3.629 ± 0.004	0.003 ± 0.0006	0.03
		3.19 Ce		3.814 ± 0.005	0.007 ± 0.0008	
	Zr K	2.5 Zr	–2.7 ± 2.0	3.536 ± 0.014	0.008 ± 0.0010	0.11
		1.5 Ce		3.662 ± 0.008	0.008 ± 0.0012	

*In-situ* FTIR spectroscopy (Section S7) on heating can probe the structure of [Ce_*x*_Zr_6–*x*_(μ_3_-O)_4_(μ_3_–OH)_4_]^12+^ clusters by examining the ν­(OH) bands of bridging μ_3_–OH groups, which are sensitive to the distribution of metal cations.^41^ Samples were heated to 110 °C under an Ar flow to remove loosely bound water molecules, and data were collected *in situ* until the OH stretching from water at 3300 cm^–1^ was eliminated, ensuring clear observation of the μ_3_–OH features. In the [Ce_*x*_Zr_6–*x*_(μ_3_-O)_4_(μ_3_–OH)_4_]^12+^ clusters, four possible types of μ_3_–OH groups can be identified by their vibrational bands: (μ_3_–OH)­Ce_3_, (μ_3_–OH)­CeZr_2_, (μ_3_–OH)­Ce_2_Zr, and (μ_3_–OH)­Zr_3_. A comparison of the ν­(OH) region (3500–3750 cm^–1^) in the IR spectra (Figure 5) for BA-Ce50 and FA-Ce50 compounds against pure Ce and Zr UiO-66 materials reveals significant differences. For FA-Ce50, predominantly containing CeZr_5_ clusters, the μ_3_–OH vibrations are seen at lower wavenumbers compared to UiO-66­(Zr), indicating the presence of mixed (μ_3_–OH)­Zr_3_ and (μ_3_–OH)­CeZr_2_ groups, consistent with previous literature.^32^ The ν­(OH) peak in BA-Ce50 shifts further to lower wavenumbers, indicating the presence of additional (μ_3_–OH)­Ce_2_Zr species. This configuration can occur only when there are two adjacent Ce atoms in a hexanuclear cluster. This provides independent experimental evidence for the formation of *cis*-Ce_2_Zr_4_ clusters, entirely consistent with the EXAFS analysis.

**Figure 5:**
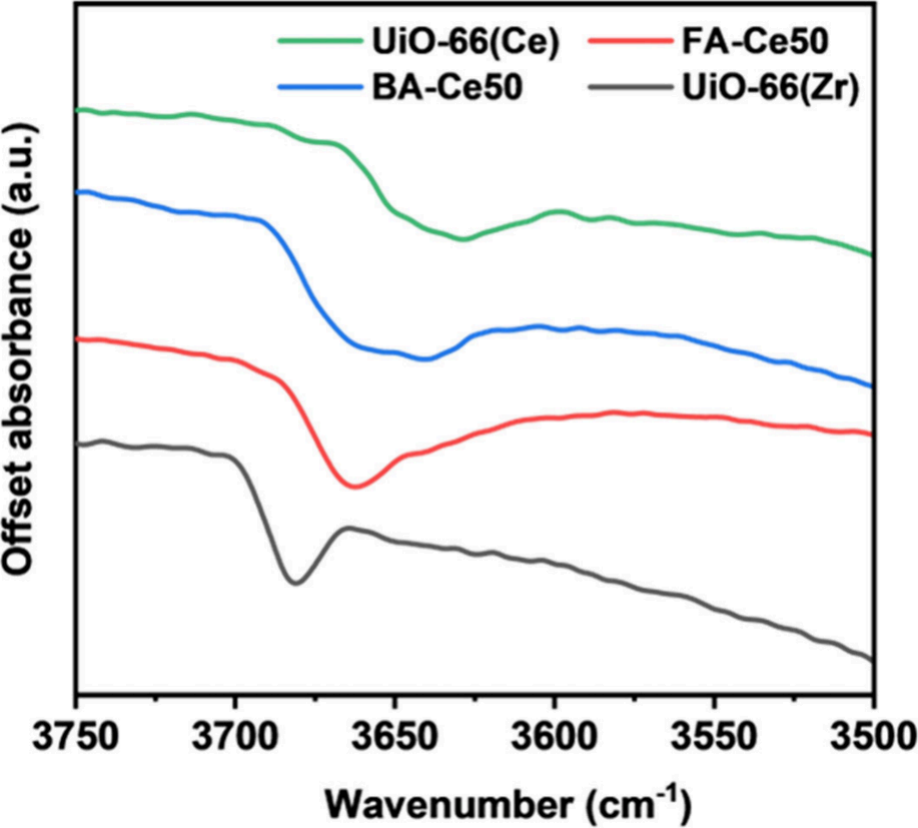
Comparison of the IR spectra ν­(OH) region (3500- 3750 cm^–1^) for samples BA-Ce50, FA-Ce50 against pristine UiO-66­(Ce) and UiO-66­(Zr).

It is not clear why certain types of building blocks are preferred or why this differs depending on the modulator. DFT calculations were carried out on isolated clusters (Figure S49) to evaluate the relative energies for different Ce_*x*_Zr_6‑x_ compositions. We find that Zr–Ce edges in the octahedral core of the SBU are unfavorable, as found also by Trousselet et al.^40^ This should favor systems in which the number of mixed edges is minimized, such as CeZr_5_, *cis*-Ce_2_Zr_4_, and *fac*-Ce_3_Zr_3_ (See Figure S48). Nevertheless, most configurations lie within thermal energy (2–3 kJ mol^–1^) of each other, whether terminated by FA or by BA, which disfavors a thermodynamic driving force behind the formation of CeZr_5_ SBUs and instead suggests that clusters with cerium contents from 0 to 6 are likely to be seen. Similar calculations were carried out on the defect-free periodic material, as shown in Figure 6. The difference in relative energies of these mixed systems is significantly larger, as the difference in ionic radii between Zr and Ce leads to a mismatch in the lengths of bonds between metals and linker oxygen atoms. Given the high connectivity of nodes in UiO-66 and the resulting stiffness of the framework via the rigid BDC linker, there is little scope for relaxing the structure to mitigate the strain that this induces. We find that it is thermodynamically preferable to incorporate a given cerium content in the material as Ce_6_ SBUs rather than as separate CeZr_5_ or Ce_2_Zr_4_ units. Taking missing-linker defects into account does not change this overall trend (SI, Section S11.3), but it decreases the energy of mixed systems relative to phase-pure Zr or Ce UiO-66, which may offer a means of influencing the metal distribution. It is therefore likely that kinetic or mechanistic factors ultimately control which SBUs predominantly form or assimilate into the material. While progress has been made in recent years on the crystallization pathways of UiO-66­(Zr), there is no consensus on a mechanism, and the possible influence of the cerium precursor is not known.^6,42–44^

**Figure 6:**
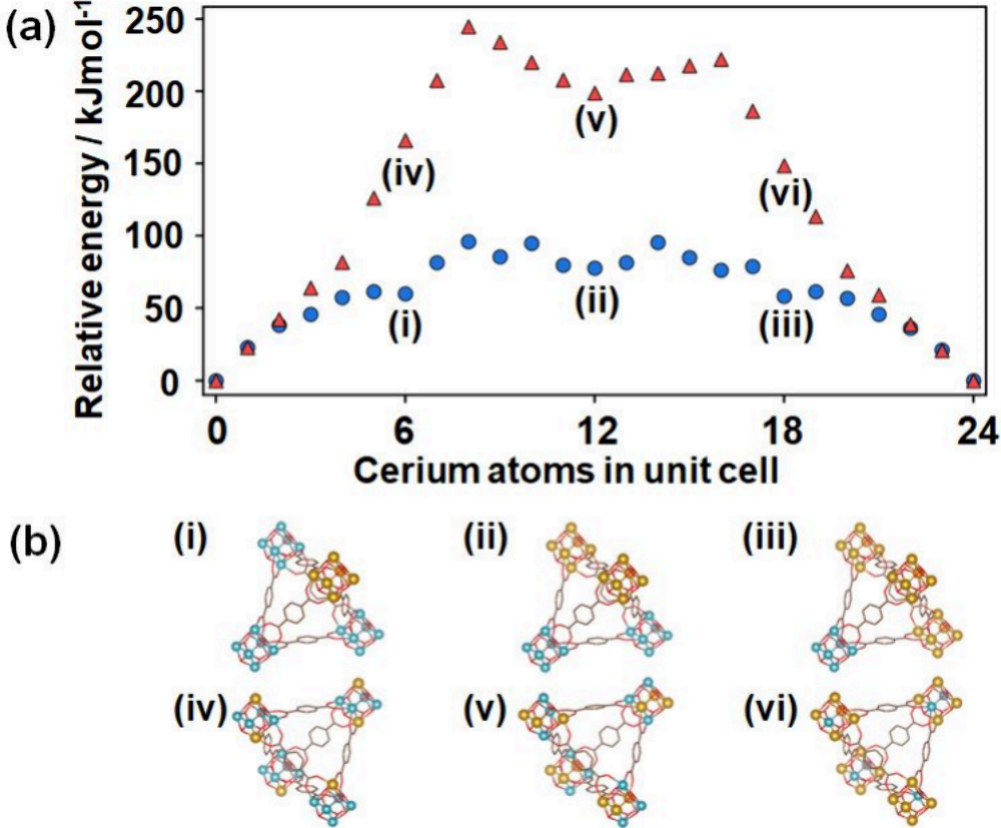
(a) DFT energies of unit cells of UiO-66 with different cerium contents. Energies were defined relative to the phase-pure Zr-UiO-66 and Ce-UiO-66 materials. Blue circles correspond to configurations in which a given cerium content is incorporated in a manner that minimizes the average distance between cerium atoms; red triangles correspond to those in which the average distance between cerium atoms is maximized. (b) Schematics (i)-(vi) showing the types of SBUs present in these structures; Zr and Ce are drawn blue and orange, and not all linkers are shown. More detail can be found in section S11.3.

To understand the reason for the limited inclusion of Ce into the MOF materials, we used XAFS spectroscopy to analyze the reaction solution *in situ* (Section S6.4, Supporting Information). The crystallization of the MOF is accompanied by the competing process of the reduction of the Ce­(IV) precursor ((NH_4_)_2_Ce­(NO_3_)_6_) to Ce­(III) which is evidenced by the Ce L_III_-edge XANES spectra of the synthesis solution before and after the reaction for FA-Ce50 (Figure S23) and BA-Ce50 (Figure S24). The reduction of Ce­(IV) occurs in DMF even at room temperature (Figure S25). Fourier-transformed *k* ^*3*^-weighted Ce K-edge EXAFS spectra (Figure 7) of the reaction solution after synthesis shows the presence of monomeric Ce­(III) in the remaining solution, with no evidence of Ce-containing clusters, as indicated by the lack of a Ce–*M* scattering signal. Each cerium atom was fitted with a shell of nine oxygen atoms, resulting in an average Ce–O distance of approximately 2.516 Å for FA-Ce50 and 2.522 Å for BA-Ce50 solution samples (Table S15). The Ce–O distance agrees with the reported value of 2.5232 Å from literature for Ce­(III) formate.^45^ It can be concluded that during synthesis, the stability of Ce­(III) species in specific solution/modulator environments limits the amount of Ce­(IV) that can be incorporated into the *M*_*6*_ clusters. This is in agreement with a series of DFT calculations on monomeric Ce­(III) complexes, which show that the incorporation of this species in *M*_*6*_ clusters is highly energetically unfavorable for both modulators (see Section S11.5). For the FA-modulated syntheses, it is noteworthy that with extended synthesis times, a crystalline byproduct of Ce­(III) formate is produced (Figure S7). This highlights the competition between the inclusion of Ce­(IV) in the MOF structure and formation of Ce­(III) in solution. This also explains why a short reaction time is needed to prepare UiO-66 that contains Ce, as first reported by Stock and co-workers.^26^ These considerations highlight that kinetic effects are also important to rationalize the limited uptake of Ce, and how this differs between differently modulated materials. The kinetic effects are likely to outweigh the energetics of stability of different clusters and any entropic gain of mixing of the two cations. This would be in line with the DFT calculations on the Zr–Ce aperiodic and periodic models, which revealed that there is no clear thermodynamic driving force behind the formation of such systems.

**Figure 7:**
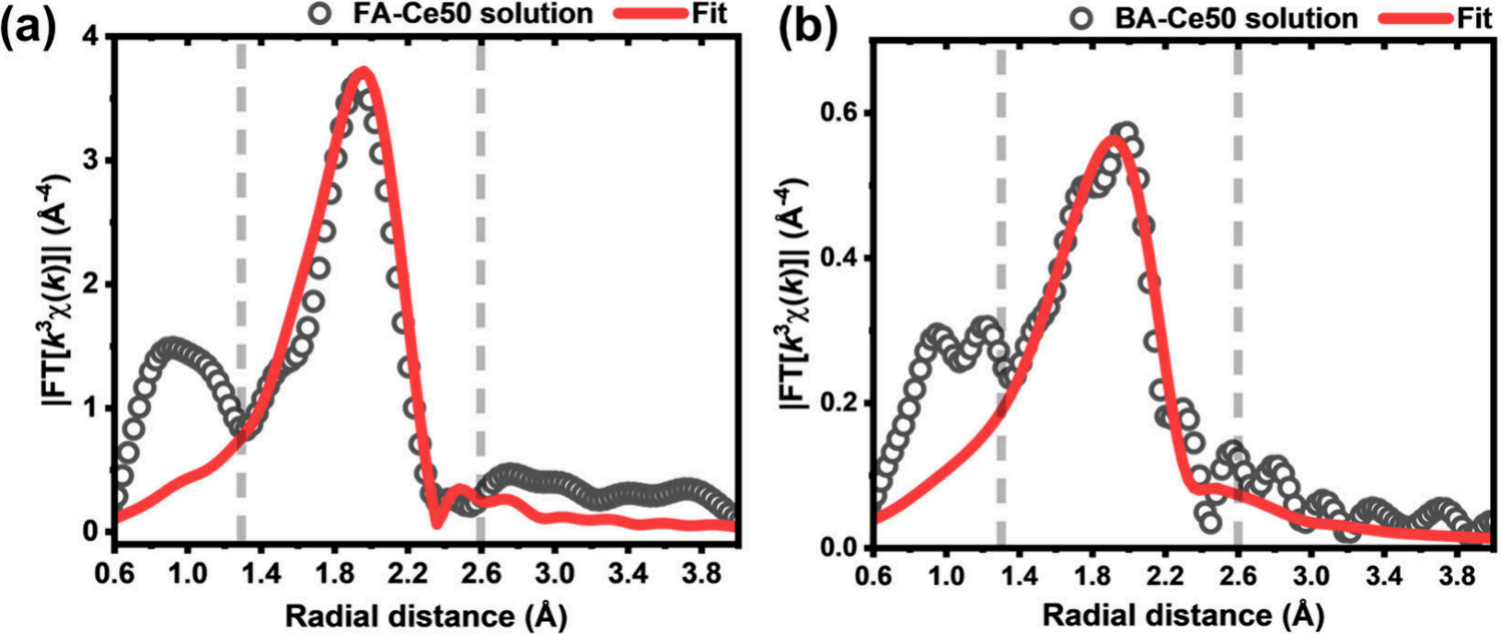
*k* ^3^-weighted, Fourier-transformed Ce K-edge EXAFS data of the synthesis solution after reaction for sample FA-Ce50 (a) and BA-Ce50 (b); fitted ranges are shown as gray dashed lines with R = 1.3–2.6 Å.

Considering the effects of solvents and modulators (Table S5), our synthesis using a 50/50 Ce/Zr ratio of reagents indicates that the combination of DMF and benzoic acid yields the highest Ce content (34%) in the MOF. These experiments included variations using DEF (*N*,*N*-diethylformamide), DMA (*N*,*N*-dimethylacetamide), and DMF as solvents and formic acid, acetic acid, and benzoic acid as modulators. We conclude that the solvent and modulator effects can be considered as important tools in tuning the local metal distribution in mixed-metal UiO-66 MOFs.

### Catalytic Oxidation

The BA- and FA-modulated UiO-66 (Ce/Zr) materials were examined for as catalysts for the selective oxidation of benzyl alcohol to benzaldehyde (Scheme 1), as a test for redox catalytic activity due to benzyl alcohol’s reactivity and minimal side product formation.^46^ The oxidation of such nonactivated primary alkyl C–H bonds is challenging due to their chemical inertness,^47^ thereby allowing the redox activity of different compositions of Ce_*x*_Zr_6‑x_ clusters to be evaluated under controlled conditions. Although adding cocatalysts such as TEMPO ((2,2,6,6-tetramethylpiperidin-1-yl)­oxyl) typically enhances catalytic performance,^48,49^ this was excluded in this test to reduce complexity and ensure exclusively the reactivity of the Ce_*x*_Zr_6‑x_ clusters was measured. Microwave-assisted heating was selected for the reaction to heat rapidly and to control precisely the reaction time to 10 min, while the benzyl alcohol conversion was analyzed by quantitative ^1^H NMR spectroscopy (Figures S37–S47). The analysis showed no detectable side-products such as benzoic acid.

**Scheme 1:**
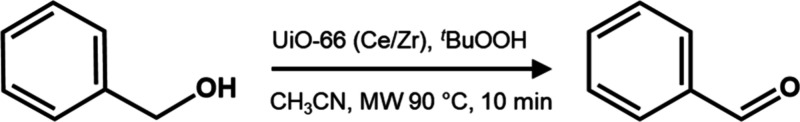
Microwave (MV)-Assisted Oxidation of Benzyl Alcohol to Benzaldehyde

The results (Table 2) indicate that the single metal Ce or Zr UiO-66 samples exhibit minimal conversion, comparable to the blank control, suggesting that the redox ability of the Ce_6_ cluster alone is similar to that of the Zr_6_ cluster. Sample BA-Ce50, consisting of 99% *cis*-Ce_2_Zr_4_ clusters, achieved a conversion of 26% after 10 min of reaction time and 12% after 5 min. In comparison, sample FA-Ce50, containing 95% CeZr_5_ clusters, yielded 10% benzaldehyde after 10 min and only 3% after 5 min. N_2_ sorption experiments (Section S8) indicate that the FA- and BA-modulated samples have similar porosity, as evidenced by comparable BET surface areas and micropore volumes (Table S16). There is no direct correlation between cerium content (wt%), determined by TGA and XRF, and catalytic performance. Despite high cerium loadings, FA-Ce75 and BA-Ce75 exhibit a lower catalytic efficiency. Consequently, rather than porosity or total Ce content, the significant difference in conversion is attributed to the cluster stoichiometries, with the *cis*-Ce_2_Zr_4_ clusters enhancing the benzaldehyde production rate compared to the CeZr_5_ clusters. Sample BA-Ce75, with a higher Ce content, exhibits lower conversion (6%) in comparison to sample BA-Ce50. This is due to the decrease in the number of *cis*-Ce_2_Zr_4_ clusters in BA-Ce75 and instead the presence of more of the Ce_6_ clusters. Given that the Ce_6_ cluster has poor reactivity, the composition of BA-Ce75, consisting of 73% Ce_6_ and 27% *cis*-Ce_2_Zr_4_ clusters, leads to lower overall redox activity. In the FA modulated samples, FA-Ce33 and FA-Ce50 have similar composition of CeZr_5_ clusters at 97% and 95% respectively and FA-Ce75 has 72% of CeZr_5_ clusters. As a result, the catalytic activity also follows a similar trend among these samples. Based on the findings, it is evident that the catalytic activity is strongly influenced by the composition of the clusters within the MOFs. Specifically, the order of catalytic activity can be observed as follows: *cis*-Ce_2_Zr_4_ > CeZr_5_ > Ce_6_ ≈ Zr_6_. The lower conversion achieved by Ce_6_ against the mixed Ce/Zr clusters can be attributed to its lower reducibility, which is supported by XPS spectra (Figure S31) that show a higher content of Ce^4+^ in the single UiO-66­(Ce) sample compared with the mixed-metal UiO-66. It is likely that the strain present in the mixed Ce/Zr clusters due to the differing Ce–O and Zr–O bond distances may play a significant role in enhancing this reducibility of catalytic performance, as mixed Ce/Zr materials, such as UiO-66­(Ce,Zr) and Ce_0.5_Zr_0.5_O_2_, exhibit superior activity compared to UiO-66­(Ce) and CeO_2_. The situation is similar to the differences between the catalytic activity of CeO_2_ compared to Ce_1–*x*_Zr_*x*_O_2_ solid solutions, where mixing the cations gives enhanced oxide-ion mobility and lower temperature redox properties, ascribed to lattice strain.^50^ Indeed, comparing the geometries of mixed structures with those of UiO-66­(Zr) and UiO-66­(Ce) reveals that SBUs which contain both Zr and Ce are highly strained due to the difference in Ce–O and Zr–O bonds, as shown in Figure 8. Notably, the most distorted bonds are the metal–oxygen bonds between a Zr–Ce octahedral edge and its connected linker, as found also by Trousselet et al.^30^ While this strain manifests most strongly around cerium for CeZr_5_ and *cis*-Ce_2_Zr_4_, it is interesting to note that a Ce_6_ SBU embedded in the zirconium material has little strain (and we expect a Ce_6_ SBU embedded in UiO-66­(Ce) to be unstrained). The trend in catalytic activity presented in Table 2 therefore follows how distorted the atomic environment around cerium atoms is in different SBUs: *cis*-Ce_2_Zr_4_ > CeZr_5_ > Ce_6_.

**Figure 8:**
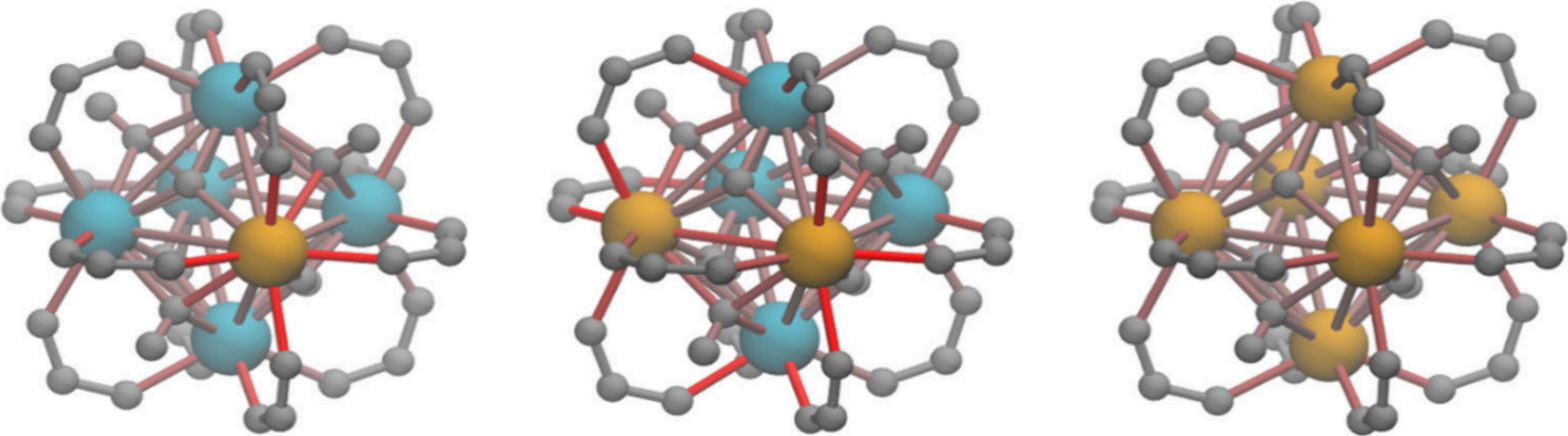
Illustration of the strain in the cluster in the periodic models of UiO-66­(Ce,Zr). Schematics show, from left to right, the cerium-containing SBU for the following configurations: 3Zr_6_–CeZr_5_, 3Zr_6_-*cis*-Ce_2_Zr_4_, and 3Zr_6_–Ce_6_. All atoms other than Zr (blue) and Ce (orange) have been colored gray. Bonds have been colored according to their strain, defined relative to the pure Zr and Ce materials, from gray (no strain) to red (∼3% strain). The overall strain increases with the number of Zr–Ce edges in the M_6_ octahedron and is most pronounced near the cerium. Tables of bond lengths and details on the calculation of strain can be found in section S11.4.

**Table 2:** Activity of UiO-66­(Ce/Zr) in the Microwave-Assisted Oxidation of Benzyl Alcohol to Benzaldehyde^a^

Entry	Catalyst	Ce wt %	Cluster Configuration	Con. (%)
1	None	n.a.	n.a.	1
2	UiO-66(Zr)	0	100% Zr_6_	3
3	UiO-66(Ce)	40	100% Ce_6_	2
4	BA-Ce33	7.5	69% *cis*-Ce_2_Zr_4_, 31% Zr_6_	13
5	BA-Ce50	11.6	99% *cis*-Ce_2_Zr_4_, 1% Ce_6_	26
6	BA-Ce50^b^	11.6	99% *cis*-Ce_2_Zr_4_, 1% Ce_6_	12
7	BA-Ce75	30.2	27% *cis*-Ce_2_Zr_4_, 73% Ce_6_	6
8	FA-Ce33	5.3	97% CeZr_5_, 3% Ce_6_	9
9	FA-Ce50	7.1	95% CeZr_5_, 5% Ce_6_	10
10	FA-Ce50^b^	7.1	95% CeZr_5_, 5% Ce_6_	3
11	FA-Ce75	14.1	72% CeZr_5_, 28% Ce_6_	6
12	CeO_2_ ^c^	81.4	n.a.	5
13	Ce_0.5_Zr_0.5_O_2_ ^c^	47.5	n.a.	11

a Configurations of the clusters in the samples are adopted from the best EXAFS fitting results. Con. = benzyl alcohol conversion. Reaction condition: 0.75 mmol benzyl alcohol, 1.1 mmol ^*t*^BuOOH, 2.5 mL acetonitrile and 100 mg catalyst (activated at 200 °C under vacuum for 2 h), heated in microwave at 90 °C for 10 min.

b 5 min reaction time.

c CeO_2_ and Ce_0.5_Zr_0.5_O_2_ reference materials are from Johnson Matthey.

Both BA-Ce50 and FA-Ce50 remained stable after the catalytic reaction, without structural collapse, as evidenced by powder XRD (Figures S35, S36). Hot filtration tests followed by ICP-OES analysis of the corresponding filtrates revealed Ce and Zr concentrations below 0.1 ppm, indicating minimal metal leaching and suggesting that the observed catalytic activity arises from the heterogeneous phase. Recyclability tests (Figure S34) on the most active sample, BA-Ce50, demonstrate that this catalyst can be reused for at least four cycles while maintaining the same performance. Previous studies have demonstrated that achieving high catalytic activity toward benzaldehyde using MOF-based catalysts often requires demanding conditions, such as precious metal Au supported by a Cu-MOF,^51^ the use of TEMPO as a cocatalyst and base (Na_2_CO_3_) for Cu_3_(BTC)_2_,^52^ or molecular oxygen (4 bar) for Pd–Cu/Sn-MOF.^53^ In contrast, our approach substantially enhances the redox activity of UiO-66 (Ce/Zr) by tuning the synthesis modulator to alter cluster configurations. Furthermore, the reaction is carried out under mild conditions using microwave irradiation and heating at 90 °C for 10 min.

Based on the reducibility of a cluster, a mechanism is proposed that involves the more active reversible switching between Ce^4+^ and Ce^3+^ within the neighboring Ce atoms in a ligand-defective *cis*-Ce_2_Zr_4_ cluster.^54,55^ The mechanism involves several key steps, as illustrated in Figure 9, each of which is based on literature observations from relevant catalytic studies on other systems. (i) Benzyl alcohol molecules adsorb onto the coordinatively unsaturated Ce atoms of the *cis*-Ce_2_Zr_4_ clusters, forming benzyl alcohol ad-species and releasing a proton.^56^ This is based on a mechanism proposed for the adsorption of benzyl alcohol at mesoporous CeO_2_. This process involves the removal of hydrogen atoms from the alcohol, which is an essential step in the selective oxidation reaction, as proposed for the gas-phase oxidation of benzyl alcohol over CeO_2_.^57^ (ii) The oxidation of the alkoxide ad-species occurs through their interaction with Ce^4+^ species situated at the redox-active sites. This interaction leads to the formation of benzaldehyde ad-species and the release of water. Simultaneously, two neighboring Ce^4+^ ions adjacent to the μ_3_-O undergo reduction to Ce^3+^ ions, as proposed to occur on CeO_2_ surfaces.^58^ We note that UiO-66­(Ce) has also been shown to have similar redox chemistry.^59^ For the mixed-metal Ce_2_Zr_4_ clusters, this is enhanced by having the pair of Ce centers adjacent in a *cis* orientation. (iii) The benzaldehyde ad-species PhCHO* desorbs from the catalyst surface, resulting in the formation of the product benzaldehyde. (iv) ^*t*^BuOOH molecules adsorb onto the missing oxygen sites of the catalyst, leading to the formation of metal-oxo species. This process also reoxidizes the Ce^3+^ ions back to Ce^4+^, based on catalysis over CeO_2_.^60,61^ (v) The metal-oxo species then transfers one oxygen atom to bridge the Ce^4+^ ions, regenerating the *cis*-Ce_2_Zr_4_ clusters. This step allows for the Ce^4+^ species to be restored, at the same time, *tert*-butyl alcohol is released.

**Figure 9:**
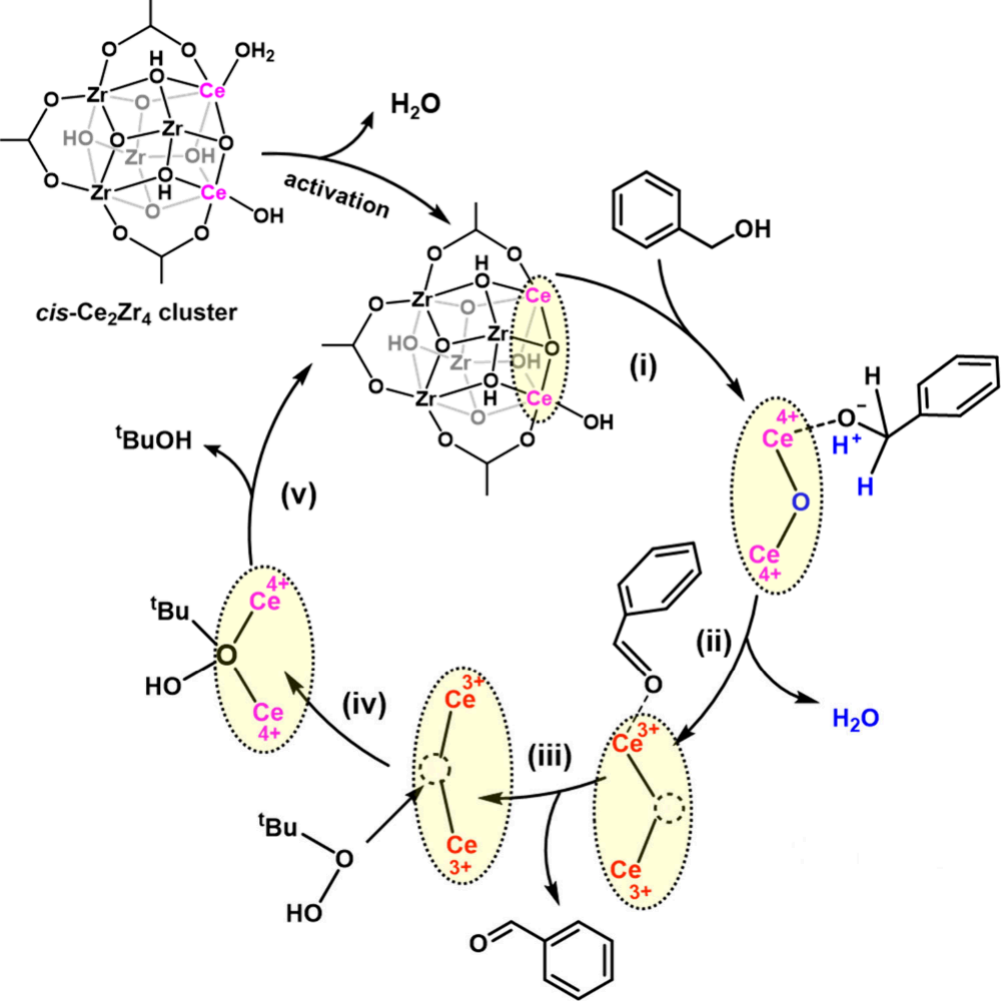
Illustration of the proposed mechanism for the oxidation of benzyl alcohol to benzaldehyde over a ligand-defective *cis*-Ce_2_Zr_4_ cluster with the presence of ^*t*^BuOOH as oxidant.

Recent work on MOFs in catalysis have discovered that binding sites at metal centers can be generated *in situ* via hemilabile ligands, and this may be a possible mechanism for the binding of benzyl alcohol in Step (i) in Figure 9. However, the literature examples of this effect use weakly binding ligands to achieve this,^62,63^ or metals of lower charge,^64,65^ and small displacing molecules such as SO_2_ or methanol, so it is likely that for our materials simply the presence of linker defects provides the binding sites for benzyl alcohol, where only water needs to be displaced.

The difference in the redox activity between the BA and FA-modulated samples aligns with the preferential formation of *cis*-Ce_2_Zr_4_ and CeZr_5_ cornerstones. This is in agreement with DFT calculations; the energy cost of removing a bridging oxygen atom in a two-electron reduction for an *M*_*6*_ cluster is less than half as large for *cis*-Ce_2_Zr_4_ as it is for CeZr_5_ (∼ 3.1 vs 6.7 eV). In the first case, the additional electron density localizes on both cerium atoms to reduce them to the 3+ state. In the latter, half of the electron density localizes on cerium, whereas the rest delocalizes over multiple zirconium atoms, a less stable configuration given its unfavorable 3+ oxidation state. This highlights the importance of controlling cation distribution through various synthesis conditions to tune the redox property of UiO-66­(Ce/Zr).

## Conclusions

In conclusion, Ce and Zr K-edges EXAFS, Ce L_III_-edge XANES, *in situ* heating FTIR spectroscopy, along with DFT calculations, reveal that using benzoic acid as a modulator leads to the preferable formation of the [(*cis*-Ce_2_Zr_4_)­(OH)_4_O_4_]^12+^ cluster in UiO-66­(Ce/Zr) with a higher Ce incorporation than formic acid modulated synthesis that results in a [(CeZr_5_)­(OH)_4_O_4_]^12+^ cluster composition. Analysis of the Ce K-edge EXAFS and L_III_-edge XANES results reveals that the formation of isolated Ce­(III) in solution limits the uptake of Ce into the UiO-66 structure. The local metal distribution of Ce and Zr in the *M*_*6*_ clusters significantly impacts the catalytic activity in the benzyl alcohol oxidation reaction. The results indicate a distinct order of catalytic activity, with *cis*-Ce_2_Zr_4_ exhibiting the highest activity, followed by CeZr_5_, and then Ce_6_ and Zr_6_ showing comparable activity. The local strain of the *cis*-Ce_2_Zr_4_ is likely the origin of the catalytic activity, akin to the solid-state redox properties of ceria-zirconia oxygen storage materials. The modulated control of cation uptake, leading to specific distributions with metal clusters, may be applicable to other cation substitutions in UiO-66 to lead to fine-tuning of its catalysis properties, and potentially to other MOF materials.

## Experimental Section

### Materials

Ammonium cerium nitrate (98.5%, (NH_4_)_2_Ce­(NO_3_)_6_, Merck), zirconium oxynitrate hydrate (99%, ZrO­(NO_3_)_2_·H_2_O, Sigma-Aldrich), benzene-1,4-dicarboxylic acid (98%, H_2_BDC, Sigma-Aldrich), formic acid (96%, HCOOH, Merck), acetic acid (99.7%, CH_3_COOH, Merck) benzoic acid (99.9%, Alfa Aesar), *N,N*-dimethylformamide (99.8%, DMF, Fisher Chemical), *N,N*-dimethylacetamide (99.8%, DMA, Sigma-Aldrich), and *N,N*-diethylformamide (99%, DEF, Sigma-Aldrich) were used in the synthesis as provided without further treatments.

Benzyl alcohol (anhydrous, 99.8%, Sigma-Aldrich), *tert*-butyl hydroperoxide solution (5.0–6.0 M in decane, ^*t*^BuOOH, Sigma-Aldrich), acetonitrile (99.8%, CH_3_CN, Fisher Chemical), 1,4-dioxane (99.8%, for analysis, Fisher Chemical) and deuterochloroform (99%, CDCl_3_, Sigma-Aldrich) were used as purchased in the catalysis reaction and further analysis.

### Synthesis

The synthesis of mixed-metal UiO-66­(Ce, Zr) using different modulators is based on a previous study.^26^ In a typical synthesis, benzene-1,4-dicarboxylic acid (63.8 mg, 0.76 mmol), DMF (1.8 mL), aqueous solutions of (NH_4_)_2_Ce­(NO_3_)_6_ (0.53 M) and ZrO­(NO_3_)_2_·H_2_O (0.53 M) and monocarboxylic acid modulators were added to a 7 mL glass reaction tube and sealed. The exact amount of the added reagents is shown in Tables S1–S3, ESI. After ultrasonication for 5 min, the sealed glass tube was placed in a preheated oil bath under continuous stirring and heated at 100 °C for 15 min. The solid was separated through centrifugation, redisperse and sonicate twice in DMF (5 mL) to remove the unreacted ligand, then three times with acetone (20 mL) to exchange DMF, and dried at 70 °C overnight. Various mixed-metal UiO-66­(Ce, Zr) compounds with intended Ce:Zr metal ratios from 1:11 to 11:1 were prepared.

### Characterization

Powder XRD patterns were collected on a Malvern Panalytical Empyrean diffractometer with Cu Kα radiation (λ = 1.5418 Å) using multicore optics and a Pixcel3D detector. Data were recorded from 5° to 50° 2θ (0.04° step size, 2s/step). Unit cell parameters were determined through Pawley fitting using GSAS-II software.^66^ The XRF measurement was performed using a Rigaku Primus IV wavelength dispersive XRF spectrometer with an X-ray tube operating power up to 4 kW. The TGA analysis was performed using a Mettler Toledo TGA/DSC 1 instrument where samples were heated in an air atmosphere from 25 to 1000 °C with a heating rate of 10 °C/min. Morphology and elemental analysis were performed using a JEOL 2100 TEM (LaB_6_, 200 kV) and JEOL ARM200F TEM/STEM (Schottky gun, 80 kV). ADF-STEM imaging was conducted using CEOS correctors, with a 23 pA probe current, 25 mrad convergence semiangle, and 45–50 mrad inner angle. STEM-EDX mapping used an Oxford Instruments X-MaxN 100TLE SSD detector. XAS experiments at the Ce L_III_-edge, Ce K-edge and Zr K-edge were performed at BM28 ^67^ beamline (XMaS) of European Synchrotron Radiation Facility (ESRF). Ce K-edge and Zr K-edge were performed in transmission mode, and the Ce L_III_-edge was studied in fluorescence mode. Solid samples were diluted with appropriate amounts of cellulose powder and pressed into self-supporting pellets. Data were merged and normalized using Athena, with subsequent EXAFS fitting performed in Artemis (Demeter package).^68^ Detailed experimental parameters are provided in ESI, Section S6. FTIR data were collected by using a Shimadzu IRTracer-100 instrument spectrometer at 2 cm^–1^ resolution. The LabSolutions IR software was employed to monitor the spectra in real-time and analyze the obtained results. The detailed *in situ* setup is described in Supporting Information, Section S7. Surface area and porosity were determined by using N_2_ gas sorption on a Micromeritics ASAP 2020 instrument. Samples were degassed under vacuum at 120 °C for 4 h prior to measurement. XPS analysis was performed using a Kratos Axis Ultra DLD spectrometer with monochromated Al Kα radiation (1.487 keV) under ultrahigh vacuum (<1 × 10^–9^ mbar). Spectra were calibrated to the C–C/C-H peak in the C 1s region (285.0 eV) and analyzed using Casa XPS software, with appropriate corrections for sensitivity factors and photoelectron parameters.

### Catalytic Experiment

The MOF catalysts were activated by vacuum degassing at 200 °C for 2 h, except for UiO-66­(Ce) which was activated at 180 °C due to lower thermal stability. The activated catalysts were used immediately after cooling to room temperature.

In a typical oxidation reaction, benzyl alcohol (81 mg, 0.75 mmol) and activated catalyst (100 mg) were combined with acetonitrile (2.5 mL) in a glass microwave reaction tube. ^*t*^BuOOH solution (0.2 mL, 5.0–6.0 M in decane) was added, and the sealed tube was sonicated for 10 min. The reaction was heated to 90 °C in an Anton-Parr Monowave 200 microwave reactor with magnetic stirring for 10 min, followed by rapid cooling to room temperature.

For product analysis, the cooled reaction mixture was combined with 1,4-dioxane (∼100 mg) as an internal standard. The mixture was then centrifuged to separate the solution from the solid catalyst. The filtered solution was diluted with CDCl_3_ for the ^1^H NMR analysis. ^1^H NMR measurements were done with a Bruker Avance III HD 300 MHz instrument. Quantitative analysis was performed on Bruker TopSpin NMR Analysis software.

### Computational Method

The calculations were carried out at the DFT level using CP2K. The PBE functional was used together with Grimme’s D3 and three body semiempirical corrections. Double-zeta MOLOPT Gaussian primary basis sets and standard Goedecker-Tetter-Hutter (GTH) pseudopotentials were used for all atoms except cerium; a plane wave kinetic energy cutoff of 1200 Ry was used for the secondary basis set. For cerium, the basis set and pseudopotential of Lu et al. were used instead.^69^ A Hubbard correction was applied to cerium’s 4f shell with a single effective parameter *U*_*eff*_ of 7 eV. For systems containing Ce­(III), the PBE0 functional was instead used along with triple-zeta MOLOPT basis sets. The Hartree–Fock contribution was determined with the auxiliary density matrix method (ADMM) for which Fit3 and Fit11 auxiliary basis sets were used for all atoms other than Ce. An auxiliary basis set developed for CeO_2_ by Hahn et al. was used for cerium.^70^ Other parameters remained unchanged.

During self-consistent field (SCF) cycles and structural optimizations, the following convergence criteria were enforced: 10^–8^, 10^–3^, 10^–4^, 4.5 × 10^–4^, and 4.5 × 10^–4^ (in atomic units) respectively for the energy, maximum step size, root-mean-square step size, maximum force, and root-mean-square force. The energy criteria was relaxed to 10^–6^ as needed for some calculations involving Ce­(III) in which the SCF had difficulty converging. Aperiodic clusters and molecules were simulated inside an empty 25 × 25 × 25 Å^3^ box to minimize interactions between periodic images. All *k*-point sampling was done at the gamma point, given the large sizes of the unit cells involved.

## Supplementary Material



## Data Availability

The data underlying this study are openly available in the WRAP and ESRF repositories at the following urls: https://wrap.warwick.ac.uk/195015/, https://doi.org/10.15151/ESRF-DC-2303883875

## Notes

PXRD, TGA, TEM, FTIR, Ce L_III_-edge XANES, Ce/Zr K-edge EXAFS fittings, XPS, N_2_ sorption, catalytic studies, ^1^H NMR spectra and DFT calculation results (PDF)
