# Supplementary material for: Modulator Control of Cation Distribution in Mixed-Metal UiO-66(Zr,Ce) Metal–Organic Frameworks
Source: Chem Mater. 2025 Dec 10;37(24):9676–88. doi: 10.1021/acs.chemmater.5c01629 (PMC12746409; doi:10.1021/acs.chemmater.5c01629)
Supplement: Supplementary file 1 [file cm5c01629_si_001.pdf]

Supporting Information

Modulator Control of Cation Distribution in Mixed-Metal UiO-66(Zr,Ce) Metal-Organic Frameworks

Baiwen Zhao,[1] Martin Hutereau,[2] Marc Walker,[3] Reza J. Kashtiban,[3] Paul B.J. Thompson,[4] Ben Slater[2] and Richard I. Walton[1]*

1. Department of Chemistry, University of Warwick, Gibbet Hill Road, Coventry, CV4 7AL, U.K. Author for correspondence: r.i.walton@warwick.ac.uk
2. Department of Chemistry, University College London, 20 Gordon Street, London, WC1E 6BT, U.K.
3. Department of Physics, University of Warwick, Gibbet Hill Road, Coventry, CV4 7AL, U.K.
4. XMaS Beamline, European Synchrotron Radiation Facility, Grenoble F-38043, France

Table of Contents

S1. Materials 1

S2. Synthesis 1

S3. Structural and Compositional Studies 4

S4. Thermal Analysis 13

S5. TEM 19

S6. XAFS 20

S7. FTIR Spectroscopy 31

S8. N_2 Sorption Experiments 33

S9. XPS 35

S10. Catalysis Studies 37

S11. Density Functional Theory 44

References 54

S1. Materials

Ammonium cerium nitrate (98.5%, $(NH_4)_2Ce(NO_3)_6$, Merck), zirconium oxynitrate hydrate (99%, $ZrO(NO_3)_2 \cdot H_2O$, Sigma-Aldrich), benzene-1,4-dicarboxylic acid (98%, H_2BDC, Sigma-Aldrich), formic acid (96%, HCOOH, Merck), acetic acid (99.7%, CH_3COOH, Merck) benzoic acid (99.9%, Alfa Aesar), *N,N*-dimethylformamide (99.8%, DMF, Fisher Chemical), *N,N*-dimethylacetamide (99.8%, DMA, Sigma-Aldrich), and *N,N*-diethylformamide (99%, DEF, Sigma-Aldrich) were used in the synthesis as provided without further treatments.

Benzyl alcohol (anhydrous, 99.8%, Sigma-Aldrich), *tert*-butyl hydroperoxide solution (5.0-6.0 M in decane, ^{t}BuOOH, Sigma-Aldrich), acetonitrile (99.8%, CH_3CN, Fisher Chemical), 1,4-dioxane (99.8%, for analysis, Fisher Chemical) and Deuterochloroform (99%, $CDCl_3$, Sigma-Aldrich) were used as purchased in the catalysis reaction and further analysis.

S2. Synthesis

The synthesis of mixed-metal UiO-66(Ce,Zr) using formic acid as modulator is based on a previous study.[1] In a typical synthesis, benzene-1,4-dicarboxylic acid (63.8 mg, 0.76 mmol) DMF (1.8 mL), aqueous solutions of $(NH_4)_2Ce(NO_3)_6$ (0.53 M) and $ZrO(NO_3)_2 \cdot H_2O$ (0.53 M) and concentrated formic acid (0.5 mL, 13.3 mmol) were added to a 7 mL glass reaction tube and sealed. The exact amount of the added reagents is shown in Table S1. After ultrasonication for 5 minutes, the sealed glass tube was placed in a pre-heated oil bath under continuous stirring and heated at 100 °C for 15 minutes. The solid was separated through centrifugation, re-disperse and sonicate twice in DMF (5 mL) to remove the unreacted ligand, then three times with acetone (20 mL) to exchange DMF, and dried at 70 °C overnight. Eleven mixed-metal UiO-66(Ce,Zr) compounds with intended Ce:Zr metal ratios from 1:11 to 11:1 were prepared.

Single-metal UiO-66(Ce) and UiO-66(Zr) were also synthesised for reference (Table S1). UiO-66(Ce) was synthesised by the same method as above described without adding any modulator. For the synthesis of UiO-66(Zr), 0.5 mL of formic acid was added, and the reaction time was extended to 1 hour. Both samples of single metal (Ce, Zr) UiO-66 were washed with DMF then three times with acetone, and then dried at 70 °C in air.

Table S1 Synthesis of mixed-metal UiO-66(Ce/Zr) modulated by formic acid, and single-metal UiO-66(Ce) and UiO-66(Zr).

Sample	Ratio		Ce (mL)	Zr (mL)	H_2BDC (mg)	HCOOH (mL)	DMF (mL)
	Ce	Zr					
FA-Ce8	1	11	0.05	0.55	63.8	0.5	1.8
FA-Ce17	2	10	0.10	0.50	63.8	0.5	1.8
FA-Ce25	3	9	0.15	0.45	63.8	0.5	1.8
FA-Ce33	4	8	0.20	0.40	63.8	0.5	1.8
FA-Ce42	5	7	0.25	0.35	63.8	0.5	1.8
FA-Ce50	6	6	0.30	0.30	63.8	0.5	1.8
FA-Ce58	7	5	0.35	0.25	63.8	0.5	1.8
FA-Ce67	8	4	0.40	0.20	63.8	0.5	1.8
FA-Ce75	9	3	0.45	0.15	63.8	0.5	1.8
FA-Ce83	10	2	0.50	0.10	63.8	0.5	1.8
FA-Ce92	11	1	0.55	0.05	63.8	0.5	1.8
UiO-66(Zr)	0	1	0	0.6	63.8	0.5	1.8
UiO-66(Ce)	1	0	0.6	0	63.8	0	1.8

The synthesis of mixed-metal UiO-66(Ce,Zr) using benzoic acid as a modulator follows a procedure similar to that using formic acid as a modulator. Benzene-1,4-dicarboxylic acid (63.8 mg, 0.76 mmol) DMF (1.8 mL), aqueous solutions of $(NH_4)_2Ce(NO_3)_6$ (0.53 M) and $ZrO(NO_3)_2{\cdot}H_2O$ (0.53 M) and benzoic acid (810 mg, 6.7 mmol) were added to a 7 mL glass reaction tube and sealed. The exact amount of the added reagents is shown in Table S2. After ultrasonication for 5 minutes, the sealed glass tube was placed in a pre-heated oil bath under continuous stirring and heated at 100 °C for 20 minutes. The solid was separated through centrifugation, re-disperse and sonicate twice in DMF (5 mL) to remove the unreacted ligand, then three times with acetone (20 mL) to exchange DMF, and dried at 70 °C overnight. Eleven mixed-metal UiO-66(Ce,Zr) compounds with intended Ce:Zr metal ratios from 1:11 to 11:1 were prepared.

Table S2 Synthesis of mixed-metal UiO-66(Ce/Zr) modulated by benzoic acid.

Sample	Ratio		Ce	Zr	H_2BDC	Benzoic acid	DMF
	Ce	Zr	(mL)	(mL)	(mg)	(mg)	(mL)
BA-Ce8	1	11	0.05	0.55	63.8	810	1.8
BA-Ce17	2	10	0.10	0.50	63.8	810	1.8
BA-Ce25	3	9	0.15	0.45	63.8	810	1.8
BA-Ce33	4	8	0.20	0.40	63.8	810	1.8
BA-Ce42	5	7	0.25	0.35	63.8	810	1.8
BA-Ce50	6	6	0.30	0.30	63.8	810	1.8
BA-Ce58	7	5	0.35	0.25	63.8	810	1.8
BA-Ce67	8	4	0.40	0.20	63.8	810	1.8
BA-Ce75	9	3	0.45	0.15	63.8	810	1.8
BA-Ce83	10	2	0.50	0.10	63.8	810	1.8
BA-Ce92	11	1	0.55	0.05	63.8	810	1.8

For the synthesis using different modulators and solvents, the procedure is very similar to the ones described above. Benzene-1,4-dicarboxylic acid (63.8 mg, 0.76 mmol), 0.6 mL of aqueous solutions of $(NH_4)_2Ce(NO_3)_6$ (0.53 M) 0.6 mL of $ZrO(NO_3)_2{\cdot}H_2O$ (0.53 M), different solvent (DEF, DMF and DMA), and different modulators (benzoic acid, acetic acid and formic acid) were added to a 7 mL glass reaction tube and sealed. Detailed synthesis parameters are shown in Table S3. After ultrasonication for 5 minutes, the sealed glass tube was placed in a pre-heated oil bath under continuous stirring and heated at 100 °C for 20 minutes. The solid was separated through centrifugation, re-disperse and sonicate twice in DMF (5 mL) to remove the unreacted ligand, then three times with acetone (20 mL) to exchange DMF, and dried at 70 °C overnight. Mixed-metal UiO-66(Ce,Zr) compounds with intended Ce:Zr metal ratios of 1:1 were prepared.

Table S3 Synthesis of mixed-metal UiO-66(Ce/Zr) of 50/50 initial Ce/Zr ratio with different modulators and solvent.

Sample	Ce (mL)	Zr (mL)	H_2BDC (mg)	Modulator	Solvent (mL)
BA-Ce50 (DEF)	0.30	0.30	63.8	BA 810 mg	DEF 1.8 mL
FA-Ce50 (DEF)	0.30	0.30	63.8	FA 0.50 mL	DEF 1.8 mL
AA-Ce50 (DMF)	0.30	0.30	63.8	AA 0.76 mL	DMF 1.8 mL
AA-Ce50 (DMA)	0.30	0.30	63.8	AA 0.76 mL	DMA 1.8 mL

S3. Structural and Compositional Studies

Powder XRD measurements were conducted using a 3rd generation Malvern Panalytical Empyrean instrument equipped with multicore (iCore/ dCore) optics and a Pixcel3D detector. A Cu tube was used, providing Cu $K\alpha_{1/2}$ radiation (λ = 1.5418 Å). Diffraction data were recorded between 5° and 50° 2θ, with a step size of 0.04° and a counting time of approximately 2 seconds per step. The GSAS-II software was used to perform Pawley fits of the powder XRD patterns to determine unit cell parameters.[2]

The Ce/Zr ratio of the samples was determined by X-ray fluorescence (XRF) spectroscopy. The XRF measurement was performed using a Rigaku Primus IV wavelength dispersive XRF spectrometer with an X-ray tube operating power up to 4 kW. In order to ensure accurate quantitative analysis, each sample was scanned for 20 minutes to acquire a sufficient number of counts. The obtained results are summarised in Table S4, comparing with the Ce content derived from Pawley fit of powder XRD.

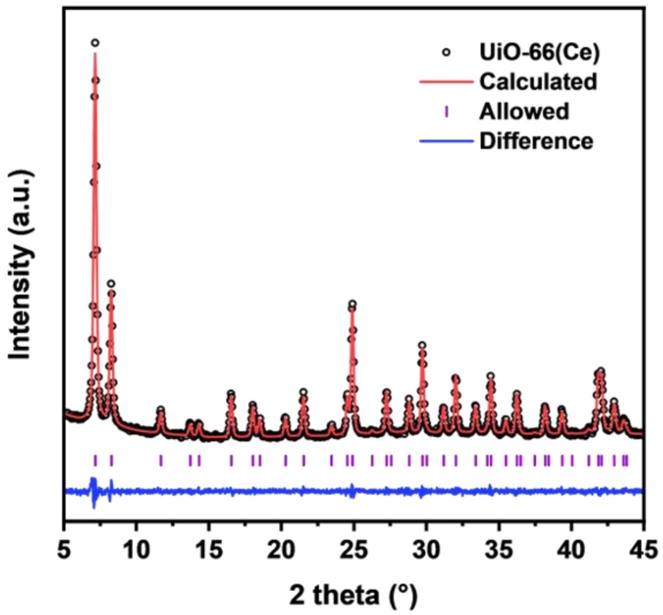

Figure S1 Powder XRD patterns of the synthesised UiO-66(Ce) refined using a Pawley fit (Space group $Fm\bar{3}m$).

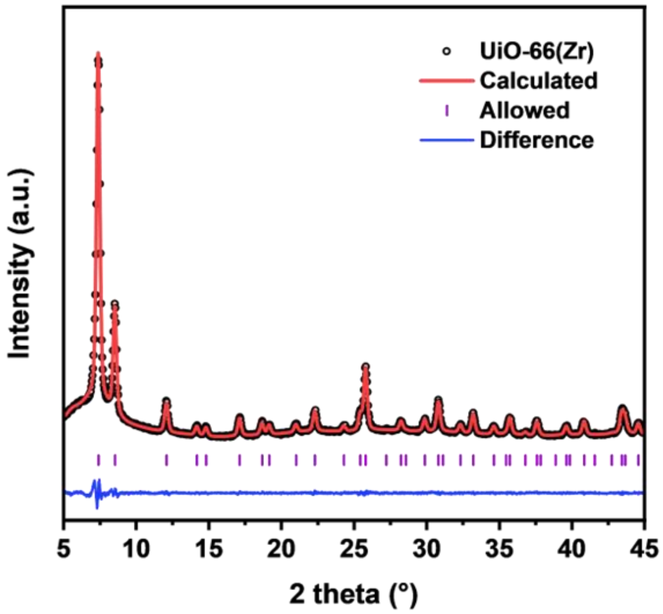

Figure S2 Powder XRD patterns of the synthesised UiO-66(Zr) refined using a Pawley fit (Space group $Fm\bar{3}m$).

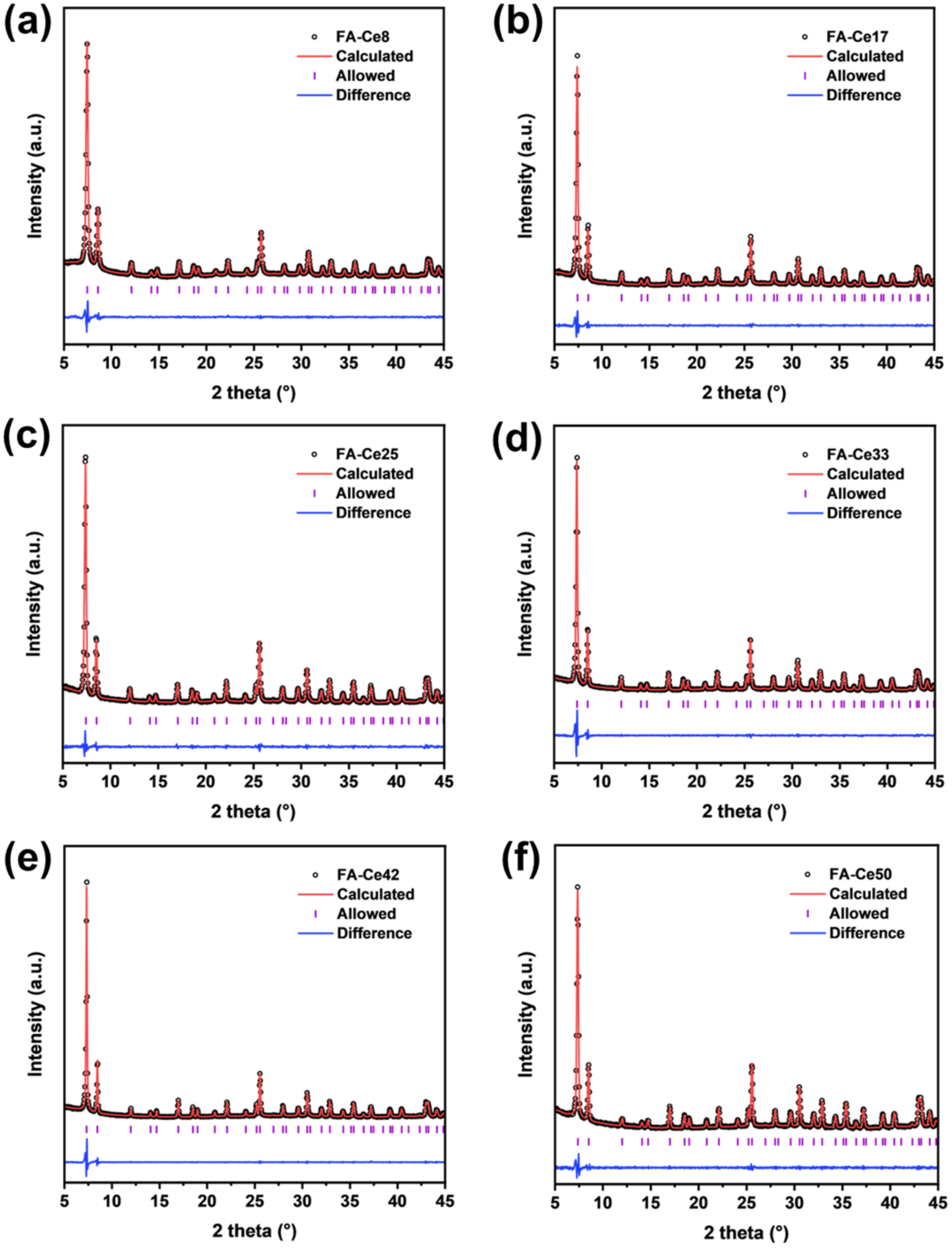

Figure S3 Powder XRD patterns refined using Pawley fit (Space group $Fm\bar{3}m$) of (a) FA-Ce8, (b) FA-Ce17, (c) FA-Ce25, (d) FA-Ce33, (e) FA-Ce42, and (f) FA-Ce50

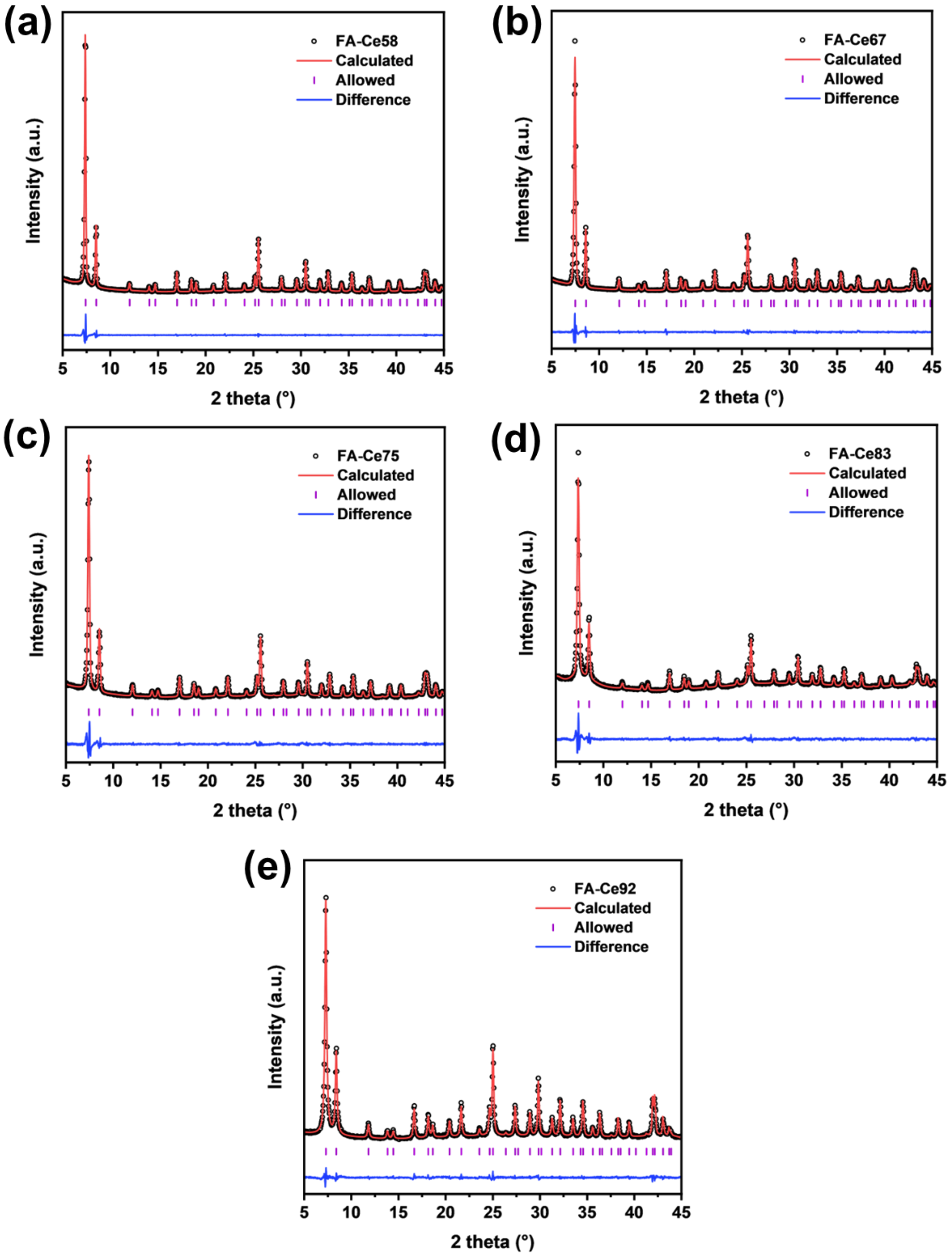

Figure S4 Powder XRD patterns refined using Pawley fit (Space group $Fm\bar{3}m$) of (a) FA-Ce58, (b) FA-Ce67, (c) FA-Ce75, (d) FA-Ce83, and (e) FA-Ce92

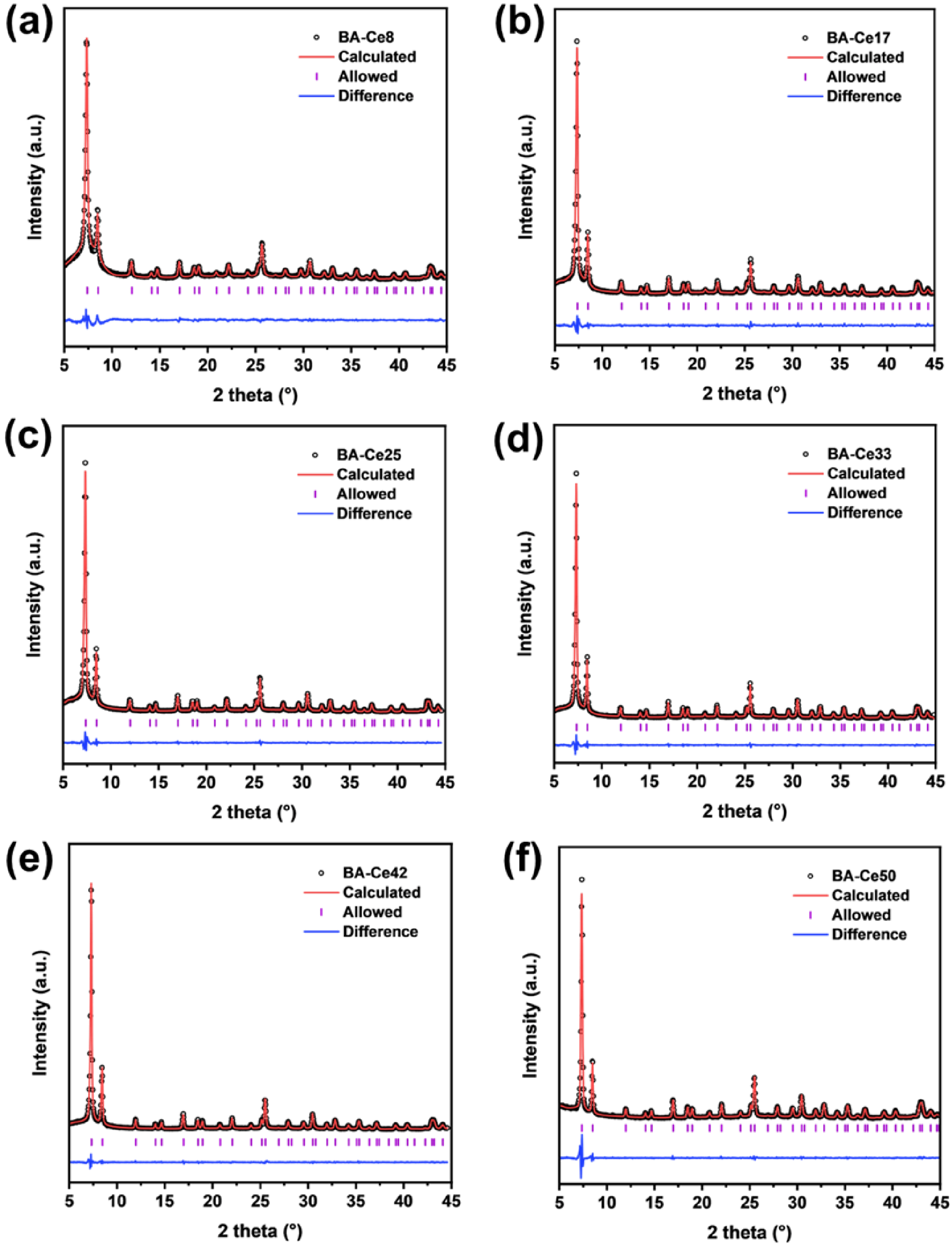

Figure S5 Powder XRD patterns refined using Pawley fit (Space group $Fm\bar{3}m$) of (a) BA-Ce8, (b) BA-Ce17, (c) BA-Ce25, (d) BA-Ce33, (e) BA-Ce42, and (f) BA-Ce50

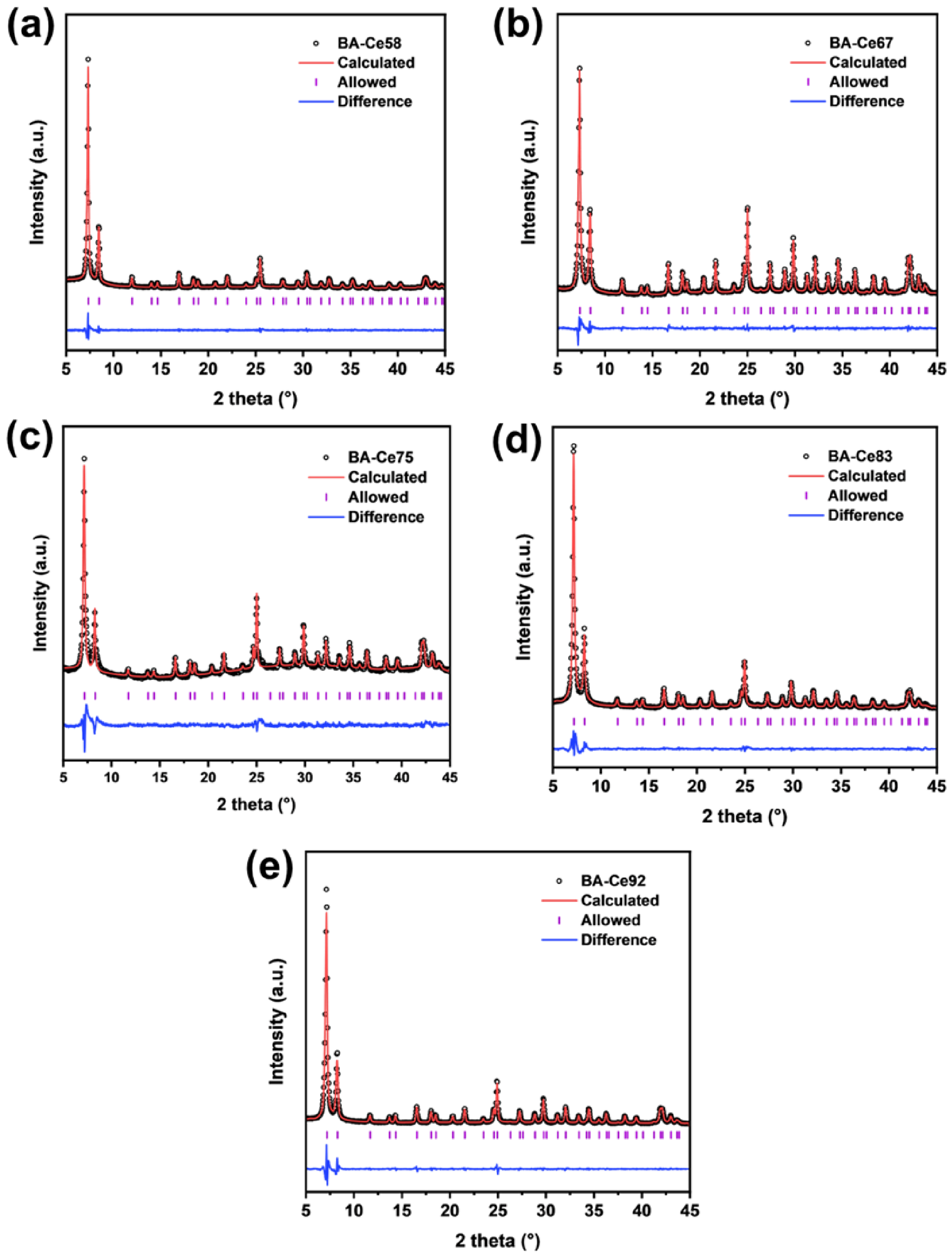

Figure S6 Powder XRD patterns refined using Pawley fit (Space group $Fm\bar{3}m$) of (a) BA-Ce58, (b) BA-Ce67, (c) BA-Ce75, (d) BA-Ce83, and (e) BA-Ce92

The results of the Pawley refinement and the derived Ce% content by lattice parameter, along with the Ce% obtained by XRF, are presented in Table S4.

The Ce content was calculated as follows (Vegard's law):

$$Ce\%_{Calculated} = (a_{Ce/Zr} - a_{Zr})/(a_{Ce} - a_{Zr}) \qquad \text{(Equation S1)}$$

where $a_{Ce/Zr}$ is the lattice parameter of the mixed-metal UiO-66(Ce/Zr) from Pawley fit, and a_{Ce} and a_{Zr} represent the lattice parameter of the synthesised UiO-66(Ce) and UiO-66(Zr) respectively.

The Ce content obtained from both the lattice parameter analysis using Pawley refinement and XRF measurements exhibit strong agreement. The close agreement between the two methods suggests that the lattice parameter analysis and XRF measurements provide consistent determinations of the Ce content in the MOF samples.

Table S4 Results of the Pawley refinement and the derived Ce% content by lattice parameter, along with the Ce% content obtained by XRF for the synthesised UiO-66 compounds.

Sample	Intended Ce/ %	a / Å	R_{wp}/ %	GoF	Ce/ % by Pawley	Ce/ % by XRF
UiO-66(Zr)	0	20.7473(5)	4.5	1.4	0	0
UiO-66(Ce)	100	21.4795(6)	3.5	1.2	100	100
FA-Ce8	8.3	20.7971(5)	4.3	1.5	6.8	5.8
FA-Ce17	16.7	20.8489(4)	5.5	1.6	13.9	12.7
FA-Ce25	25.0	20.8713(3)	4.9	1.4	16.9	15.0
FA-Ce33	33.3	20.8836(6)	6.4	1.7	18.6	19.3
FA-Ce42	41.7	20.9008(4)	7.3	2.1	21.0	19.1
FA-Ce50	50.0	20.9187(5)	5.0	1.3	23.4	20.8
FA-Ce58	58.3	20.9514(5)	5.5	1.7	27.9	25.4
FA-Ce67	66.7	20.9831(6)	6.5	1.9	32.2	33.3
FA-Ce75	75.0	20.9911(6)	6.9	1.9	33.3	41.4
FA-Ce83	83.3	21.0204(9)	5.2	2.0	37.3	54.2
FA-Ce92	91.7	21.3354(8)	5.9	1.9	80.3	75.5
BA-Ce8	8.3	20.7953(11)	6.1	2.0	6.5	8.2
BA-Ce17	16.7	20.8285(5)	5.5	1.8	11.1	13.8
BA-Ce25	25.0	20.8586(4)	4.7	1.6	15.2	16.8
BA-Ce33	33.3	20.8985(4)	4.8	1.5	20.6	22.7
BA-Ce42	41.7	20.9373(4)	4.4	1.4	25.9	25.5
BA-Ce50	50.0	20.9897(5)	4.5	1.3	31.9	33.7
BA-Ce58	58.3	20.9932(5)	5.4	1.5	33.5	35.2
BA-Ce67	66.7	21.1974(4)	5.5	1.4	61.4	65.8
BA-Ce75	75.0	21.3354(6)	6.5	2.4	80.2	82.1
BA-Ce83	83.3	21.3940(7)	6.9	1.8	88.2	89.6
BA-Ce92	91.7	21.4442(5)	7.3	2.0	95.1	93.3

Table S5 Fitted unit cell lattice parameters and calculated Ce contents of samples synthesised by different combinations of solvent and modulators with 50% intended Ce contents. Sample AA-Ce50 (DMA), AA-Ce50 (DEF) and BA-Ce50 (DEF) cannot be successfully synthesised.

Sample	Solvent	Modulator	Lattice parameter *a* / Å	Calculated Ce /%
FA-Ce50	DMF	Formic acid	20.9187	22.6
AA-Ce50 (DMF)	DMF	Acetic acid	20.9268	24.6
BA-Ce50	DMF	Benzoic acid	20.9970	34.2
FA-Ce50 (DMA)	DMA	Formic acid	20.9724	30.8
AA-Ce50 (DMA)	DMA	Acetic acid	-	-
BA-Ce50 (DMA)	DMA	Benzoic acid	20.9332	25.5
FA-Ce50 (DEF)	DEF	Formic acid	20.8923	19.8
AA-Ce50 (DEF)	DEF	Acetic acid	-	-
BA-Ce50 (DEF)	DEF	Benzoic acid	-	-

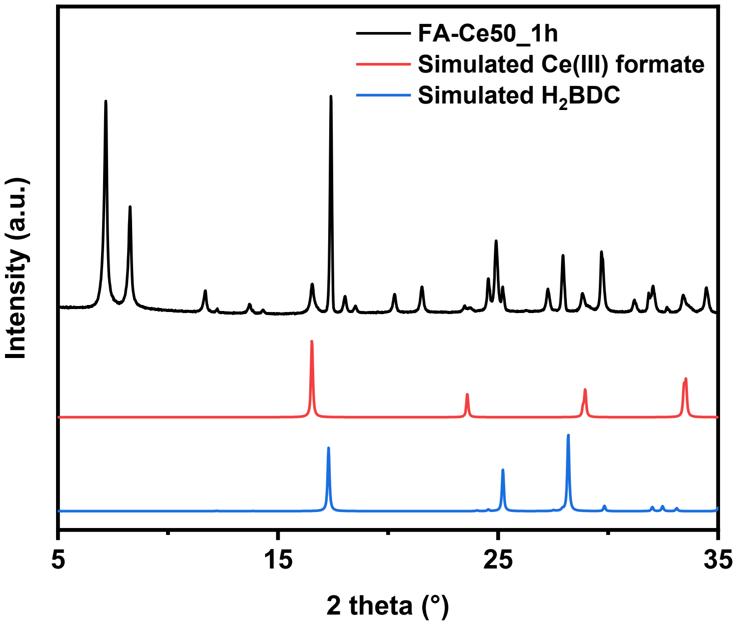

Figure S7 Powder XRD patterns of unwashed FA-Ce50 sample reacted for 1h in comparison with the simulated patterns of Ce(III) formate[3] and ligand terephthalic acid.[4]

S4. Thermal Analysis

The TGA analysis was performed using a Mettler Toledo TGA/DSC 1 instrument where samples were heated in air atmosphere from 25 to 1000 °C with a heating rate of 10 °C/min.

The chemical composition and mass percentage for the samples at each step are provided in Figure S8, S9. By comparing the Δm from the decomposition of BDC and modulator with the calculated values for a perfect structure, the missing ligand defects can be determined.

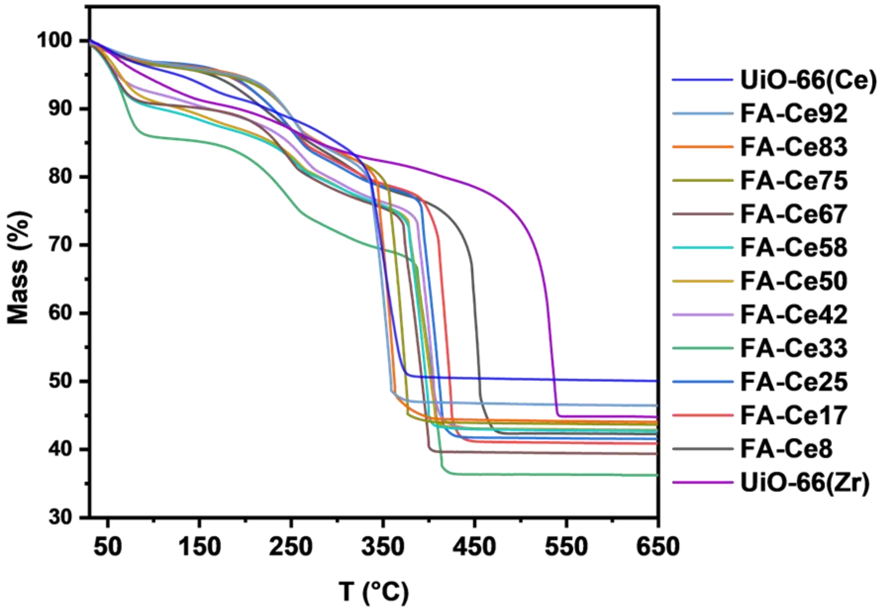

Figure S8 TG curves measured in air showing thermal stability of the as made series of formic acid modulated UiO-66(Ce/Zr) samples.

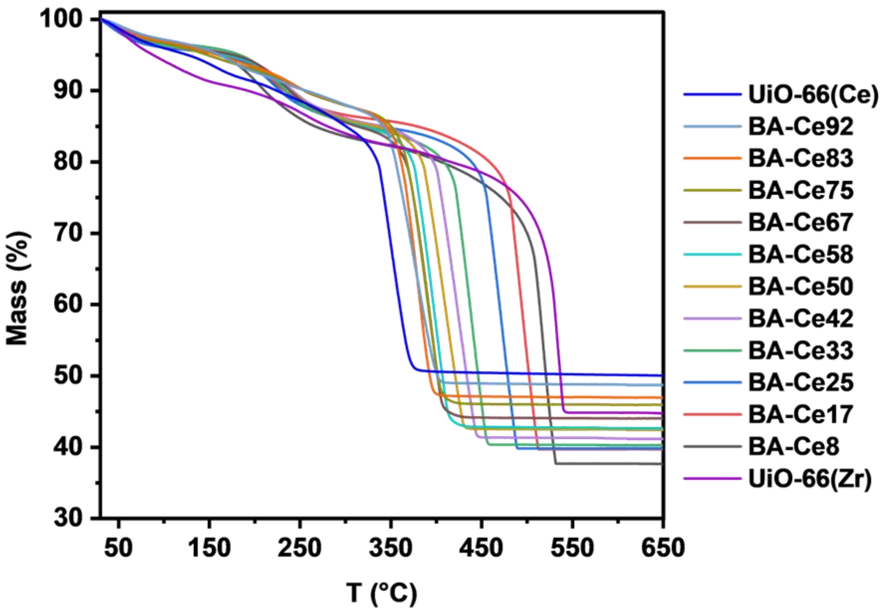

Figure S9 TG curves measured in air showing thermal stability of the as made series of benzoic acid modulated UiO-66(Ce/Zr) samples.

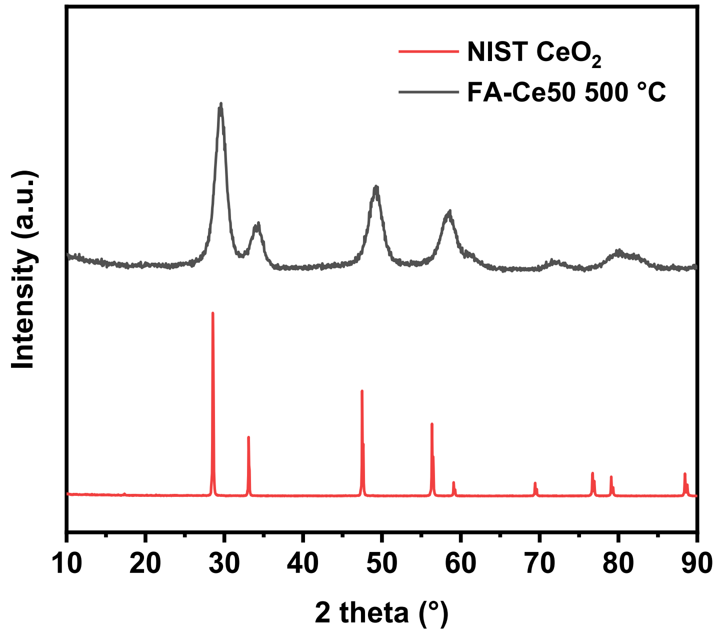

Figure S10 Powder XRD patterns of residue phase of sample FA-Ce50 after heating in air at 500 °C, shown in comparison with the reference patterns of NIST CeO_2. Note the smaller unit cell of the measured pattern, consistent with Zr inclusion.

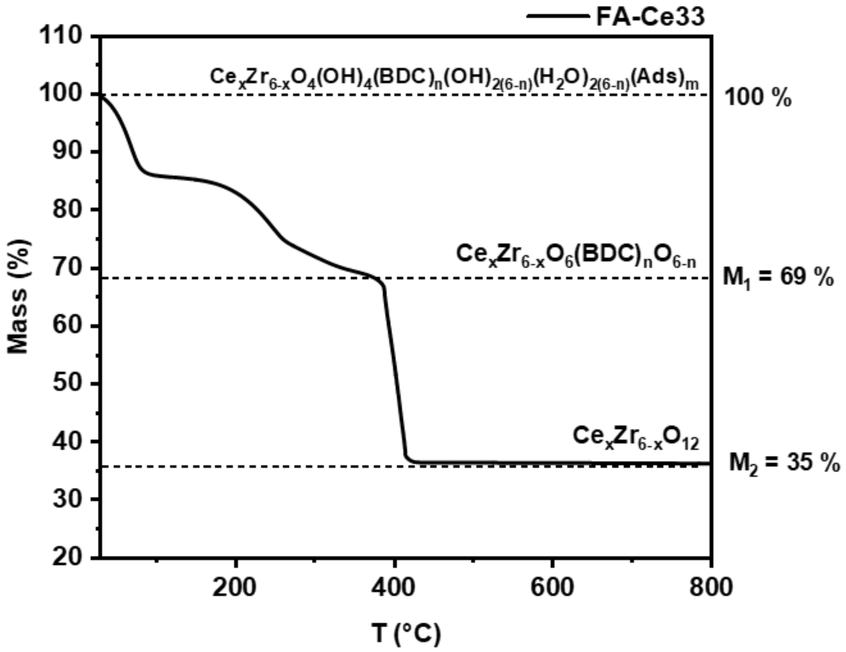

Figure S11 TGA trace of FA-Ce33 with chemical composition described and mass percentage at each stage of decomposition, Ads = adsorbed H_2O, DMF etc.

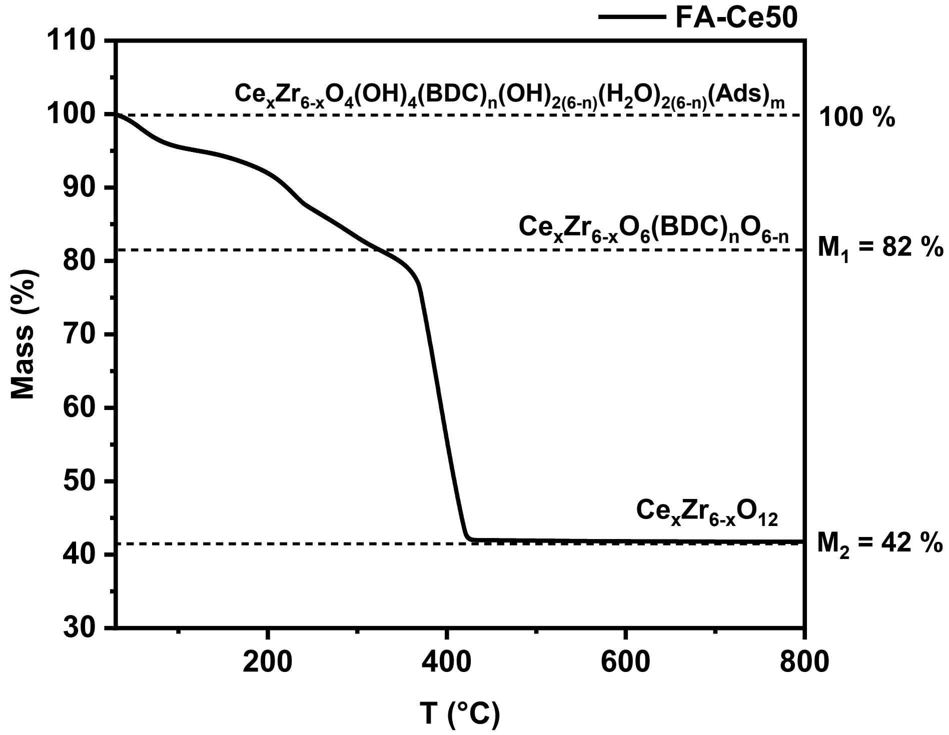

Figure S12 TGA trace of FA-Ce50 with chemical composition described and mass percentage at each stage of decomposition, Ads = adsorbed H_2O, DMF etc.

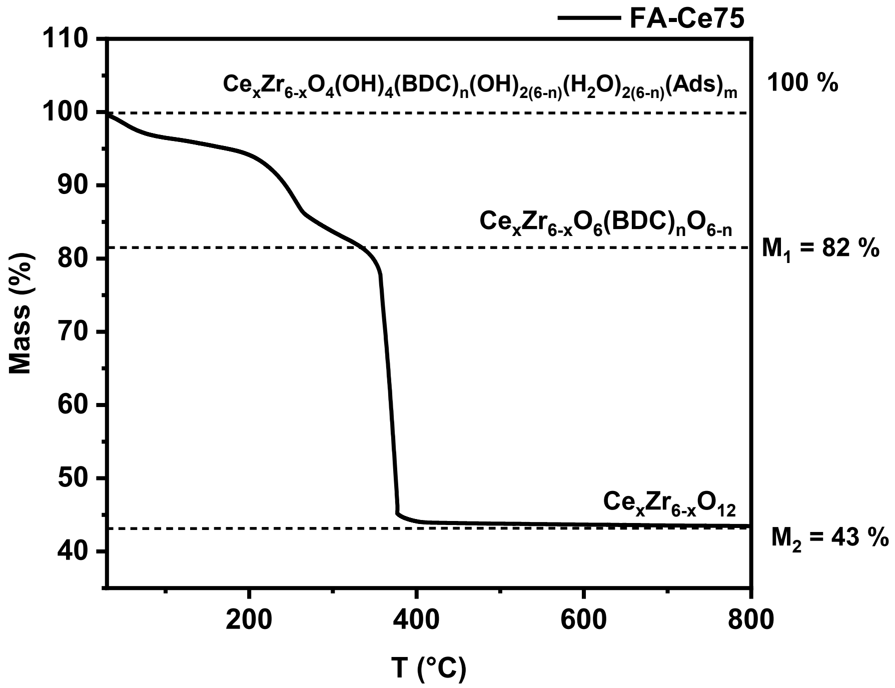

Figure S13 TGA trace of FA-Ce75 with chemical composition described and mass percentage at each stage of decomposition, Ads = adsorbed H_2O, DMF etc.

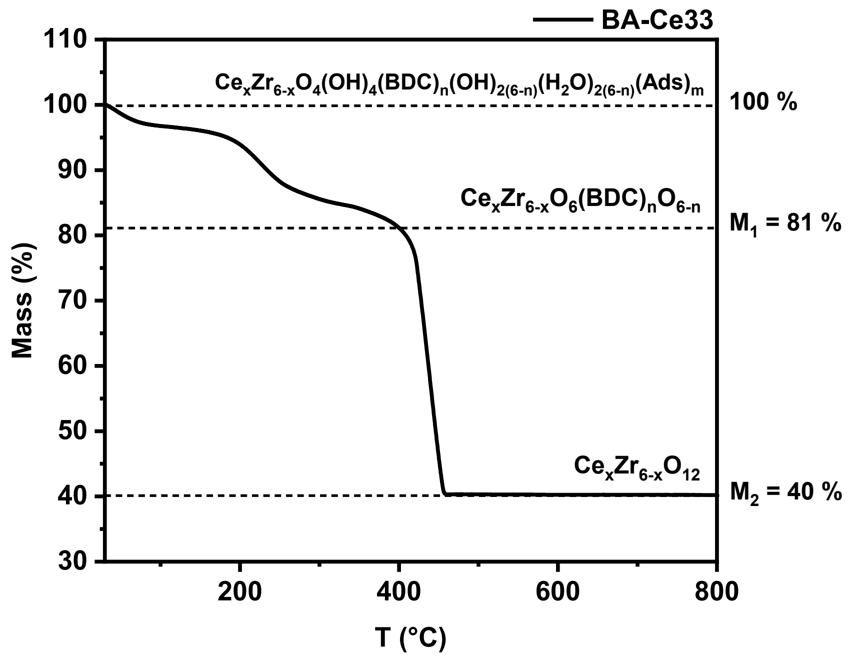

Figure S14 TGA trace of BA-Ce33 with chemical composition described and mass percentage at each stage of decomposition, Ads = adsorbed H_2O, DMF etc.

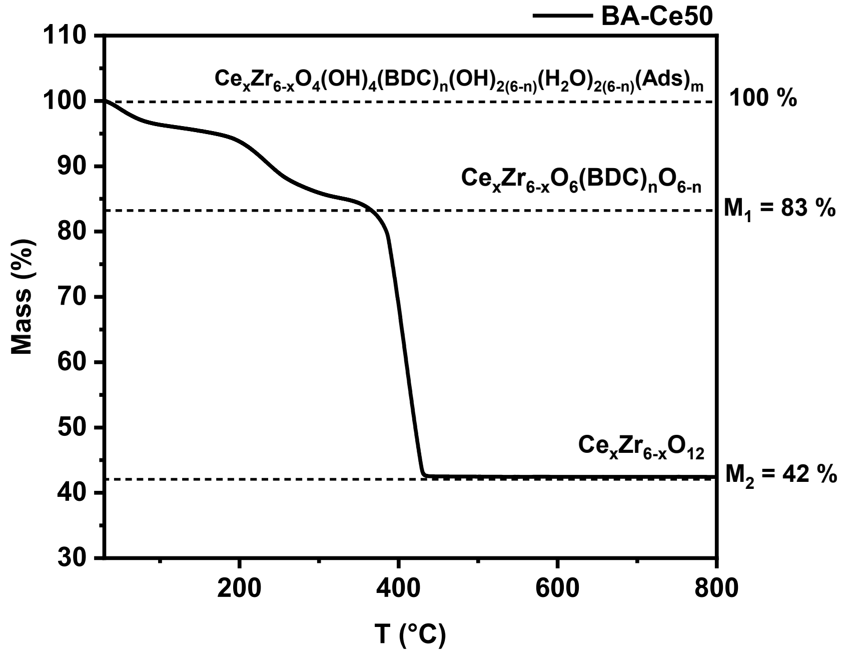

Figure S15 TGA trace of BA-Ce50 with chemical composition described and mass percentage at each stage of decomposition, Ads = adsorbed H_2O, DMF etc.

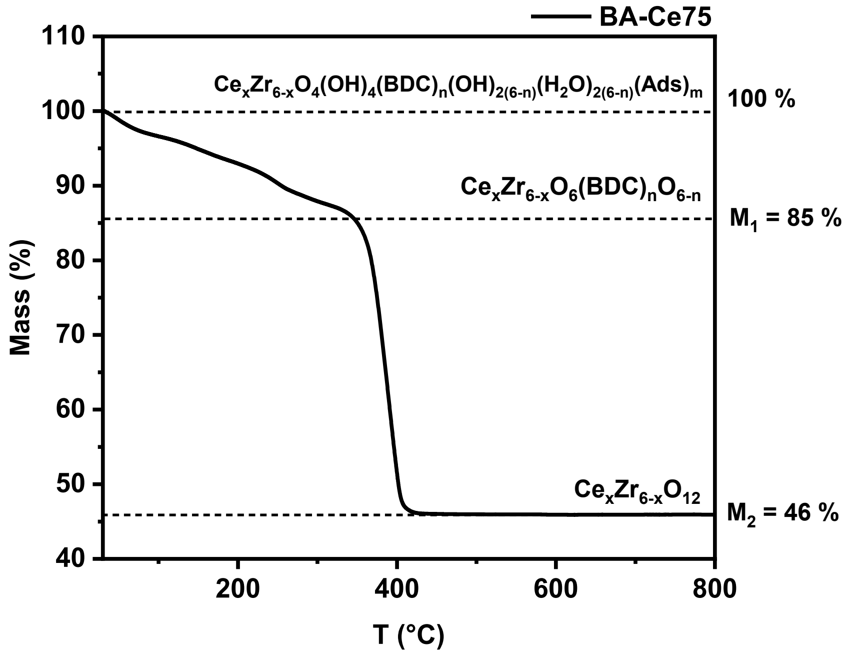

Figure S16 TGA trace of BA-Ce75 with chemical composition described and mass percentage at each stage of decomposition, Ads = adsorbed H_2O, DMF etc.

Table S6 Detailed mass percentages in each decomposition stage, and comparison of Δm from the decomposition of BDC and modulator with the calculated values for a perfect structure as determined by TGA.

Sample	M_1 /%	M_2 /%	Δm /%	$\Delta m_{perfect}$ /%
FA-Ce33	69	35	34	36
FA-Ce50	82	42	40	43
FA-Ce75	82	43	39	42
BA-Ce33	82	40	42	43
BA-Ce50	84	43	41	43
BA-Ce75	86	46	40	41

MOF digestion: 3 mg of a MOF sample was added to 3 mL of 1 M NaOH in D_2O. The mixture was sonicated for 5 min when the solid had dissolved and the remaining solid was removed by centrifuge and then filtration. The ratio of monocarboxylate to BDC is determined by solution NMR.

Table S7 Molar ratios of BDC and benzoate and formate from the MOF digestion determined by ^{1}H NMR

Sample	Molar Ratio			Monocarboxylate : BDC
	BDC	Benzoate	Formate	
FA-Ce50	0.25	0	0.075	0.30 : 1
FA-Ce50 (200 °C)	0.25	0	0.054	0.22 : 1
BA-Ce50	0.92	0.33	0.09	0.46 : 1
BA-Ce50 (200 °C)	0.85	0.33	0.094	0.50 : 1
UiO-66(Ce)	0.25	0	0.17	0.68 : 1
UiO-66(Zr)	0.25	0	0.18	0.72 : 1

S5. TEM

The morphology and elemental mapping of the samples were measured using a JEOL 2100 TEM equipped with LaB_6 operating at 200kV and JEOL ARM200F TEM/scanning TEM (STEM) with a Schottky gun both at 80 kV. Annular dark-field (ADF) STEM measurement was performed in ARM200F, with probe and image aberration CEOS correctors. ADF-STEM images were obtained using a JEOL annular field detector with a probe current of approximately 23 pA, a convergence semi-angle of ~25 mrad, and an inner angle of 45–50 mrad. STEM-EDX analysis was conducted using an Oxford Instruments X-MaxN 100TLE windowless silicon drift detector (SSD).

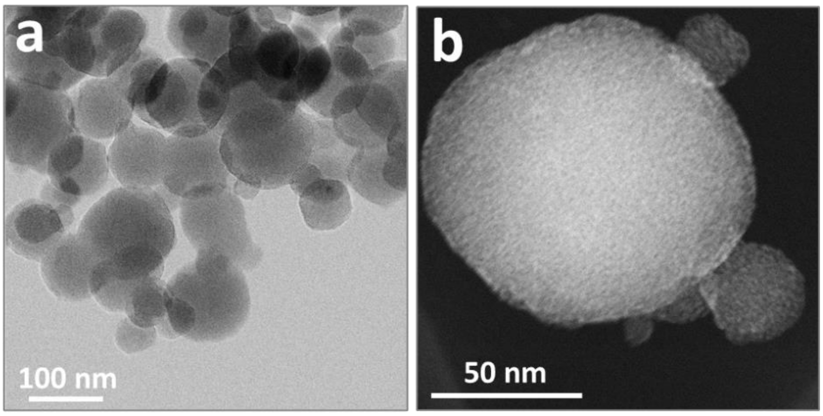

Figure S17 High resolution TEM images of FA-Ce50.

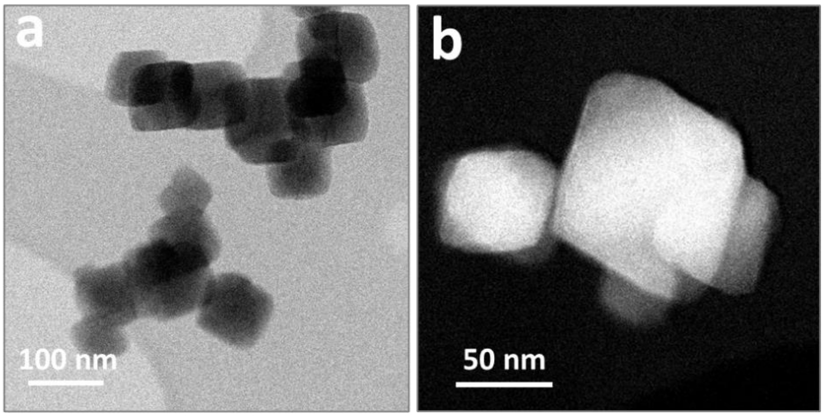

Figure S18 High resolution TEM images of BA-Ce50.

S6. XAFS

S6.1 Experimental Methods

All X-ray absorption spectroscopy experiments including Ce L_{III}-edge, Ce-K edge and Zr K-edge were performed at BM28[5] beamline (XMaS) of European Synchrotron Radiation Facility (ESRF). Ce-K edge and Zr K-edge were performed in transmission mode and the Ce L_{III}-edge was conducted in fluorescence mode. A Si (111) double crystal monochromator was used. The UiO-66 samples were measured in the form of self-supporting 5 mm-diameter pellets at room temperature in transmission mode. The powder sample was mixed with cellulose and pressed. The exact amount of MOFs and cellulose used for the pellet is calculated by XAFSmass software[6] to ensure the concentration is in the correct range.

S6.2 XANES

X-ray absorption near-edge spectroscopy (XANES) was used to investigate the oxidation states of Ce in the MOFs. All XANES data was collected over the edge energy of the Ce L_{III}-edge (5723 eV). The near-edge regions of the spectra are compared against the reference materials. In this experiment, $CeAlO_3$ has been used as the Ce(III) reference and CeO_2 (The National Institute of Standards and Technology (NIST)) as a Ce(IV) reference. The raw sample data were merged and normalised using the software ATHENA from Demeter package to prepare for analysis.[7]

S6.3 EXAFS

$k^3\chi(k)$ EXAFS spectra on Ce K edge and Zr K-edge is shown in Figure S19. The data was normalised and merged using Athena. Different samples show comparable quality of data. The data quality is better when the Ce or Zr concentration is high in the samples. Limited by the signal-to-noise ratio for samples with lower metal content, the Fourier transform was performed up to 16 $Å^{-1}$ for Ce K-edge and 15 $Å^{-1}$ for Zr K-edge. The following EXAFS data fitting was performed using software Artemis.

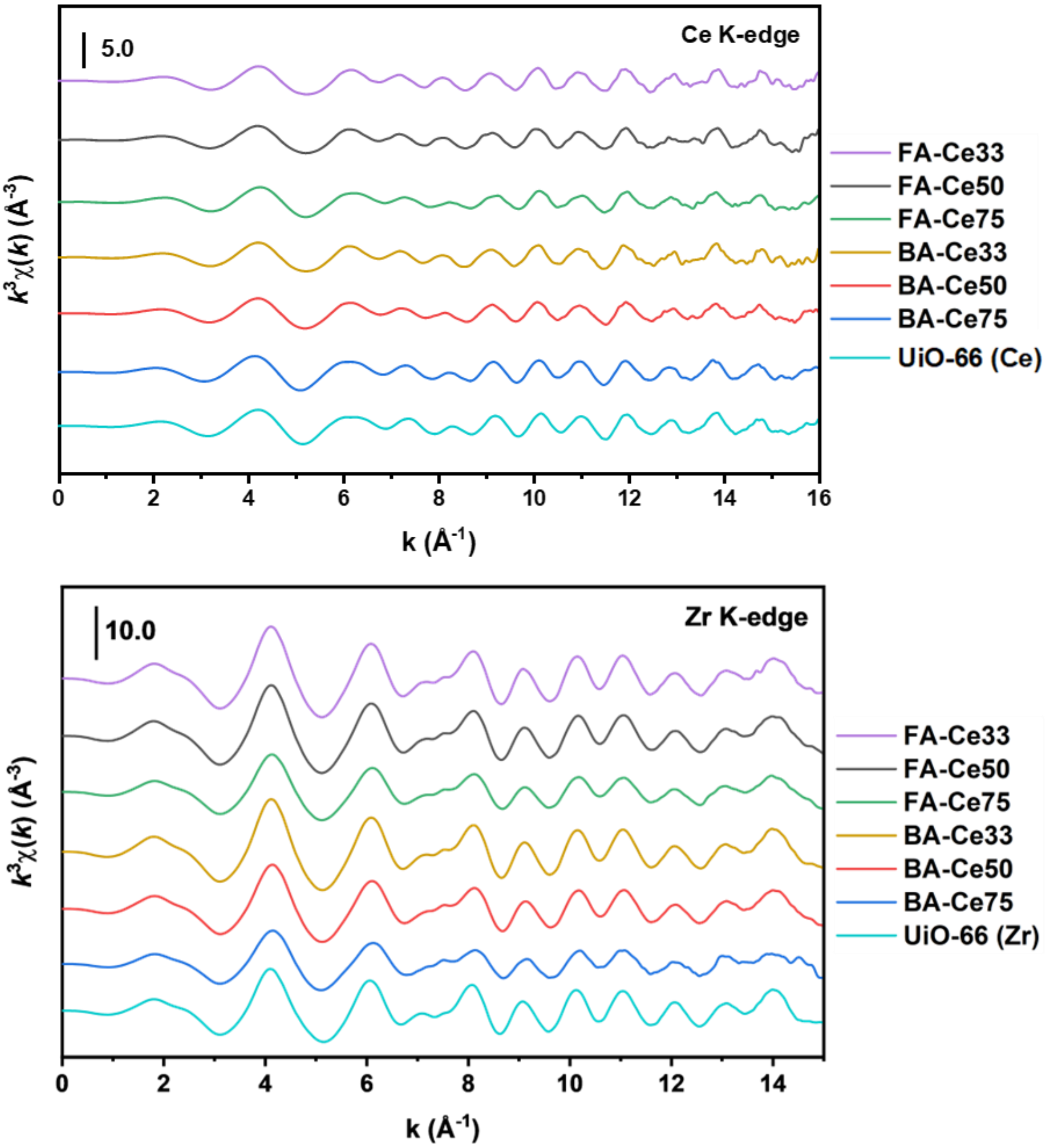

Figure S19 $k^3\chi(k)$ EXAFS data on Ce K-edge (above) and Zr K-edge (below) of Ce/Zr UiO-66 materials and reference pure Ce and Zr UiO-66

S6.3.1 Fitting of UiO-66(Ce) and UiO-66(Zr)

To decrease the variations in the fitting parameters and make the fit more reliable, the referencing pure Ce and Zr UiO-66 samples are fitted first to confirm the amplitude reduction factor S_0^2. The region that shows the signal from M-M scattering was fitted over R = 3.0-3.8 Å for Ce K-edge and R = 2.8-3.8 Å for Zr K-edge spectra.

The results of the best fit of the pure reference samples are shown in Figure S20. The fitted region is between the grey dashed lines. Detailed fitting parameters are shown in Table S8.

Both samples are fitted using an octahedral M_6 cluster model with a shell of 4 nearest neighbours. Debye Waller (DW) factors of both samples are similar, indicating the similarities of structural disorder. S_0^2 factors have been confirmed to be restricted at 0.9 for Ce K-edge spectra and 1.5 for Zr K-edge in the following fits, both close to the expected value of 1.

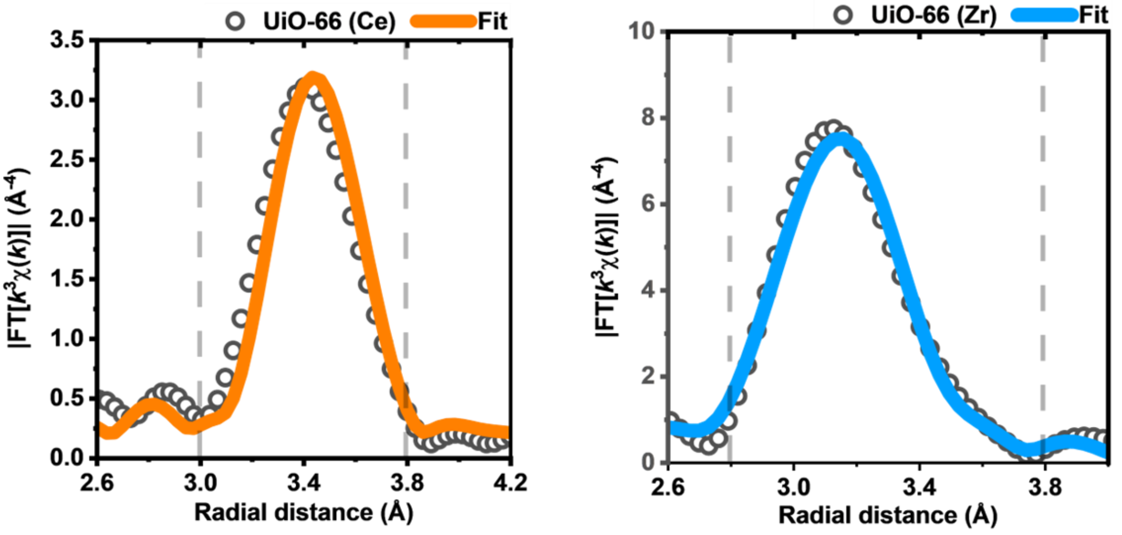

Figure S20 Results of the fitting of the k^3-weighted Fourier-transformed EXAFS of UiO-66(Ce) (left, Ce K-edge, fitted in the range R = 3.0-3.8 Å) and UiO-66(Zr) (right, Zr K-edge, fitted in the range R = 2.8-3.8 Å)

Table S8 Parameters of the best fit of UiO-66(Ce) and UiO-66(Zr) at Ce and Zr K-edges.

	Shell	R / Å	S_0^2	ΔE / eV	σ^2/ Å^2	R-factor
**Ce UiO-66**	4 Ce	3.793 ± 0.004	0.9	-6.2	0.006 ± 0.0001	0.05
**Zr UiO-66**	4 Zr	3.525 ± 0.006	1.5	-4.7	0.007 ± 0.0007	0.04

S6.3.2 Fitting of FA-modulated Samples

Figure S21 demonstrates the model used and the fitting results of FA-Ce33, FA-Ce50 and FA-Ce75. The fitting parameters are shown in Table S9. The fitted data is in good agreement with the measured data with the fitting parameters obtained fall within a reasonable range. The Ce-Zr interatomic distance is fitted to approximately 3.65 Å, which aligns with the values in the literature.[8] Furthermore, from the Ce K-edge perspective, as the Ce content increases, σ^2 for the Ce-Zr bond also increases, suggesting the $CeZr_5$ cluster undergoes distortion with the introduction of higher Ce content.

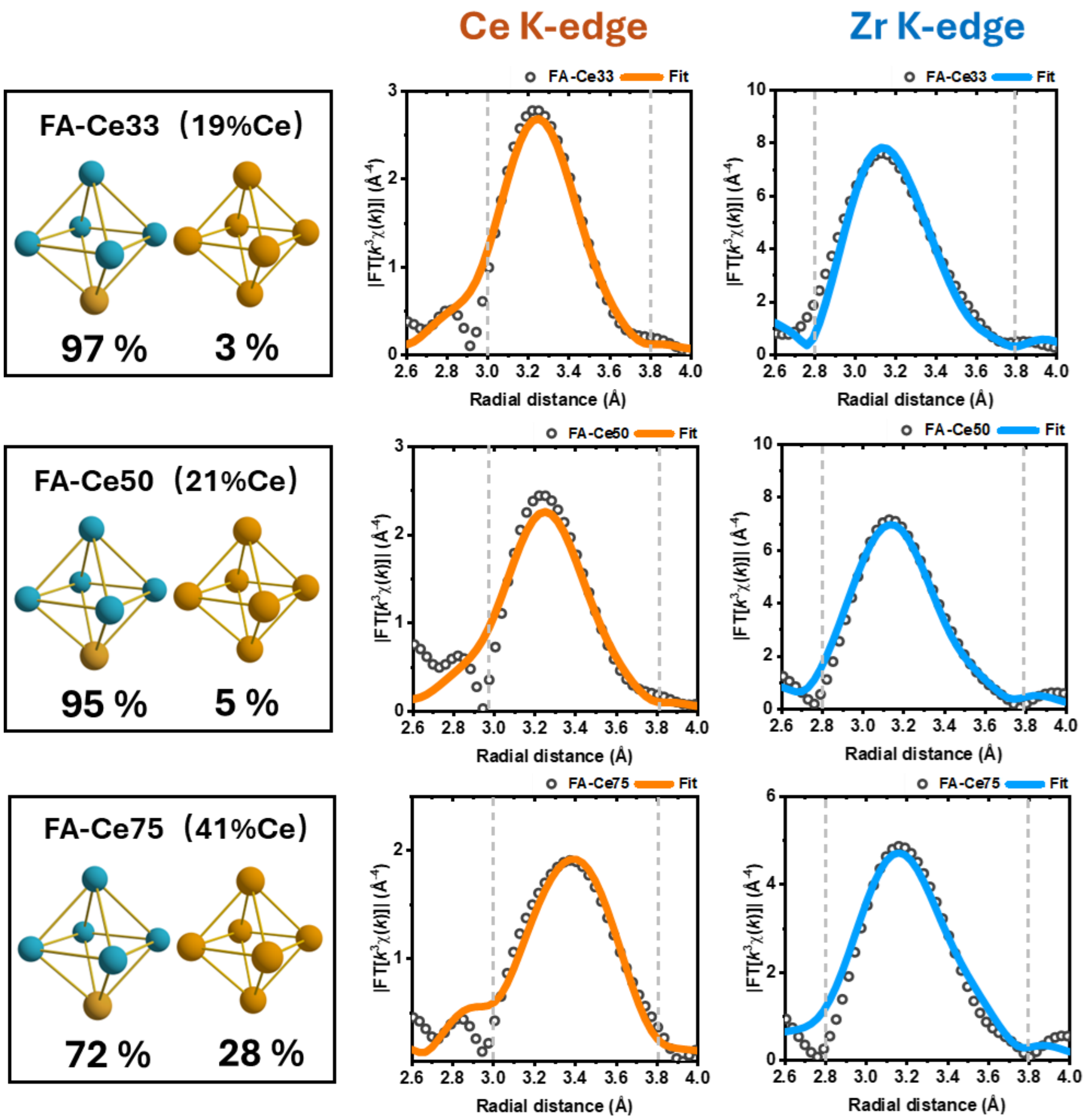

Figure S21 Result of the EXAFS fitting for FA-Ce33, FA-Ce50 and FA-Ce75. Left panels show the cluster composition used in the fitting.

Table S9 EXAFS fitting parameters of the best fits of FA-Ce33, FA-Ce50 and FA-Ce75 at each Ce and Zr K-edges, S_0^2 = 0.9 (Ce K-edge) and S_0^2 = 1.5 (Zr K-edge)

Sample	Edge	Shell	ΔE /eV	R / Å	σ^2 / Å^2	R-factor
FA-Ce33	Ce K	3.88 Zr	-3.5	3.655 ± 0.003	0.004 ± 0.0005	0.07
		0.12 Ce		3.814 ± 0.005	0.006 ± 0.0008	
	Zr K	3.2 Zr	-6.0	3.504 ± 0.003	0.006 ± 0.0005	0.08
		0.8 Ce		3.673 ± 0.006	0.005 ± 0.0006	
FA-Ce50	Ce K	3.84 Zr	-3.4	3.652 ± 0.008	0.007 ± 0.0007	0.11
		0.16 Ce		3.816 ± 0.007	0.004 ± 0.0006	
	Zr K	3.2 Zr	-2.2	3.532 ± 0.011	0.006 ± 0.0003	0.11
		0.8 Ce		3.658 ± 0.004	0.010 ± 0.0010	
FA-Ce75	Ce K	2.88 Zr	-8.7	3.633 ± 0.010	0.010 ± 0.0020	0.12
		1.12 Ce		3.790 ± 0.006	0.004 ± 0.0009	
	Zr K	3.2 Zr	-5.2	3.506 ± 0.009	0.009 ± 0.0010	0.09
		0.8 Ce		3.673 ± 0.003	0.005 ± 0.0005	

S6.3.2 Fitting of BA-modulated Samples

S6.3.2.1 Ce_6 + Zr_6 Model

Taking BA-Ce-50 (34%Ce) as an example, a model with 34% Ce_6 + 66% Zr_6 clusters has been built for the sample. Table S10 shows the fitting results. To achieve a satisfactory fit to the experimental data, a compromise has been made by significantly reducing the interatomic distance *R*(Ce-Ce) to a lower value of 3.712 Å, compared to the fitted result of 3.793 Å for UiO-66(Ce). This suggests the involvement of other species with a lower coordinating distance, which in this case can only be a Ce-Zr path. This observation implies that Zr atoms should be present and interacting with the Ce atoms in the following models.

Table S10 Fitting parameters and a figure of the Ce_6 + Zr_6 model for BA-Ce50. The red entry in the table suggests a wrong parameter given by the current model.

Ce K-edge, fitted over R-range 3.0- 3.8 Å

Shell	R / Å	R_{cryst} / Å	sigma² / Å²
4 Ce	3.712	3.803	0.007

S_0^2 = 0.9, E_0 = -35.2 eV, R = 0.11

Zr K-edge, fitted over R-range 2.8- 3.8 Å

Shell	R / Å	R_{cryst} / Å	sigma² / Å²
4 Zr	3.534	3.556	0.007

S_0^2 = 1.5, E_0 = -4.7 eV, R = 0.12

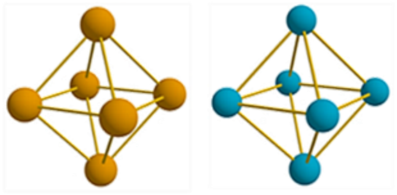

34 % 66 %

S6.3.2.2 $CeZr_5$ Model

The $CeZr_5$ model for FA-modulated samples has also been tried to fit the multiple benzoate-modulated samples. The results are shown in Table S11. The deviated values are highlighted in red in the table. Based on the Ce K-edge analysis of BA-Ce33, the fitting of the Ce-Ce path is challenging due to the low contribution of only 0.16 Ce out of the total 4 atoms in the shell, resulting in the *R*(Ce-Ce) distance to a higher-than-expected value. The fitting analysis of BA-Ce50 using the Zr K-edge data indicates that the DW factor, σ^2 = 0.06 Å², obtained in this fit is higher compared to the results from previous fits. These observations indicate that this model is less suitable for describing the BA-modulated samples compared to the FA-modulated samples.

Table S11 Fitting parameters and the figure of the configuration of $CeZr_5$ model for BA-Ce33, BA-Ce50 and BA-Ce75. Ce K-edge fitted over *R*-range = 3.0-3.8 Å, Zr K-edge fitted over *R*-range = 2.8-3.8 Å. The error values are highlighted in red.

BA-Ce33

Ce K-edge Shell	R / Å	R_{cryst} / Å	sigma² / Å²	Zr K-edge Shell	R / Å	R_{cryst} / Å	sigma² / Å²
3.84 Zr	3.657	3.614	0.007	3.2 Zr	3.515	3.556	0.006
0.16 Ce	3.833	3.803	0.002	0.8 Ce	3.664	3.614	0.003
S_0^2 = 0.9, E_0 = -4.9 eV, R = 0.05				S_0^2 = 1.5, E_0 = -5.2 eV, R = 0.11			

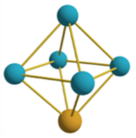
96 %
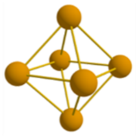
4 %

BA-Ce50

Ce K-edge Shell	R / Å	R_{cryst} / Å	sigma² / Å²	Zr K-edge Shell	R / Å	R_{cryst} / Å	sigma² / Å²
3.16 Zr	3.639	3.614	0.007	3.2 Zr	3.533	3.556	0.06
0.84 Ce	3.813	3.803	0.004	0.8 Ce	3.653	3.614	0.02
S_0^2 =0.9 , E_0 = -4.9 eV, R = 0.08				S_0^2 =1.5 , E_0 = -0.4 eV, R = 0.11			

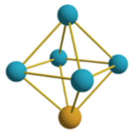
79 %
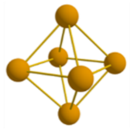
21 %

BA-Ce75

Ce K-edge Shell	R / Å	R_{cryst} / Å	sigma² / Å²	Zr K-edge Shell	R / Å	R_{cryst} / Å	sigma² / Å²
0.88 Zr	3.629	3.614	0.003	3.2 Zr	3.536	3.556	0.010
3.12 Ce	3.813	3.803	0.007	0.8 Ce	3.662	3.614	0.005
S_0^2 = 0.9 , E_0 = -9.0 eV, R = 0.03				S_0^2 = 1.5, E_0 = -2.9 eV, R = 0.12			

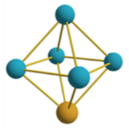
22 %
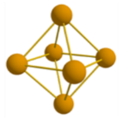
78 %

S6.3.2.3 Ce_2Zr_4 Model (*cis*/ *trans*)

In the Ce_2Zr_4 model of the hexanuclear cluster, there are two possible isomers based on the arrangement of the Ce atoms. These isomers can be described as diagonal isomer (*trans*-Ce_2Zr_4) that two Ce atoms occupy diagonal positions, and adjacent isomer (*cis*-Ce_2Zr_4) in which two Ce atoms are positioned adjacently next to each other.

Using BA-Ce50 as reference sample, the fitting has been conducted for both Ce_2Zr_4 isomers. The results are shown in Table S12. In the *trans*-Ce_2Zr_4 hexanuclear cluster, due to the significant distance between the Ce atoms in this arrangement, the Ce-Ce interaction is considered to be negligible. As a result, the Ce-Ce distance is not considered in the analysis model. It is notable while in this case only Ce-Zr path is included, the resulting fitted distance *R*(Ce-Zr) = 3.69 Å is higher than the expected value of around 3.61 Å. This indicates that the Ce atoms may not be solely interacting with the Zr atoms but also with other neighbouring Ce atoms. In addition, in both fits of Ce and Zr K-edge, the R-factor for *trans*-Ce_2Zr_4 model is higher than that of the *cis*-Ce_2Zr_4 model.

Table S12 Fitting parameters and the composition for *cis-/trans*-Ce_2Zr_4 models of BA-Ce50 (34%) Ce K-edge fitted over *R*-range = 3.0- 3.8 Å, Zr K-edge fitted over R-range = 2.8- 3.8 Å, the error values are highlighted in red.

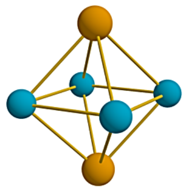

trans-Ce_2Zr_4

Ce K-edge

**Shell**	R / Å	R_{cryst} / Å	sigma^2 / Å^2
4 Zr	3.686	3.614	0.003

S_0^2 = 0.9, E_0 = -1.0 eV, R = 0.14

Zr K-edge

**Shell**	R / Å	R_{cryst} / Å	sigma^2 / Å^2
2 Zr	3.557	3.556	0.006
2 Ce	3.641	3.614	0.03

S_0^2 = 1.5, E_0 = -13.84 eV, R = 0.17

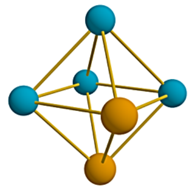

cis-Ce_2Zr_4

Ce K-edge

**Shell**	R / Å	R_{cryst} / Å	sigma^2 / Å^2
2.97 Zr	3.648	3.614	0.006
1.03 Ce	3.806	3.803	0.005

S_0^2 = 0.9, E_0 = -4.9 eV, R = 0.08

Zr K-edge

**Shell**	R / Å	R_{cryst} / Å	sigma^2 / Å^2
2.51 Zr	3.522	3.556	0.005
1.49 Ce	3.649	3.614	0.01

S_0^2 = 1.5, E_0 = -3.4 eV, R = 0.12

When comparing the *cis*-Ce_2Zr_4 model and the $CeZr_5$ model, distinguishing between the two is challenging due to the close proximity of their structures and the small difference in the relative degeneracies of each path. In higher Ce content, Ce_6 contributes to the data with 24 Ce-Ce scattering paths. In contrast, the $CeZr_5$ model includes only 4 Ce-Zr paths, while the *cis*-Ce_2Zr_4 model has 6 Ce-Zr paths. Sample BA-Ce33, with lower Ce content (23%), shows the most distinctive differences between the models from the Ce K-edge and has been used for fitting to both models.

The comparison between the EXAFS fitting parameters of *cis*-Ce_2Zr_4 and $CeZr_5$ model for BA-Ce33 is shown in Table S13. In the fitting of the Ce K-edge data, the $CeZr_5$ model yields a higher *R*(Ce-Ce) value and a lower σ^2 compared to the *cis*-Ce_2Zr_4 model. The higher *R*(Ce-Ce) distance in the $CeZr_5$ model suggests the need for additional Ce-Ce scattering paths. This issue is resolved in the *cis*-Ce_2Zr_4 model, where the Ce-Ce distances better match the expected data. The DW factor in the *cis*-Ce_2Zr_4 model falls within a reasonable range, further supporting its validity.

Table S13 EXAFS Fitting parameters and the composition of *cis*-Ce_2Zr_4 and $CeZr_5$ model for BA-Ce33 (23 %Ce), Ce K-edge fitted over *R*-range = 3.0-3.8 Å, Zr K-edge fitted over R-range = 2.8-3.8 Å. The error values are highlighted in red.

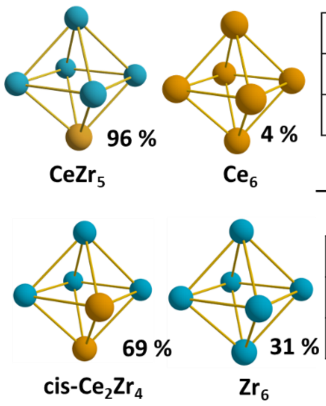

$CeZr_5$ (96 %) / Ce_6 (4 %)

Ce K-edge

Shell	R / Å	R_{cryst} / Å	sigma^2 / Å^2
3.84 Zr	3.657	3.614	0.007
0.16 Ce	3.843	3.803	0.002

S_0^2 = 0.9, E_0 = -4.9 eV, R = 0.05

Zr K-edge

Shell	R / Å	R_{cryst} / Å	sigma^2 / Å^2
3.2 Zr	3.515	3.556	0.006
0.8 Ce	3.664	3.614	0.003

S_0^2 = 1.5, E_0 = -5.2 eV, R = 0.11

cis-Ce_2Zr_4 (69 %) / Zr_6 (31 %)

Ce K-edge

Shell	R / Å	R_{cryst} / Å	sigma^2 / Å^2
3 Zr	3.654	3.614	0.005
1 Ce	3.800	3.803	0.01

S_0^2 = 0.9, E_0 = -5.9 eV, R = 0.05

Zr K-edge

Shell	R / Å	R_{cryst} / Å	sigma^2 / Å^2
2.96 Zr	3.515	3.556	0.006
1.04 Ce	3.661	3.614	0.009

S_0^2 = 1.5, E_0 = -5.2 eV, R = 0.11

S6.3.2.4 Ce_3Zr_3 Model (*fac*/ *mer*)

Two possible isomers of the Ce_3Zr_3 cluster, the *mer*- and *fac*-configurations, were considered in the model. Using BA-Ce75 as an example, fitting was conducted for both Ce_3Zr_3 isomers, and the results are shown in Table S14. From the Ce K-edge, the difference in the relative degeneracies of each path is small compared to the *cis*-Ce_2Zr_4. However, from the Zr K-edge, there is significant deviation to lower values from the expected R(Ce-Zr) for both *mer*-Ce_3Zr_3 and *fac*-Ce_3Zr_3. Additionally, the DW factor is much higher than the fitting results from the *cis*-Ce_2Zr_4 model. This suggests that the Ce content is overestimated in this model. Therefore, considering the contribution of Ce in Zr-Ce path, models with higher Ce content, such as Ce_4Zr_2, are ruled out.

Table S14 EXAFS Fitting parameters and the composition of *mer*-Ce_3Zr_3 and *fac*-Ce_3Zr_3 model for BA-Ce33 (23 %Ce), Ce K-edge fitted over *R*-range = 3.0-3.8 Å, Zr K-edge fitted over R-range = 2.8-3.8 Å The error values are highlighted in red.

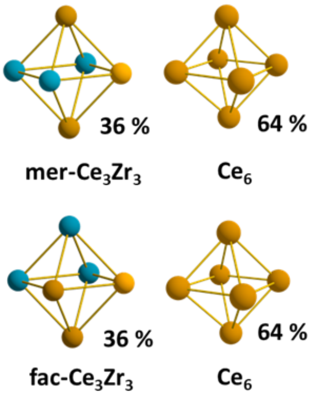

mer-Ce_3Zr_3 (36 %) / Ce_6 (64 %)

Ce K-edge

Shell	R / Å	R_{cryst} / Å	sigma^2 / Å^2
0.96 Zr	3.628	3.614	0.004
3.04 Ce	3.812	3.803	0.007

S_0^2 = 0.9, E_0 = -4.9 eV, R = 0.05

Zr K-edge

Shell	R / Å	R_{cryst} / Å	sigma^2 / Å^2
1.28 Zr	3.536	3.556	0.004
2.77 Ce	3.512	3.614	0.02

S_0^2 = 1.5, E_0 = 3.7 eV, R = 0.08

fac-Ce_3Zr_3 (36 %) / Ce_6 (64 %)

Ce K-edge

Shell	R / Å	R_{cryst} / Å	sigma^2 / Å^2
0.72 Zr	3.629	3.614	0.003
3.28 Ce	3.815	3.803	0.007

S_0^2 = 0.9, E_0 = -8.3 eV, R = 0.05

Zr K-edge

Shell	R / Å	R_{cryst} / Å	sigma^2 / Å^2
2 Zr	3.541	3.556	0.005
2 Ce	3.567	3.614	0.02

S_0^2 = 1.5, E_0 = 4.3 eV, R = 0.09

S6.4 Solution XAFS

A liquid cell has been used for the measurement of solution XAFS. This plastic cell features inlet and outlet for liquid injection and is sealed with DuPont™ Kapton® polyimide film to contain the liquid where the beam passes through.

To measure the solution after the reaction, the reaction was stopped by cooling to room temperature, and the solution was separated from the solid product by centrifugation. The separated solution was then injected into the liquid cell for measurement.

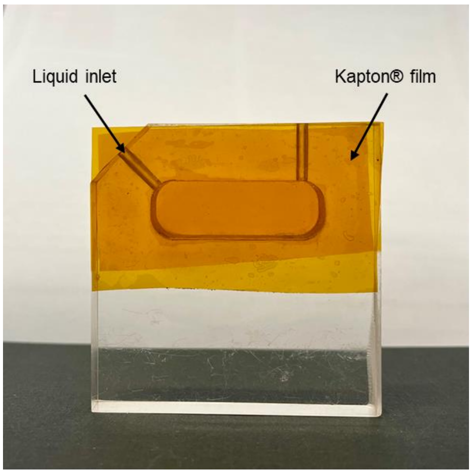

Figure S22 Perspex® liquid cell for the measurement of solution XAFS.

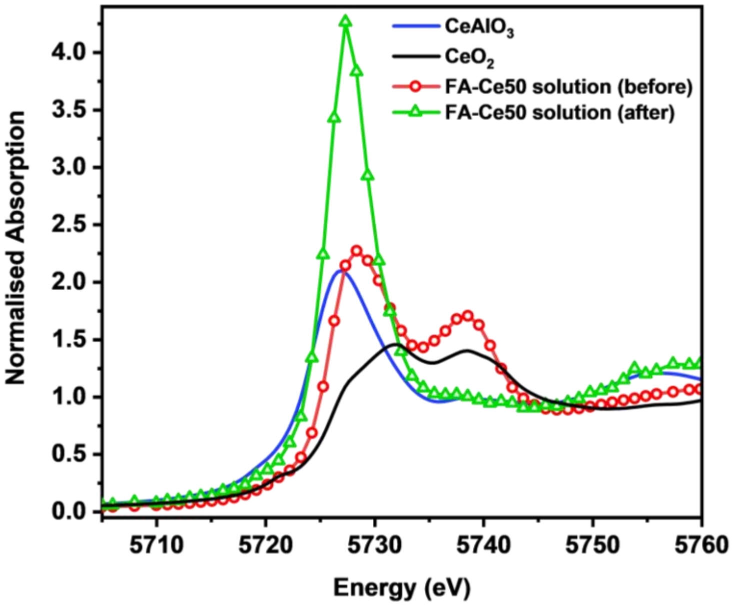

Figure S23 Ce L_{III}-edge XANES spectra of the synthesis solutions for FA-Ce50 before and after the reaction compared to the Ce(III) and Ce(IV) reference materials.

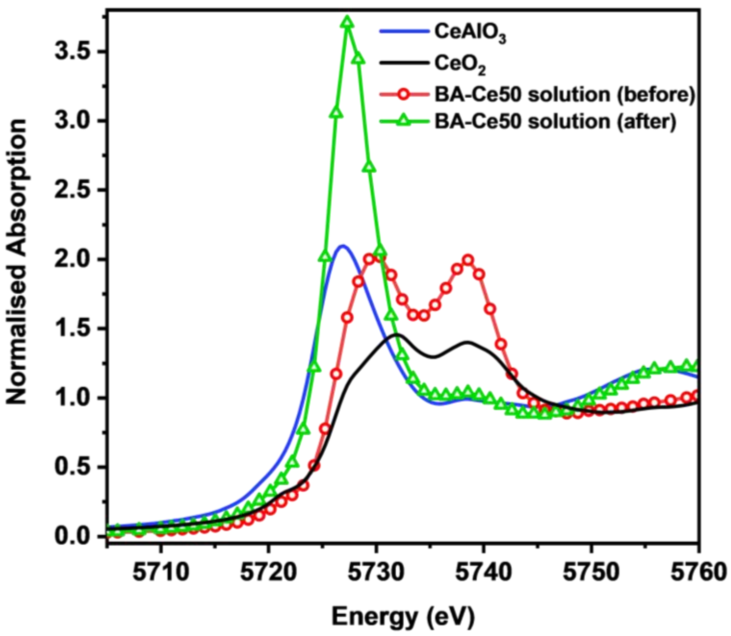

Figure S24 Ce L$_{III}$-edge XANES spectra of the synthesis solutions for FA-Ce50 before and after the reaction compared to the Ce(III) and Ce(IV) reference materials.

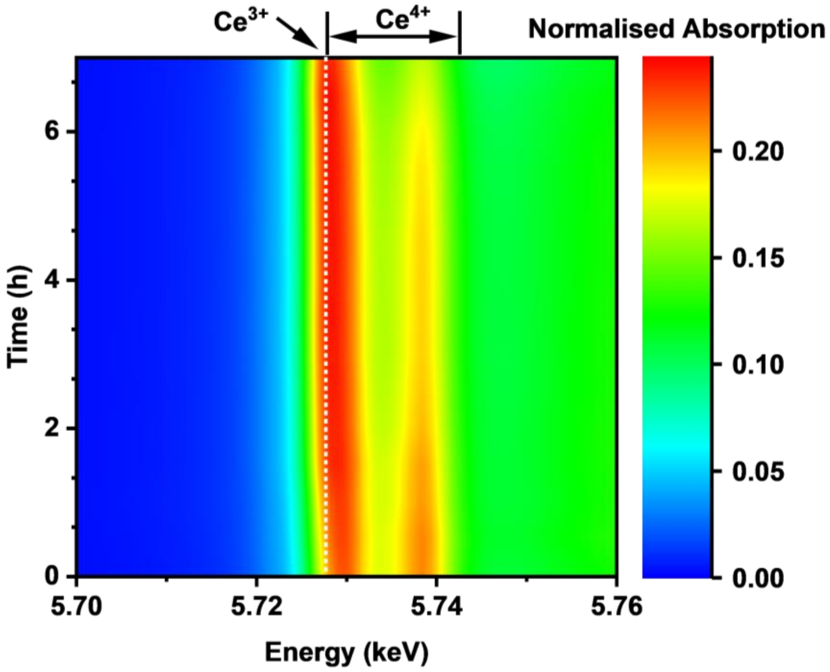

Figure S25 Contour plot of Ce L$_{III}$-edge XANES spectra of the test solution ($(NH_4)_2Ce(NO_3)_6$ in DMF) kept under room temperature for 7 hours. The XANES spectra was measured every 27 min.

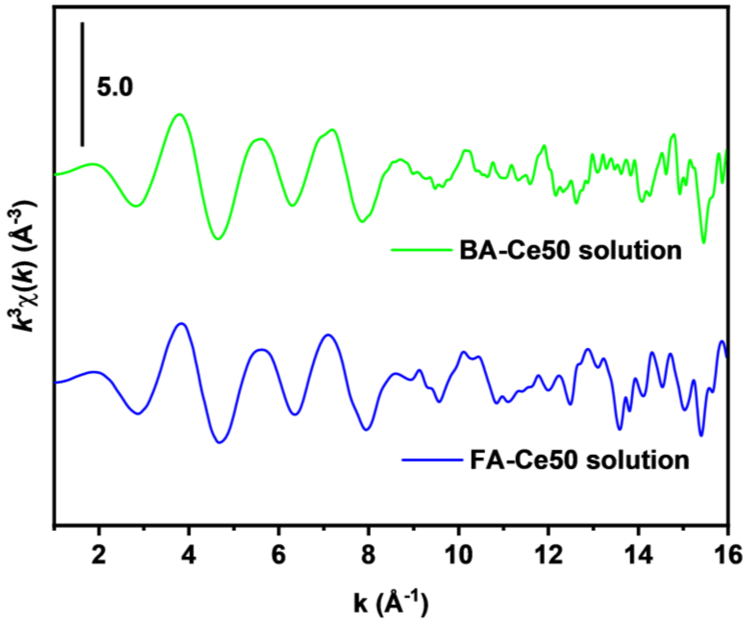

Figure S26 $k^3\chi(k)$ Ce K-edge EXAFS data of the synthesis solution of BA-Ce50 and FA-Ce50 after reaction.

Table S15 Ce K-edge EXAFS fitting parameters of FA-Ce50 and BA-Ce50 solution after reaction, $S_0^2 = 0.9$

Sample	Shell	R / Å	ΔE /eV	σ /Å^2	R-factor
**FA-Ce50 solution**	9 O	2.5163 ± 0.004	-0.4 ± 1.3	0.011 ± 0.0009	0.03
**BA-Ce50 solution**	9 O	2.5220 ± 0.009	-2.4 ± 2.2	0.011 ± 0.0016	0.08

S7. FTIR Spectroscopy

A sample cell was designed capable of operating the measurement across a broad temperature range and accommodating various gas atmospheres. The cell features transparent windows made of CaF_2, which enables the transmission of infrared radiation into and out of the cell. Within the cell, a sample holder securely holds the sample pellet, allowing the infrared radiation to pass through for analysis. The cell is surrounded by a heating jacket equipped with a thermocouple for temperature control. The cell also includes ports or fittings for the controlled introduction and removal of gases.

In this experiment, the measurement was conducted under an Ar gas flow. The samples were mixed with KBr and pressed to the form of thin self-supporting pellets before measuring. The samples were heated *in-situ* to a temperature of 110 °C while being exposed to a flow of Ar to remove any adsorbed or loosely bound water molecules present in the samples. The data was collected until the O-H stretching at 3300 cm^{-1} from water was reduced to a minimum.

The data were collected using a Shimadzu IRTracer-100 instrument spectrometer at 2 cm^{-1} resolution. The LabSolutions IR software was employed to monitor the spectra in real-time and analyse the obtained results.

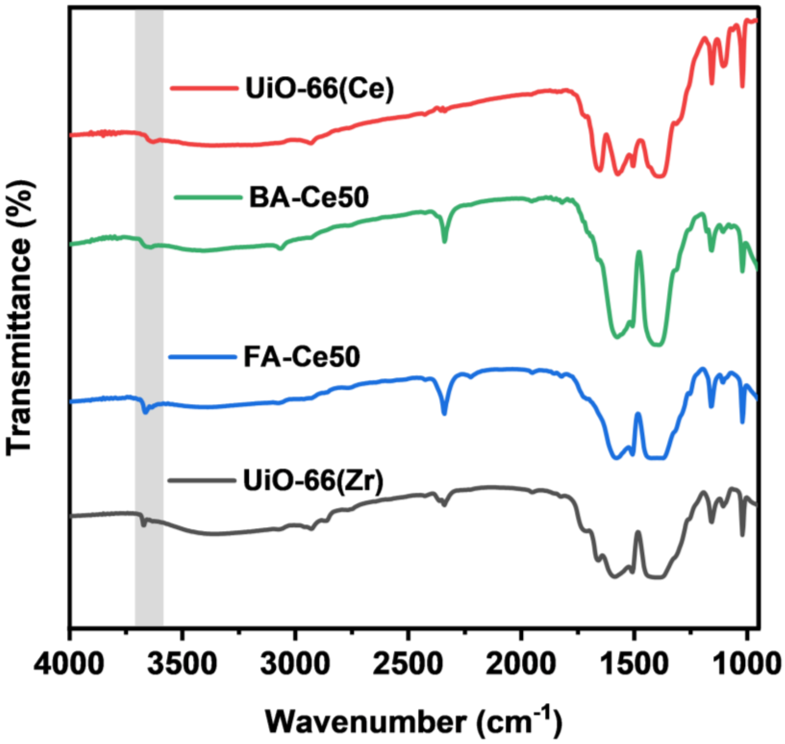

Figure S27 IR spectra for samples BA-Ce50, FA-Ce50, UiO-66(Ce), and UiO-66(Zr) after water removal by heating at 110 °C in a flow of Ar. The grey region highlights the ν(OH) bands of bridging μ_3-OH groups.

S8. N_2 Sorption Experiments

Porosity measurements of the MOF materials were carried out using an ASAP 2020 gas adsorption instrument. To measure the surface area of MOFs using N_2 gas sorption analysis, the material is typically pretreated by degassing under vacuum at 120°C for 4 hours to remove any adsorbed gases or volatile impurities. To calculate the BET specific surface area from a BET plot, the standard pressure (P/P_0) range of 0.05–0.20 is selected.[9] All synthesised UiO-66 samples show microporosity, characterised by type-I isotherms. The results of BET surface area and the pore volume are shown in Table S16.

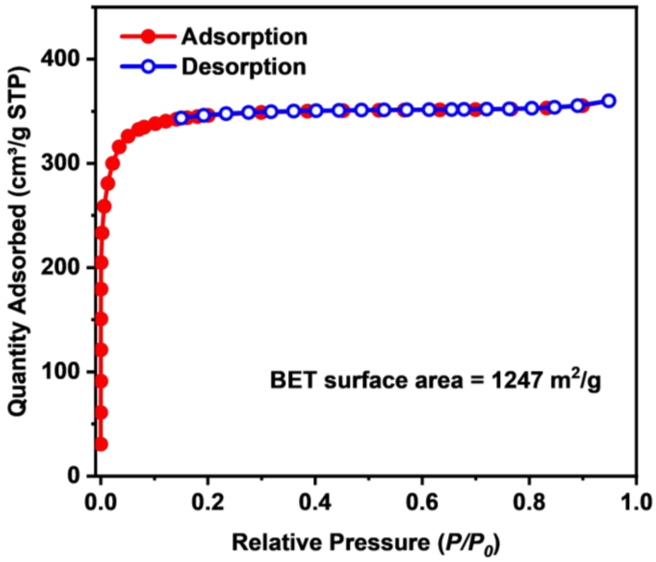

Figure S28 N_2 adsorption-desorption isotherms at 77K of UiO-66(Zr).

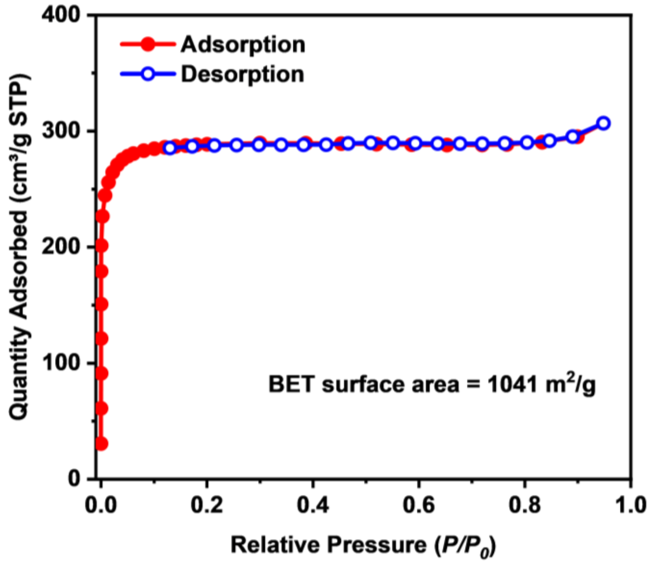

Figure S29 N_2 adsorption-desorption isotherms at 77K of UiO-66(Ce).

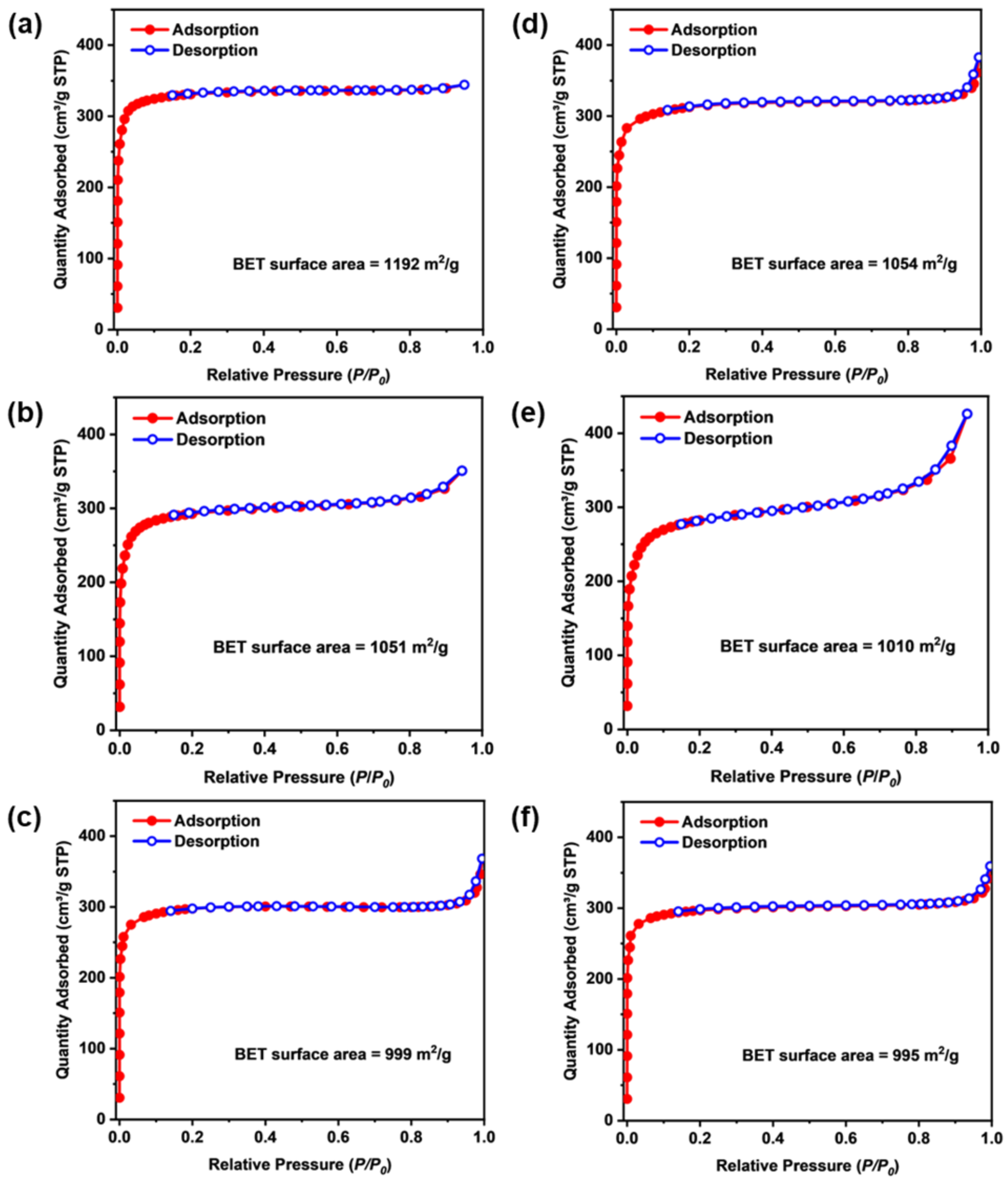

Figure S30 N_2 adsorption-desorption isotherms at 77K of sample FA-Ce33 (a), FA-Ce50 (b), FA-Ce75 (c), BA-Ce33 (d), BA-Ce50 (e) and BA-Ce75 (f)

Table S16 BET surface area and micropore volume of FA-Ce33, FA-Ce50, FA-Ce75, BA-Ce33, BA-Ce50, BA-Ce75 and UiO-66(Zr) and UiO-66(Ce) obtained from the isotherms.

Sample	BET surface area (m^2/g)	Micropore volume (cm^3/g)
UiO-66(Ce)	1041	0.40
UiO-66(Zr)	1247	0.45
FA-Ce33	1192	0.44
FA-Ce50	1051	0.35
FA-Ce75	999	0.34
BA-Ce33	1054	0.41
BA-Ce50	1010	0.33
BA-Ce75	995	0.31

S9. XPS

The XPS measurements were carried out using a Kratos Axis Ultra DLD spectrometer. Samples were affixed to a stainless-steel bar using electrically conductive carbon tape. The XPS spectrometer's base pressure was approximately 1×10^{-10} mbar, and the measurements were carried out under a chamber pressure of less than 1×10^{-9} mbar. A monochromated Al K_{α} X-ray source (1.487 keV) was employed for the XPS measurements. The measurements were performed at room temperature with the aid of a charge neutraliser, and the take-off angle was set at 90° relative to the sample surface. All XPS spectra were calibrated using the C1s core level.

To extract peak positions and relative stoichiometries, fitting procedures were executed employing the Casa XPS software suite. Corrections were made considering the sensitivity factors, photoelectron mean free paths, and photo-ionisation cross sections of the respective core levels.

In the fitting, the binding energy separation between Ce^{3+} and Ce^{4+} states is typically constrained within a certain range. The fitting data is shown in Table S17. The designated binding energy for each peak was referenced to reported literature.[10]

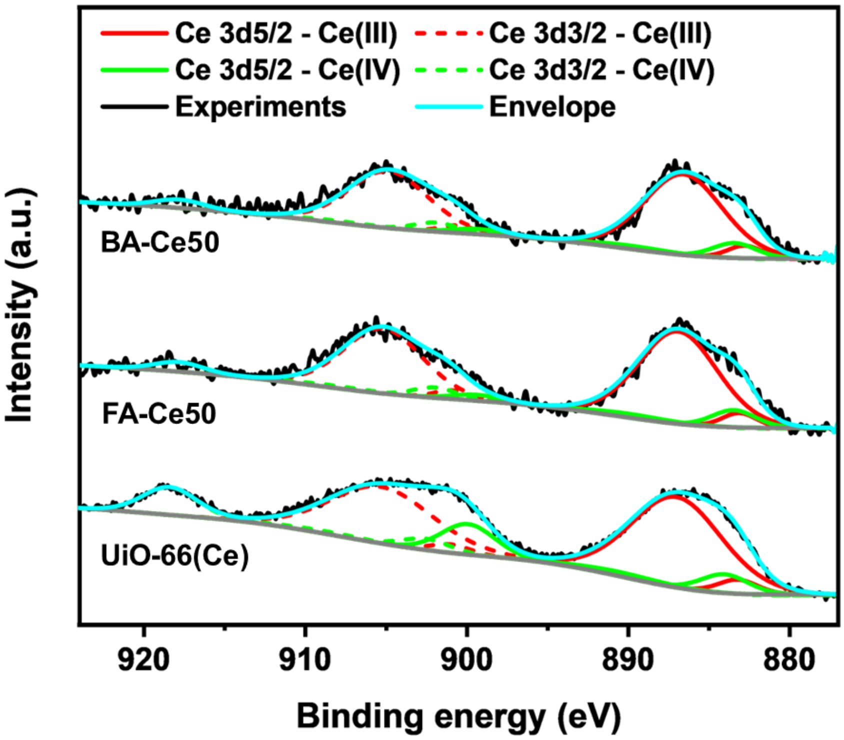

Figure S31 Ce $3d_{3/2}$ and $3d_{5/2}$ XPS spectra of BA-Ce50 and FA-Ce50 in comparison with UiO-66(Ce)

Table S17 XPS binding energies (eV) and relative area content (%) of individual peaks of Ce 3d for BA-Ce50 and FA-Ce50 compared to pure UiO-66(Ce)

		**BA-Ce50**		**FA-Ce50**		**UiO-66(Ce)**	
	Peak assignment	Binding Energy (eV)	Area (%)	Binding Energy (eV)	Area (%)	Binding Energy (eV)	Area (%)
**Ce $3d_{5/2}$**	Ce (III)	882.76	2.6	883.1	2.6	883.11	2.3
	Ce (IV)	883.4	4	883.45	4	883.93	3.7
	Ce (III)	886.56	38.7	886.9	38.7	886.91	34.1
	Ce (IV)	890	1.7	890.05	1.7	890.53	1.6
	Ce (IV)	899.3	1.6	899.35	1.6	899.83	6.9
**Ce $3d_{3/2}$**	Ce (III)	900.56	2.3	900.9	2.3	900.91	2.1
	Ce (IV)	901.9	3.5	901.95	3.5	902.43	3.3
	Ce (III)	904.86	40.4	905.2	40.3	905.21	35.5
	Ce (IV)	908.4	1.5	908.45	1.5	908.93	1.4
	Ce (IV)	917.8	3.8	917.85	3.8	918.33	9.3
**Ce(IV) content**		**16%**		**16%**		**26%**	

S10. Catalysis Studies

S10.1 Experimental Methods

The catalyst was firstly activated by degassing at 200 °C under vacuum for 2 hours. For UiO-66(Ce), the activation temperature was adjusted to 180°C due to its lower thermal stability. After the catalyst was cooled to room temperature it was promptly used in the reaction.

In a typical experiment, 81 mg of benzyl alcohol (0.75 mmol) and 6 mol% of activated catalyst was added to 2.5 mL of acetonitrile in a glass microwave reaction tube. Then, 0.2 mL of ^{t}BuOOH solution (5.0-6.0 M in decane) was added to this mixture. The tube was then sealed and sonicated for 10 minutes. After sonication, the reaction tube was transferred to an Anton-Parr Monowave 200 microwave reactor and heated at the maximum rate to reach 90 °C with magnetic stirring, maintaining this temperature for 10 minutes.

After the reaction mixture cooled to room temperature, approximately 100 mg of the internal standard 1,4-dioxane was accurately weighed and thoroughly mixed into the solution. The mixture was then centrifuged to separate the solution from the solid catalyst. The resulting solution was filtered, added to an NMR tube, and diluted with $CDCl_3$ for the following NMR measurement.

^{1}H NMR spectroscopy was used to quantify the product of the reaction. The data was collected by a Bruker AVIII HD-300 MHz spectrometer. The quantitative analysis was performed on a Bruker TopSpin NMR Analysis software. Samples were spiked with a known exact molar quantity of an internal standard (1,4-dioxane). The molar quantity of benzaldehyde $m_{Benzaldehyde}$ in the sample was calculated using Equation S2 as shown below:

$$m_{Benzaldehyde} = \frac{I_{analyte} \times P_{standard}}{I_{standard} \times P_{analyte}} \times m_{standard} \quad \text{(Equation S2)}$$

where $m_{standard}$ is the known molar amount of the internal standard 1,4-dioxane, $I_{analyte}$ and $I_{standard}$ represent the integral of the resonance peaks for the analyte and the standard, and $P_{standard}$ and $P_{analyte}$ are the numbers of protons corresponding to the resonance peak.

S10.2 Optimisation Studies

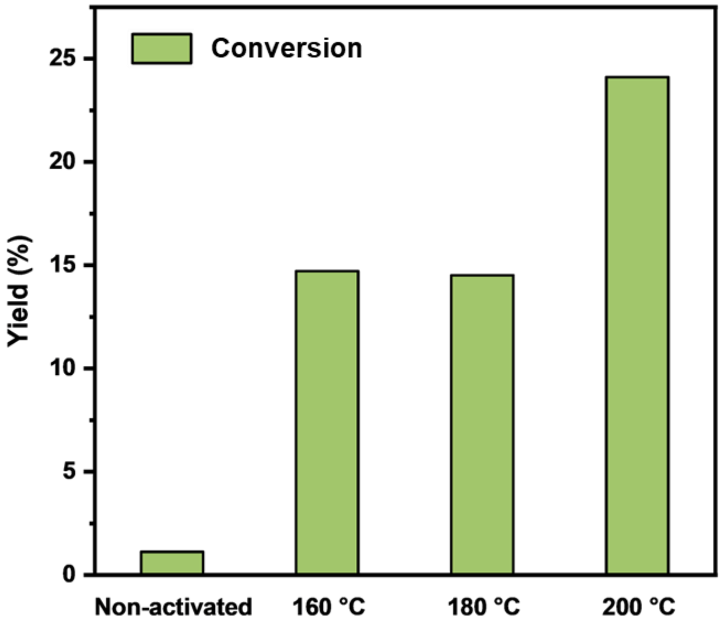

Figure S32 Conversion of the oxidation of benzyl alcohol by BA-Ce50 that was activated under vacuum condition at different temperatures for 2 hours. Reaction condition: 0.75 mmol benzyl alcohol, 1.1 mmol ^{t}BuOOH, 2.5 mL acetonitrile, heated in microwave at 90°C for 10 minutes. Catalyst: BA-Ce50

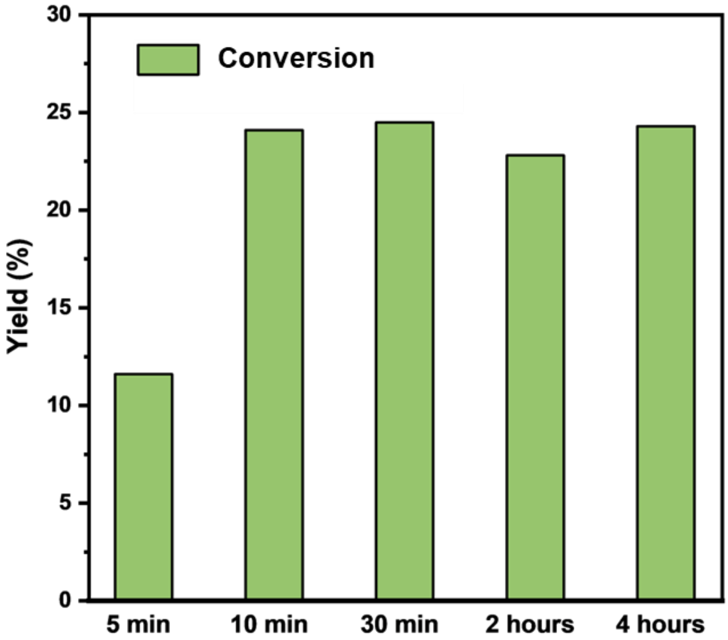

Figure S33 Conversion of the oxidation of benzyl alcohol when using various reaction time. Reaction condition: 0.75 mmol benzyl alcohol, 1.1 mmol ^{t}BuOOH, 2.5 mL acetonitrile, catalyst: BA-Ce50. Reactions for 5, 10, and 30 min were carried out by microwave heating and that of 2 and 4 hours were performed by autoclave in an oven. The temperature performed was 90°C.

S10.3 Recyclability and Stability Test

For the recyclability test, the solid catalyst separated through centrifugation was washed three times with acetone. Subsequently, it was soaked in acetone overnight to ensure complete solvent exchange from the compounds trapped within the MOF's pores. To prepare the catalyst for reuse, it was dried in an oven at 70°C and then re-activated at 200°C for 2 hours under vacuum.

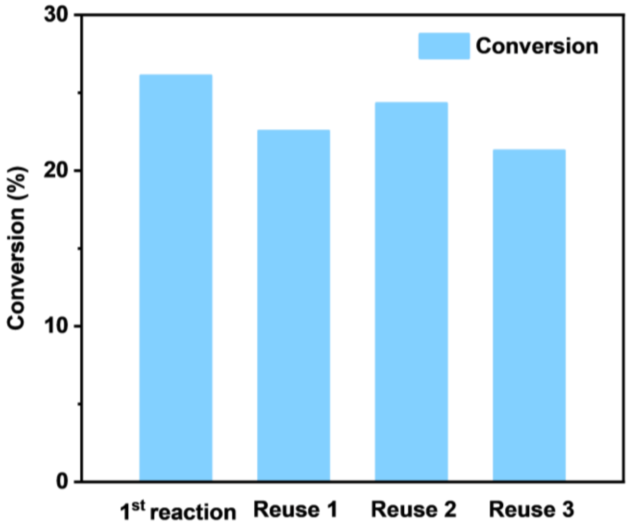

Figure S34 Results of catalytic oxidation of benzyl alcohol by BA-Ce50 across four consecutive reaction cycles reusing the same catalyst. Reaction condition: 0.75 mmol benzyl alcohol, 1.1 mmol ^{t}BuOOH, 2.5 mL acetonitrile, heated in microwave at 90°C for 10 minutes. Catalyst: BA-Ce50.

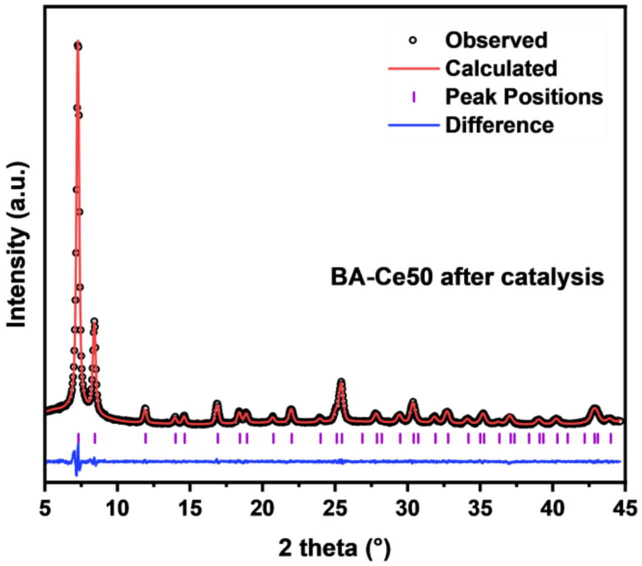

Figure S35 Pawley plot of BA-Ce50 after catalytic reaction. Reaction condition: 0.75 mmol benzyl alcohol, 1.1 mmol ^{t}BuOOH, 2.5 mL acetonitrile, heated in microwave at 90°C for 10 minutes.

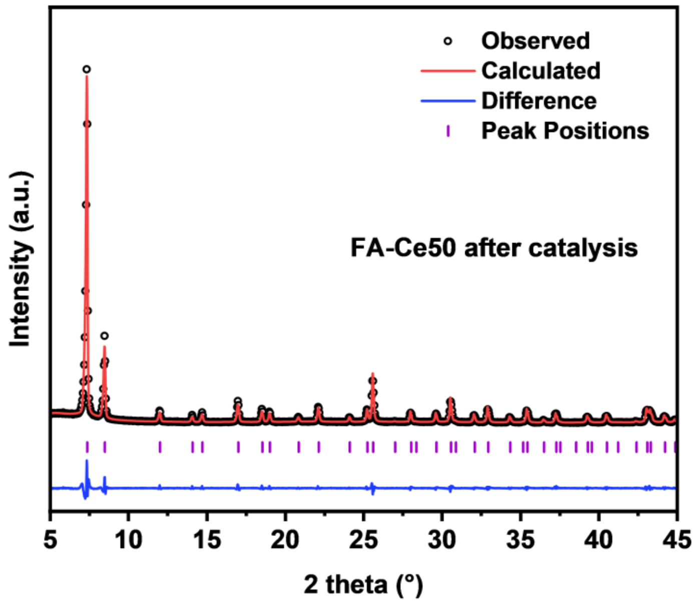

Figure S36 Pawley plot of FA-Ce50 after catalytic reaction. Reaction condition: 0.75 mmol benzyl alcohol, 1.1 mmol ^{t}BuOOH, 2.5 mL acetonitrile, heated in microwave at 90°C for 10 minutes.

Table S18 Lattice parameters of the materials before and spent from catalytic reactions. Results are obtained from Pawley fits.

Compound	*a* (Å)	α (°)	Space group	R_{wp} (%)	GoF
BA-Ce50	20.9897(5)	90	$Fm\bar{3}m$	4.5	1.3
BA-Ce50 after catalysis	20.9811(9)	90	$Fm\bar{3}m$	5.2	1.4
FA-Ce50	20.9187(5)	90	$Fm\bar{3}m$	5.0	1.3
FA-Ce50 after catalysis	20.9061(4)	90	$Fm\bar{3}m$	7.8	2.4

S10.4 ^{1}H NMR Spectra

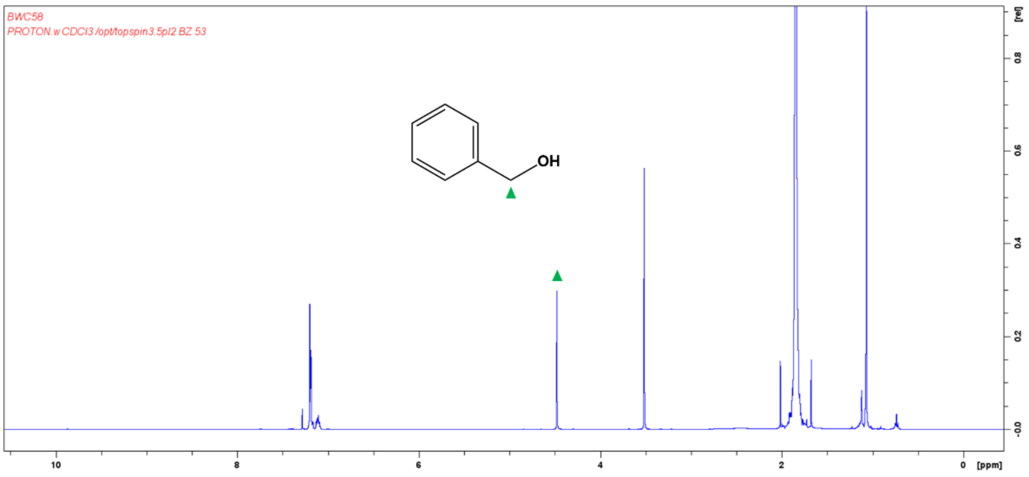

Figure S37 ^{1}H NMR spectra of post experimental solution of the blank control reaction.

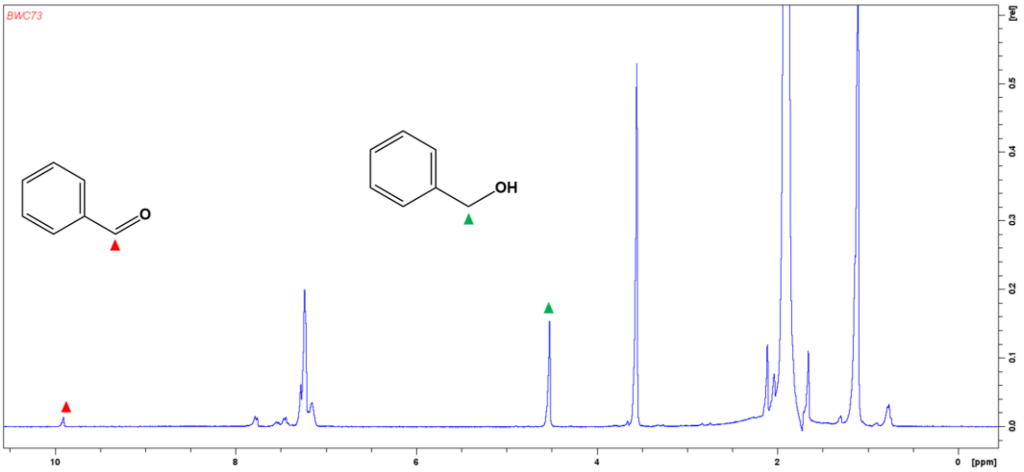

Figure S38 ^{1}H NMR spectra of post catalysis reaction solution catalysed by BA-Ce33.

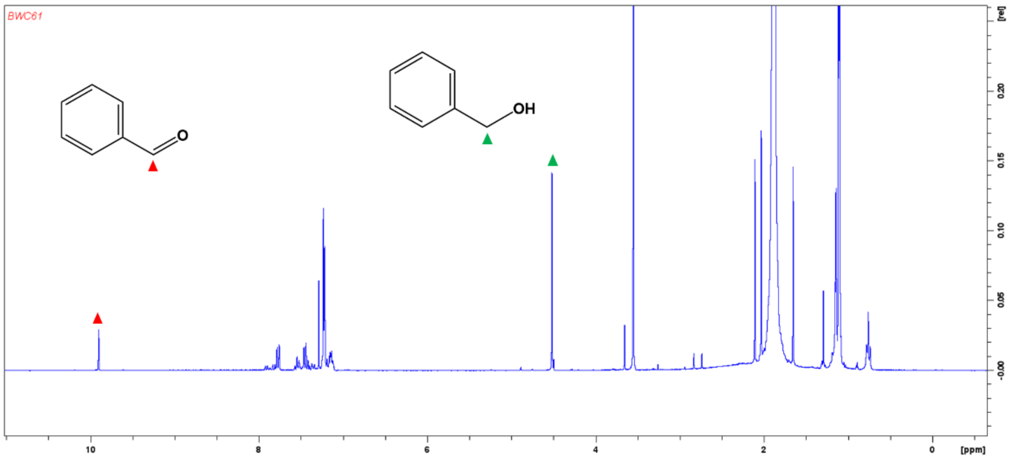

Figure S39 ^{1}H NMR spectra of post catalysis reaction solution catalysed by BA-Ce50.

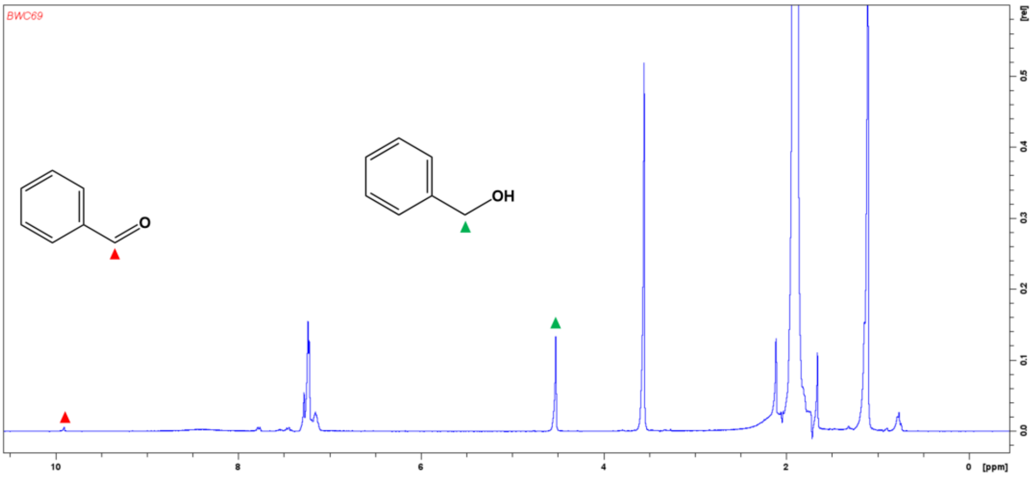

Figure S40 ^{1}H NMR spectra of post catalysis reaction solution catalysed by BA-Ce75.

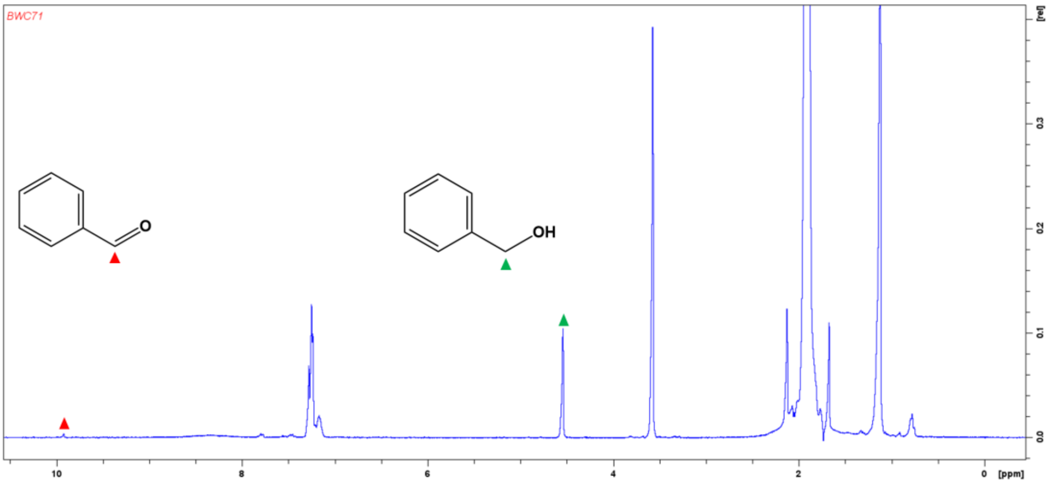

Figure S41 ^{1}H NMR spectra of post catalysis reaction solution catalysed by FA-Ce33.

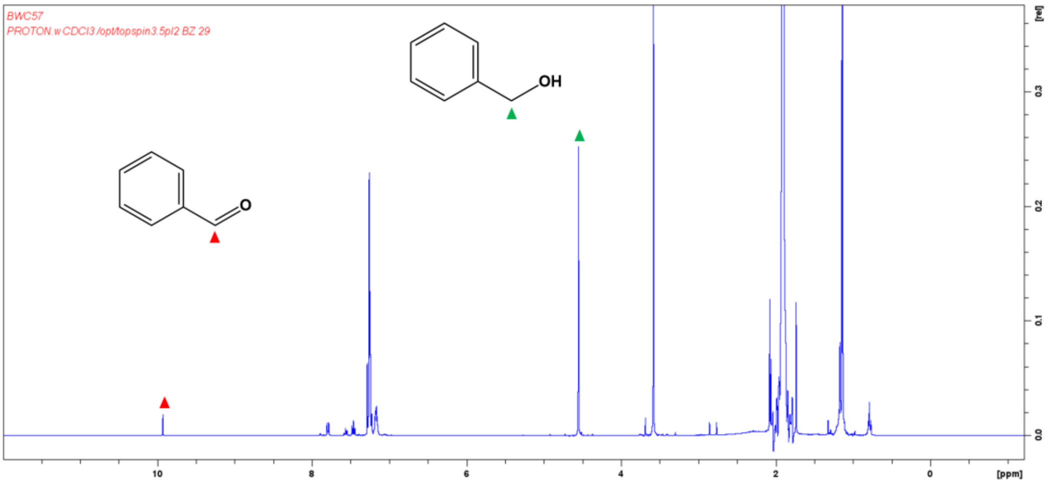

Figure S42 ^{1}H NMR spectra of post catalysis reaction solution catalysed by FA-Ce50.

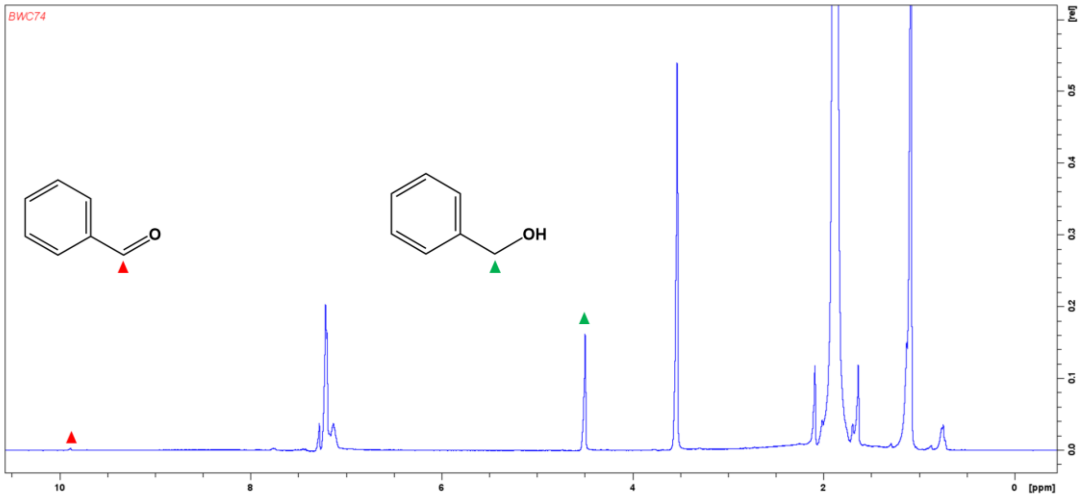

Figure S43 ^{1}H NMR spectra of post catalysis reaction solution catalysed by FA-Ce75.

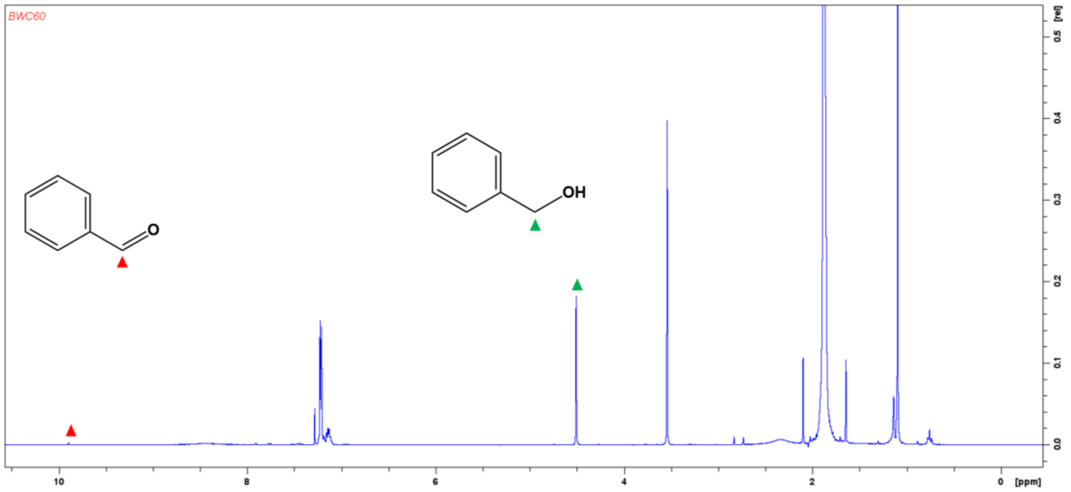

Figure S44 ^{1}H NMR spectra of post catalysis reaction solution catalysed by UiO-66(Ce).

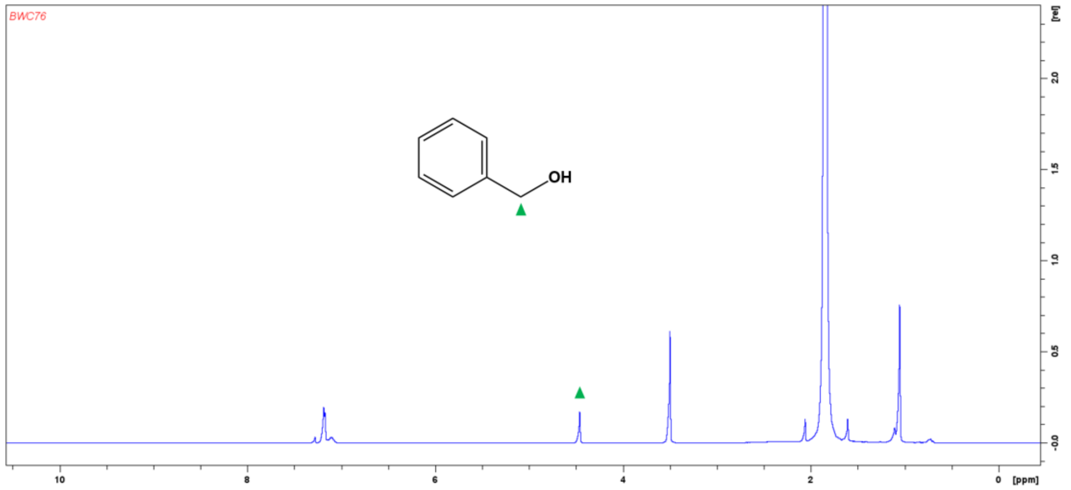

Figure S45 ^{1}H NMR spectra of post catalysis reaction solution catalysed by UiO-66(Zr).

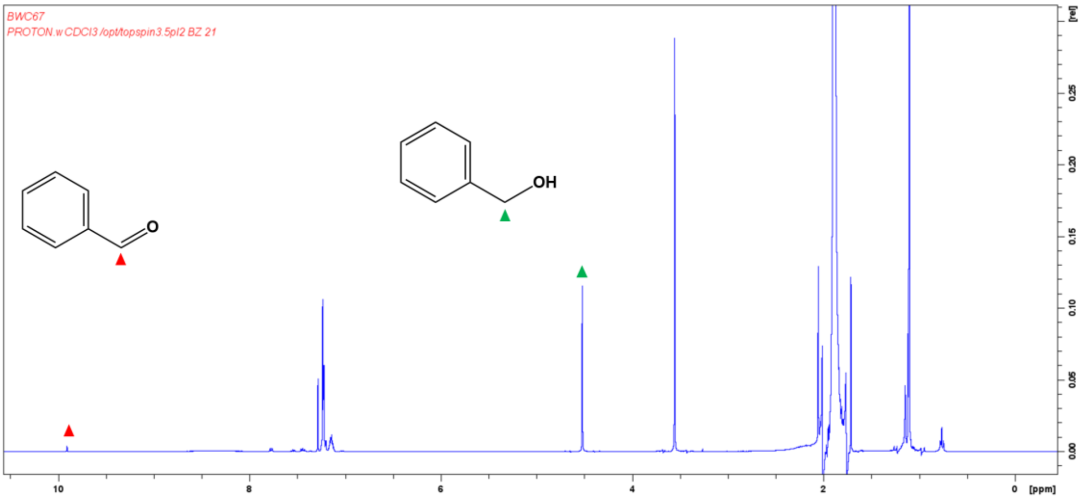

Figure S46 ^{1}H NMR spectra of post catalysis reaction solution catalysed by CeO_2.

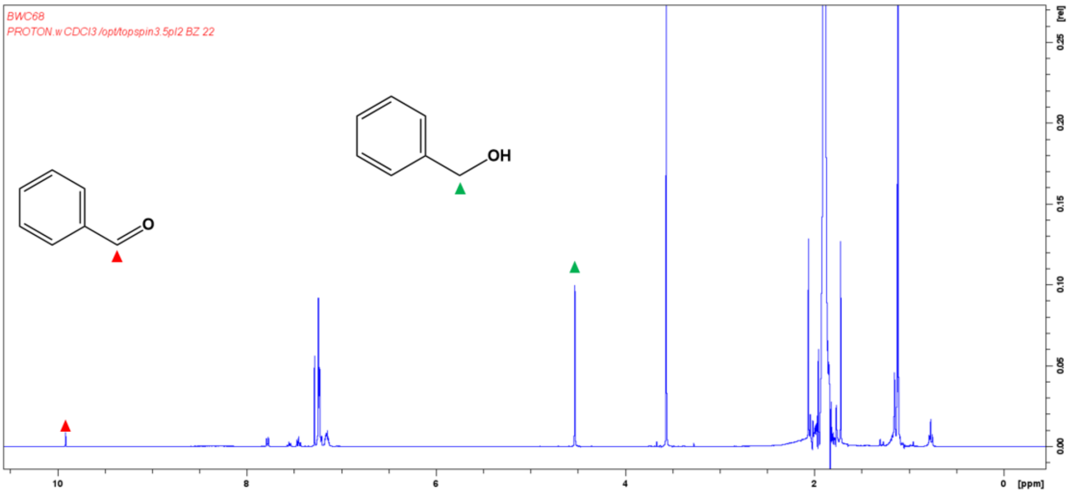

Figure S47 ^{1}H NMR spectra of post catalysis reaction solution catalysed by $Ce_{0.5}Zr_{0.5}O_2$.

S11. Density Functional Theory

S11.1 Computational details

The calculations in this work were carried out at the DFT level using CP2K. The PBE functional was used together with Grimme's D3 and three body semi-empirical corrections. Double-zeta MOLOPT gaussian primary basis sets and standard Goedecker-Tetter-Hutter (GTH) pseudopotentials were used for all atoms except cerium; a plane wave kinetic energy cutoff of 1200 Ry was used for the secondary basis set. For cerium, the basis set and pseudopotential of Lu *et al.* were used instead.[11] A Hubbard correction was applied to cerium's 4f shell with a single effective parameter U_{eff} of 7 eV. For systems containing Ce(III), the PBE0 functional

was instead used along with triple zeta MOLOPT basis sets. The Hartree-Fock contribution was determined with the auxiliary density matrix method (ADMM) for which Fit3 and Fit11 auxiliary basis sets were used for all atoms other than Ce. An auxiliary basis set developed for CeO_2 by Hahn *et al.* was used for cerium.[12] Other parameters remained unchanged.

During self-consistent field (SCF) cycles and structural optimisations, the following convergence criteria were enforced: 10^{-8}, 10^{-3}, 10^{-4}, 4.5×10^{-4}, and 4.5×10^{-4} (in atomic units) respectively for the energy, maximum step size, root mean square step size, maximum force, and root mean square force. The energy criteria was relaxed to 10^{-6} as needed for some calculations involving Ce(III) in which the SCF had difficulty converging. Aperiodic clusters and molecules were simulated inside an empty 25×25×25 $Å^3$ box to minimise interactions between periodic images. All *k*-point sampling was done at the gamma point given the large sizes of the unit cells involved.

S11.2 Isolated SBU calculations

In order to construct pure and mixed-metal isolated SBUs, a single SBU was initially cut from the optimised periodic structure of zirconium UiO-66. This cluster was then terminated exclusively with either formic or benzoic acid. Zr were then systematically substituted by Ce to generate all possible configurations. Structural optimisations were carried out without constraints.

The relative energy and free energy of a mixed-metal cluster can be defined via those of pure Zr_6 and Ce_6 clusters as:

$$\Delta E = E_{Zr_xCe_{6-x}} - \frac{x}{6}E_{Zr_6} - \frac{6-x}{6}E_{Ce_6} \qquad \text{(Equation S3)}$$

$$\Delta G = \Delta E - Tk_B \ln{(\Omega_i)} \qquad \text{(Equation S4)}$$

where Ω_i is the number of symmetry-related configurations for a given distribution *i* of cerium atoms in the mixed system. Note that as the reference states are phase-pure, their contributions to configurational entropy are 0. We neglect any vibrational contributions due to their associated computational cost (particularly for periodic systems, see below) but this is unlikely to make a significant difference. Given that the reference systems contain the same numbers and types of bonds as the mixed systems, the vibrational behaviour will not change significantly between the heterometallic clusters.

Schematics of some of the clusters modelled are also shown in Figure S48. The relative free energies have been calculated and are shown in Figure S49.

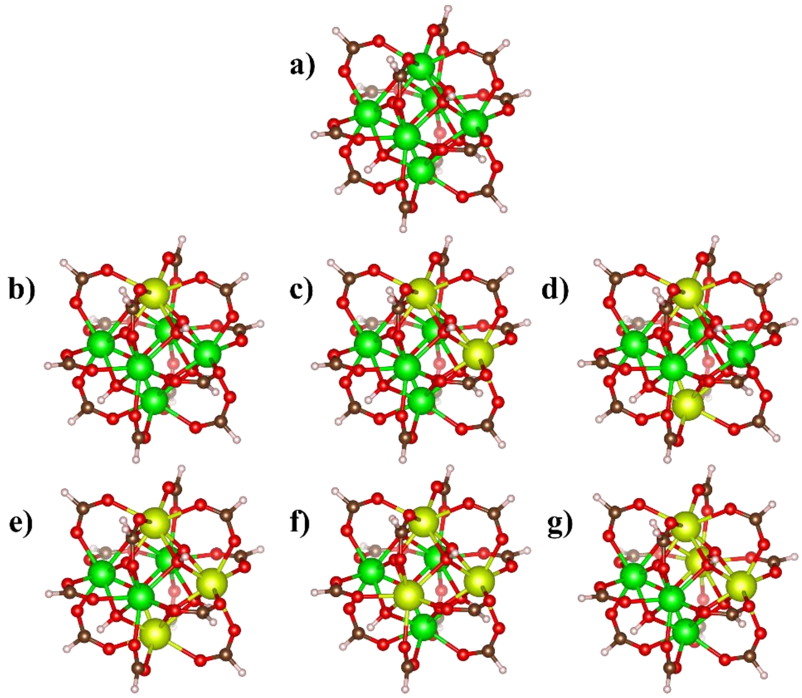

Figure S48 Schematics of some of the isolated clusters modelled in this work. Panels a-g, respectively, show clusters: Zr_6, Zr_5Ce_1, *cis*-Zr_4Ce_2, *trans*-Zr_4Ce_2, *mer*-Zr_3Ce_3, *fac*-Zr_3Ce_3-1, and *fac*-Zr_3Ce_3-2. Models f) and g) differ in whether the 3 cerium atoms surround a bridging oxygen or a bridging hydroxyl. H, C, O, Zr, and Ce atoms have been coloured white, brown, red, green, and yellow respectively.

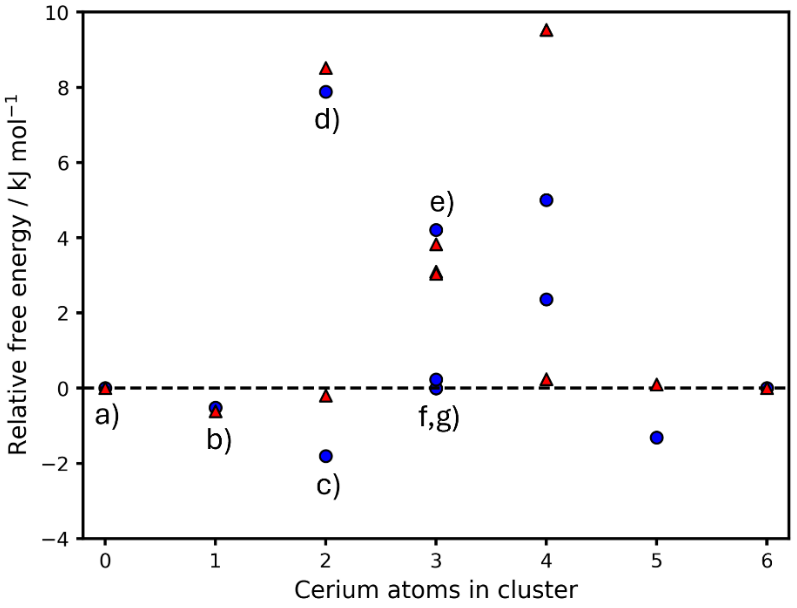

Figure S49 Relative energies of FA (red triangles) and BA (blue circles) terminated mixed-metal isolated M_6 clusters. The data points shown here are labelled according to the

corresponding panels in **Figure S48**. The models used for cerium contents 4-6 are analogous to panels a-d, but where every zirconium has been replaced by cerium and *vice versa*.

S11.3 Periodic simulations of Zr-Ce-UiO-66

The phase-pure Zr was optimised in its conventional unit cell from the experimental coordinates of Cavka *et al.*,[18] with all SBUs oriented in the same direction such that the symmetry is decreased from $Fm\bar{3}m$ to $F\bar{4}3m$. The Ce system was optimised after substituting all zirconium atoms for cerium. Both unit cells contain 4 SBUs and 24 metal ions. Mixed systems were constructed by substituting Zr for Ce in in the Zr-UiO-66 system followed by structural optimisation. A relative energy for the mixed system can then be defined in relation to the phase-pure end-states:

$$\Delta E = E_{Zr_xCe_{24-x}} - E_{Zr_{24}} - E_{Ce_{24}} \qquad \text{(Equation S5)}$$

Note that for the periodic material, we neglect the effect of configurational entropy because for a given distribution of cerium atoms, the number of symmetry-related distributions is relatively small (ranging from 1 to 48), such that the enthalpic contribution dominates.

Given 24 atoms in the conventional unit cell, the configurational space of possible mixed-metal frameworks becomes intractable past a handful of substitutions. 2 sequences representing opposing extremes have therefore been examined: one in which the average distance between cerium atoms is minimised, and one in which it is maximised. These are plotted respectively as blue circles and red triangles in figure 6 of the main manuscript and can be represented via the progressions below:

Minimum separation between cerium atoms:

$4Zr_6$ → $3(Zr_6)$-(Zr_5Ce_1) → $3(Zr_6)$-(*cis*-Zr_4Ce_2) → $3(Zr_6)$-(*fac*-Zr_3Ce_3) → $3(Zr_6)$-(*cis*-Zr_2Ce_4) → $3(Zr_6)$-(Zr_1Ce_5) → $3(Zr_6)$-(Ce_6) → $2(Zr_6)$-(Ce_6)-(Zr_5Ce_1) → $2(Zr_6)$-(Ce_6)-(*cis*-Zr_4Ce_2) → $2(Zr_6)$-(Ce_6)-(*fac*-Zr_3Ce_3) → $2(Zr_6)$-(Ce_6)-(*cis*-Zr_2Ce_4) → $2(Zr_6)$-(Ce_6)-(Zr_1Ce_5) → $2(Zr_6)$-$2(Ce_6)$ etc.

Maximum separation between cerium atoms:

$4(Zr_6)$ → $3(Zr_6)$-(Zr_5Ce_1) → $2(Zr_6)$-$2(Zr_5Ce_1)$ → (Zr_6)-$3(Zr_5Ce_1)$ → $4(Zr_5Ce_1)$ → $3(Zr_5Ce_1)$-(*trans*-Zr_4Ce_2) → $2(Zr_5Ce_1)$-2(*trans*-Zr_4Ce_2) → (Zr_5Ce_1)-3(*trans*-Zr_4Ce_2) → 4(*trans*-Zr_4Ce_2) → 3(*trans*-Zr_4Ce_2)-(*mer*-Zr_3Ce_3) → 2(*trans*-Zr_4Ce_2)-2(*mer*-Zr_3Ce_3) → *trans*-Zr_4Ce_2-3(*mer*-Zr_3Ce_3) etc.

For defective systems, 8 linkers have been removed in a regular arrangement such that the remaining structure has the bcu topology. This is intended only as a simple configuration of the defect structure given the intractable nature of the combined configurational space. For each linker removed from the structure, 2 formate anions have been introduced to terminate

the defective SBUs and maintain charge neutrality. The same metal substitutions have been made as for the pristine material.

Metal-metal distances have been tabulated for a subset of these structures for comparison with EXAFS fitting data; these can be found in Table S19. Unit cell volumes have also been tabulated as a function of cerium content, as shown in Figure S50.

Table S19 Ce-Ce distances for a selected subset of missed periodic unit cells. The minimum and average Ce-Ce distances both increase with increasing cerium content, but they remain smaller for the *cis*-Zr_4Ce_2 model than Zr_5Ce_1.

System	Ce – Ce distance / Å	
	Minimum	Average
$2Zr_6$-Ce_6-Zr_5Ce_1	3.766	3.782
$3Ce_6$-Zr_5Ce_1	3.781	3.793
$3Zr_6$-*cis*-Zr_4Ce_2	3.751	3.751
$3Ce_6$-*cis*-Zr_4Ce_2	3.770	3.793

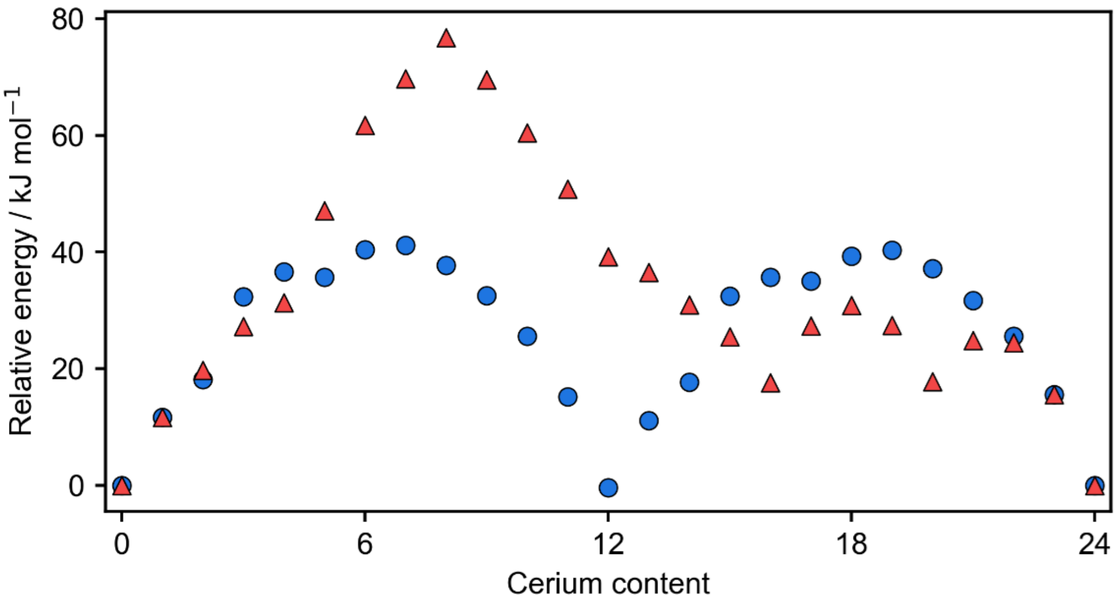

Figure S50 Relative energies of defective unit cells of mixed Zr-Ce UiO-66. Configurations of minimum average cerium separation in blue; maximum separation in red. The low-energy

configuration for 12 ceriums is such that SBU-linker bonds for Ce and Zr SBUs are completely decoupled, leading to 0 strain and minimal energy cost.

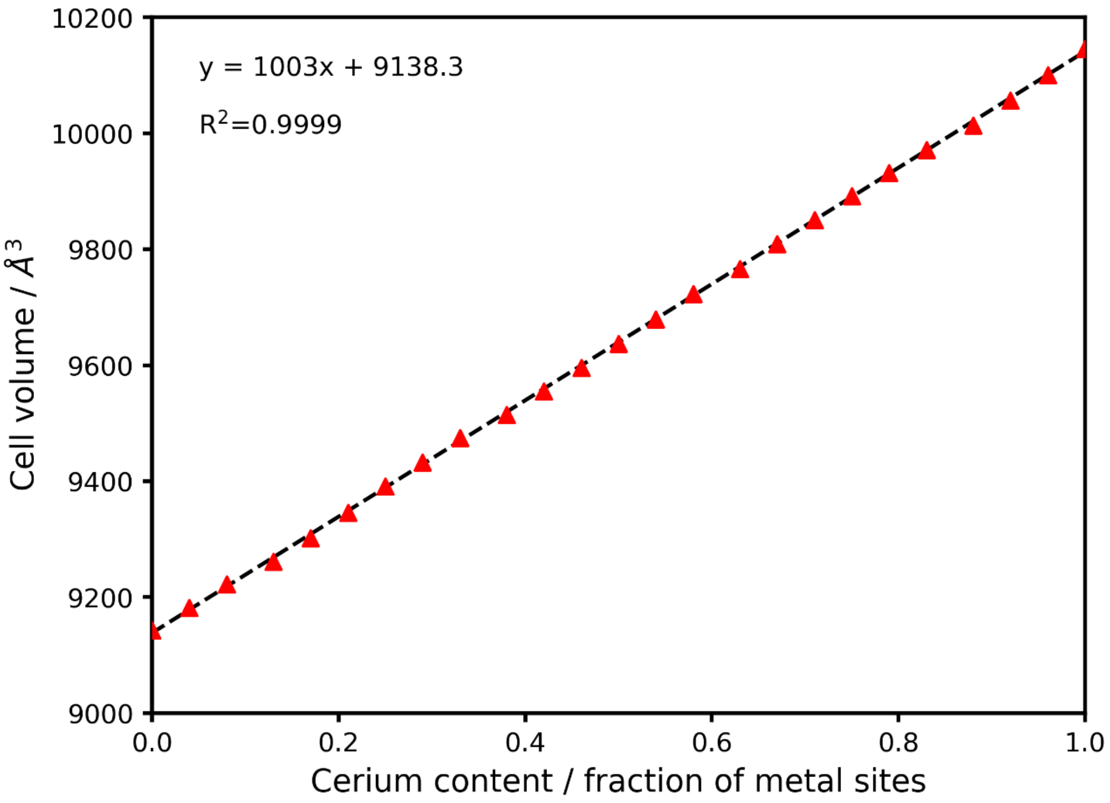

Figure S51 Volumes of optimised mixed UiO-66(Ce/Zr) systems with increasing cerium content. The volume for each cerium content has been determined as the average between the configuration with minimum Ce-Ce separations and the configuration with maximum Ce-Ce separation.

S11.4 Evaluating strain

Strain in individual bonds between a pair of atoms *i, j* was evaluated using the following definition:

$$\sigma = \frac{r_{i,j}(Zr,Ce) - r_{i,j}(reference)}{r_{i,j}(reference)} \times 100 \qquad \text{(Equation S6)}$$

where $r_{i,j}$ is the bond length, as found by DFT, between atoms *i* and *j* in either the mixed system or the suitable reference system. The reference system was defined as the pure UiO-66(Zr) system for bonds involving Zr and O as well as O—H bonds (which differ only minimally in the Zr and Ce materials), and pure UiO-66(Ce) for bonds involving Ce and O. Given that there is almost no spread in distances for bonds of the same type in UiO-66(Zr) and UiO-

66(Ce), individual bond lengths rather than averages were used for the reference. Note that we include here for completeness the Zr—Zr, Ce—Ce, and Zr—Ce distances which correspond to the edges of the M_6 octahedron as they provide additional information on the distortion of mixed SBUs relative to pure-metal ones. The reference Zr—Ce distance, given the lack of a suitable material for comparison, was set as the average of Zr—Zr and Ce—Ce distances in UiO-66(Zr) and UiO-66(Ce).

The bond lengths extracted from the DFT-optimised structures, which were used to evaluate the strains shown in Figure 8 of the main manuscript, are given in Table S21. The atom labels can be interpreted using Figure S52 and Table S20, the latter of which denotes the only changes in atom types between the different structures.

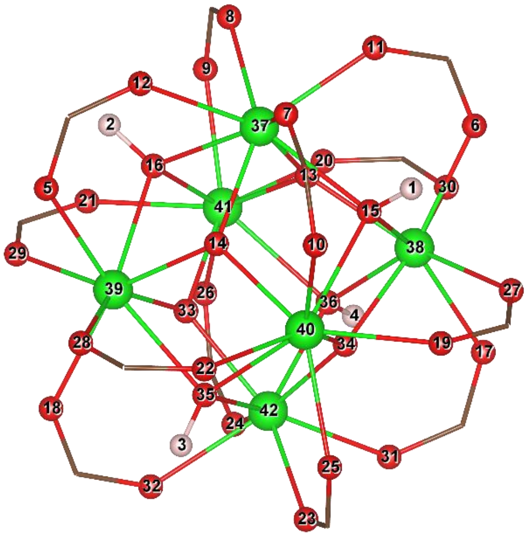

Figure S52 Schematic depicting atom labels for the bond lengths given in Table S21. Zr, O, and H atoms have been drawn in green, red, and white respectively. Note that only atom types 37-42 change between the systems analysed, as summarised in Table S20.

Table S20 Atom types which differ between UiO-66(Zr), UiO-66(Ce), and the mixed-metal systems

UiO-66(Zr)		UiO-66(Ce)	$3Zr_6$-Zr_5Ce_1	$3Zr_6$-*cis*-Zr_4Ce_2	$3Zr_6$-Ce_6
37	Zr	Ce	Zr	Zr	Ce
38	Zr	Ce	Zr	Zr	Ce
39	Zr	Ce	Zr	Ce	Ce
40	Zr	Ce	Ce	Ce	Ce
41	Zr	Ce	Zr	Zr	Ce
42	Zr	Ce	Zr	Zr	Ce

Table S21 Table of bond lengths for the selected structures in which strains were evaluated. All distances are given in Ångstrom; atom labels correspond to those given in Figure S52 and Table S20.

Label 1	Label 2	UiO-66(Zr)	UiO-66(Ce)	$3Zr_6$-Zr_5Ce_1	$3Zr_6$-*cis*-Zr_4Ce_2	$3Zr_6$-Ce_6
1	15	0.972	0.975	0.973	0.972	0.976
2	16	0.972	0.972	0.972	0.972	0.976
3	35	0.972	0.972	0.973	0.974	0.976
4	36	0.972	0.972	0.971	0.973	0.976
5	39	2.238	2.395	2.242	2.345	2.373
6	38	2.238	2.395	2.251	2.25	2.372
7	37	2.238	2.395	2.269	2.274	2.372
8	37	2.238	2.396	2.224	2.237	2.372
9	41	2.238	2.395	2.239	2.25	2.373
10	40	2.238	2.395	2.339	2.345	2.372
11	37	2.238	2.395	2.251	2.237	2.372
12	37	2.238	2.395	2.243	2.274	2.372
13	37	2.075	2.217	2.074	2.075	2.211
13	38	2.075	2.217	2.074	2.076	2.211
13	41	2.075	2.217	2.075	2.076	2.211
14	37	2.075	2.217	2.072	2.07	2.21
14	39	2.075	2.217	2.074	2.214	2.21
14	40	2.075	2.217	2.218	2.216	2.212
15	37	2.274	2.431	2.284	2.289	2.428
15	38	2.274	2.431	2.284	2.277	2.428
15	40	2.274	2.431	2.404	2.411	2.428
16	37	2.274	2.431	2.281	2.289	2.428
16	39	2.274	2.431	2.28	2.411	2.428
16	41	2.274	2.431	2.268	2.277	2.428
17	38	2.238	2.395	2.243	2.244	2.372
18	39	2.238	2.394	2.252	2.347	2.373
19	40	2.238	2.395	2.339	2.327	2.372
20	41	2.238	2.395	2.239	2.225	2.373
21	41	2.238	2.394	2.239	2.27	2.373
22	40	2.238	2.396	2.339	2.372	2.372
23	42	2.238	2.395	2.269	2.285	2.373
24	42	2.238	2.395	2.224	2.228	2.373
25	40	2.238	2.396	2.339	2.347	2.372
26	41	2.238	2.394	2.239	2.244	2.373
27	38	2.238	2.395	2.269	2.27	2.372
28	39	2.238	2.395	2.269	2.372	2.373
29	39	2.238	2.395	2.224	2.328	2.373
30	38	2.238	2.396	2.224	2.226	2.372
31	42	2.238	2.395	2.242	2.228	2.373

32	42	2.238	2.394	2.252	2.287	2.373
33	39	2.075	2.217	2.076	2.219	2.211
33	41	2.075	2.217	2.075	2.072	2.211
33	42	2.075	2.217	2.076	2.072	2.211
34	38	2.075	2.217	2.072	2.072	2.211
34	40	2.075	2.217	2.218	2.219	2.211
34	42	2.075	2.217	2.074	2.072	2.211
35	39	2.274	2.431	2.285	2.413	2.428
35	40	2.274	2.431	2.404	2.413	2.428
35	42	2.274	2.431	2.285	2.287	2.428
36	38	2.274	2.431	2.28	2.274	2.428
36	41	2.274	2.431	2.269	2.274	2.428
36	42	2.274	2.431	2.281	2.285	2.428
37	38	3.541	3.796	3.55	3.554	3.783
37	39	3.541	3.796	3.553	3.668	3.783
37	40	3.541	3.796	3.654	3.668	3.783
37	41	3.541	3.796	3.542	3.554	3.783
38	40	3.541	3.796	3.654	3.656	3.783
38	41	3.541	3.796	3.542	3.544	3.783
38	42	3.541	3.796	3.553	3.553	3.783
39	40	3.541	3.796	3.656	3.751	3.783
39	41	3.541	3.796	3.543	3.656	3.783
39	42	3.541	3.796	3.553	3.664	3.783
40	42	3.541	3.796	3.656	3.664	3.783
41	42	3.541	3.796	3.543	3.553	3.783

S11.5 Solution-state Ce(III) complexes

In order to evaluate whether it is possible for Ce(III) to participate in the formation of M_6 clusters, a number of reference solution-state complexes have been modelled. In each case 3 anions are chosen between nitrate, chloride, formate, or benzoate (all expected to be present in solution via the precursor or modulator) and 6 molecules of DMF are added to fill the coordination sphere. Relative energies have been defined by setting the lowest absolute energy to 0, making it clear which one is most stable.

Table S22 Ce(III) solution-state complexes and their relative energies

NO_3^-	Cl^-	FA^-	BA^-	DMF	Relative energy / kJ mol^{-1}
3	0	0	0	6	0
2	0	1	0	6	36.2

1	0	2	0	6	100.8
0	0	3	0	6	107.0
2	0	0	1	6	25.5
1	0	0	2	6	56.5
0	0	0	3	6	63.3
0	3	0	0	6	31.7
0	2	1	0	6	69.5
0	1	2	0	6	110.0
0	2	0	1	6	48.4
0	1	0	2	6	64.7

To model M_6 clusters containing Ce(III), we started from the FA and BA terminated clusters constructed in S11.2. For each cerium to be reduced to 3+, a proton was added to an adjacent bridging oxygen on the SBU, and the structure reoptimized with the Unrestricted Kohn-Sham (UKS) formalism. Thereafter, the relative energies of such clusters compared to pure Zr_6 ones and Ce(III) complexes can be defined through the following balanced equation:

$$\Delta E = E_{Zr_xCe_{6-x}} - \frac{x}{6}E_{Zr_6} - (6-x)E_{Ce(III)} - \frac{4x}{3}E_{H_2O} + 6xE_{DMF} + 3xE_{HNO_3} - 2xE_{acid}$$

(Equation S7)

The most stable cluster containing 3 nitrates and 6 DMF has been chosen as reference, and some additional water is needed for balancing. $E_{Ce(III)}$ is therefore the absolute energy of the corresponding 3-nitrate,6-DMF complex. E_{acid} here refers to the appropriate termination (FA or BA) for the given cluster. ΔE for some FA and BA-terminated SBUs is shown in Table S23:

Table S23 Energy cost of including Ce(III) in SBUs compared to forming complexes and remaining in solution

System	Number of Ce(III)	Relative energy / kJ mol^{-1}
Zr5Ce1 – FA	1	303.7
Zr4Ce2 – FA – cis	2	585.7
Zr4Ce2 – FA - trans	2	619.4
Zr5Ce1 – BA	1	287.2
Zr4Ce2 – BA - cis	2	560.1
Zr4Ce2 – BA - trans	2	586.1

S11.6 2-electron oxidation of M_6 clusters

The ease with which different M_6 clusters can undergo reduction can be evaluated to rationalise variations in catalytic activity. The energy cost of 2-electron reduction via the loss of an oxygen atom can be estimated via:

$$\Delta E = E_{Zr_xCe_{6-x}O_7H_4} + \frac{1}{2}E_{O_2} - E_{Zr_xCe_{6-x}O_8H_4} \quad \text{(Equation S8)}$$

where terms on the left-hand side correspond respectively to energies of an open shell cluster with a bridging oxygen adjacent to the cerium atoms removed, an isolated oxygen molecule in its triplet configuration, and the pristine cluster. Given the large size of the unit cell and the cost of the hybrid functional used, aperiodic models have been used. These have been constructed by extracting an SBU and its 12 adjacent linkers from the periodic material and converting these to benzoates. These were held fixed during optimisation to mimic the rigidity offered by the framework in the true material. One benzoate near cerium was replaced by a hydroxide anion as shown in the first step of Figure 9 of the main manuscript.

References

(1) Lammert, M.; Glissmann, C.; Stock, N. Tuning the Stability of Bimetallic Ce((IV))/Zr((IV))-based MOFs with UiO-66 and MOF-808 Structures. *Dalton Trans.* **2017**, *46* (8), 2425-2429.
(2) Toby, B. H.; Von Dreele, R. B. *GSAS-II*: The Genesis of a Modern Open-source All Purpose Crystallography Software Package. *J. Appl. Crystallogr.* **2013**, *46*, 544-549.
(3) Hennig, C.; Ikeda-Ohno, A.; Kraus, W.; Weiss, S.; Pattison, P.; Emerich, H.; Abdala, P. M.; Scheinost, A. C. Crystal Structure and Solution Species of Ce(III) and Ce(IV) Formates: From Mononuclear to Hexanuclear Complexes. *Inorg. Chem.* **2013**, *52* (20), 11734-11743.
(4) Bailey, M.; Brown, C. J. The crystal structure of terephthalic acid. *Acta Crystallogr.* **1967**, *22* (3), 387-391.
(5) Brown, S. D.; Bouchenoire, L.; Bowyer, D.; Kervin, J.; Laundy, D.; Longfield, M. J.; Mannix, D.; Paul, D. F.; Stunault, A.; Thompson, P.; et al. The XMaS Beamline at ESRF: Instrumental Developments and High-resolution Diffraction Studies. *J. Synchrotron Radiat.* **2001**, *8* (6), 1172-1181.
(6) Klementiev, K.; Chernikov, R. XAFSmass: a program for calculating the optimal mass of XAFS samples. *J. Phys.: Conf. Ser.* **2015**, *712*, 012008.

(7) Ravel, B.; Newville, M. *ATHENA*, *ARTEMIS*, *HEPHAESTUS*: Data Analysis for X-ray Absorption Spectroscopy Using *IFEFFIT*. *J. Synchrotron Radiat.* **2005**, *12*, 537-541.
(8) Trousselet, F.; Archereau, A.; Boutin, A.; Coudert, F. X. Heterometallic Metal-Organic Frameworks of MOF-5 and UiO-66 Families: Insight from Computational Chemistry. *J. Phys. Chem. C* **2016**, *120* (43), 24885-24894.
(9) Ambroz, F.; Macdonald, T. J.; Martis, V.; Parkin, I. P. Evaluation of the BET Theory for the Characterization of Meso and Microporous MOFs. *Small Methods* **2018**, *2* (11), 1800173.
(10) Hasegawa, T.; Shahed, S. M. F.; Sainoo, Y.; Beniya, A.; Isomura, N.; Watanabe, Y.; Komeda, T. Epitaxial growth of CeO₂(111) film on Ru(0001): Scanning tunneling microscopy (STM) and x-ray photoemission spectroscopy (XPS) study. *J. Chem. Phys.* **2014**, *140* (4).
(11) Lu, J.-B.; Cantu, D. C.; Nguyen, M.-T.; Li, J.; Glezakou, V.-A.; Rousseau, R. Norm-Conserving Pseudopotentials and Basis Sets To Explore Lanthanide Chemistry in Complex Environments. *J. Chem. Theory. Comput.* **2019**, *15* (11), 5987-5997.
(12) Hahn, K. R.; Iannuzzi, M.; Seitsonen, A. P.; Hutter, J. Coverage Effect of the CO_2 Adsorption Mechanisms on CeO_2(111) by First Principles Analysis. *J. Phys. Chem. C* **2013**, *117* (4), 1701-1711.
